# Effects of Patch Size, Fragmentation, and Invasive Species on Plant and Lepidoptera Communities in Southern Texas

**DOI:** 10.3390/insects12090777

**Published:** 2021-08-29

**Authors:** James A. Stilley, Christopher A. Gabler

**Affiliations:** 1 School of Earth, Environmental, and Marine Sciences, University of Texas Rio Grande Valley, 1 West University Blvd, Brownsville, TX 78520, USA; jimmystilley@verizon.net; 2 Department of Biology, University of Texas Rio Grande Valley, 1201 W University Dr, Edinburg, TX 78539, USA

**Keywords:** habitat loss, fragmentation, biodiversity, landscape ecology, conservation, wildlife, management, Rio Grande Valley, Tamaulipan thornscrub

## Abstract

**Simple Summary:**

Human land use has removed habitats, separated habitats into small and disconnected fragments, and introduced foreign species, which all harm wildlife. South Texas is highly diverse and home to many endangered species, but human disturbance threatens its wildlife. In south Texas, we poorly understand how different aspects of human land use influence wildlife diversity and abundance. We studied this by surveying plants and butterflies in 24 habitat fragments in south Texas that differed in size, shape, type, and land use history. Human disturbance was extensive, and foreign and weedy species were dominant in most habitats. Habitat types had distinctive sets of plants and butterflies, but habitats with the most human disturbance were the least distinct and had the most foreign or weedy species. Usually, larger and less-fragmented habitats have fewer foreign and weedy species and have higher diversity, and habitats with more foreign and weedy species have lower diversity, but only the first of these was true in our study. This suggests that historic sets of native plants are very rare, most areas are actively recovering from disturbance, and foreign species are now a normal part of communities. This study helps us understand how human land use impacts wildlife and how we can better manage land to protect and enhance wildlife.

**Abstract:**

Habitat loss, fragmentation, and invasive species are major threats to biodiversity. In the Lower Rio Grande Valley (LRGV) of southern Texas, a conservation hotspot, few studies have examined how land use change and biotic disturbance influence biodiversity, particularly among Lepidoptera. We surveyed 24 habitat fragments on private lands in the LRGV and examined how patch size, edge to interior ratio (EIR), prevalence of invasive, exotic, and pest (IEP) plant species, and other environmental factors influenced plant and Lepidoptera communities within four habitat classes. Biotic disturbance was widespread and intense. IEP plants represented three of the four most common species in all but one habitat class; yet, classes largely had distinctive plant and Lepidoptera communities. Larger habitat patches had lower IEP prevalence but also lower plant richness and lower Lepidoptera richness and abundance. Conversely, patches with higher EIRs had greater IEP prevalence, plant richness, and Lepidoptera richness and abundance. IEP prevalence was negatively related to plant diversity and positively related to woody dominance, blooming plant abundance, and, surprisingly, both plant cover and richness. However, plant richness, abundance, and diversity were higher where a greater proportion of the plants were native. Lepidoptera diversity increased with plant cover, and Lepidoptera richness and abundance increased with plant richness. More individual Lepidoptera species were influenced by habitat attributes than by availability of resources such as host plants or nectar sources. Our results illustrate extensive landscape alteration and biotic disturbance and suggest that most regional habitats are at early successional stages and populated by a novel species pool heavy in IEP species; these factors must be considered together to develop effective and realistic management plans for the LRGV.

## 1. Introduction

### 1.1. Background and Context

The emerging global trend in land management is to assess conservation goals and objectives at a landscape scale [1]. This trend comes from the realization that historical methods of managing wildlife and natural resources on small scales, such as within single preserves, without considering the area surrounding and between other preserves, is inadequate for long-term species protection and survival [1]. This revision in conservation strategy comes at an important time because landscapes worldwide are rapidly being altered to facilitate growing human populations [2]. Many nations such as the United States, for the first time in history, have equal proportions of land classified as wilderness (5%) and urban (4%) [3]. Conservationists have stated that one of the greatest conservation challenges of the modern era will be to understand how anthropogenic disturbance affects biodiversity [4].

Landscape alterations to facilitate anthropogenic expansion have created what are arguably the two largest global problems in conservation biology: habitat loss and habitat fragmentation [5]. Habitat loss and fragmentation occur when continuous natural landscapes are modified and transformed into islands of relatively undisturbed land surrounded by a matrix of land that is less hospitable for wildlife, such as urban areas and farmland [6]. Both habitat loss and habitat fragmentation are known to have profound impacts on ecosystems and species survival [5]. However, recent research has discovered that certain types of wildlife are utilizing human-disturbed landscapes more than expected and, in some instances, these modified landscapes even support high levels of diversity for specific taxa [4]. Other studies have found that the human-disturbed habitat matrix can contain critical resource features that have a substantial influence on connected ecosystems; this includes both natural features (e.g., flowering meadows or artesian springs) and artificial features (e.g., butterfly gardens or irrigation canals) [7].

As a result, understanding how wildlife are utilizing these novel landscapes is an important area of research for both land managers and conservationists [8]. A realistic approach to understanding how landscapes and their features influence wildlife is to (a) split a landscape into distinguishable components or patches; (b) differentiate between patches by considering relevant categories (such as habitat types) and continuous habitat characteristics (such as patch size, degree of fragmentation, and level of disturbance); and then (c) assess differences between these groups and across these gradients and evaluate how these factors influence wildlife [9,10,11]. This approach is central to the current study.

However, resource managers and decision makers face another problem, which is that researchers and land managers typically do not have enough time or resources to thoroughly examine every aspect of an ecosystem, and detailed data are seldom available from before landscapes were extensively altered by invasive species or land conversion [10,12]. In response, a variety of streamlined methodologies (often termed ‘rapid assessment methods’) have been developed that can quickly and effectively assess habitat attributes and wildlife usage across landscapes by quantifying a relatively small set of relatively easy to measure variables that serve as reliable indicators of broader ecological or environmental conditions [13]. To address the grander challenge of extensive human alteration of landscapes, many land managers are focusing on attempts to restore functional connectivity of landscapes by preserving or enhancing ecosystem function within strategic habitat patches (e.g., creating conservation corridors), rather than unrealistic and typically futile attempts to restore entire landscapes to historic conditions [12]. This change in strategy came partly from the understanding that species, especially during migration, utilize secondary habitat as temporary refugia and that connectivity is obtainable even if parts of a focal area consist of lower quality habitat [14]. 

Biodiversity is central to these considerations for many resource managers. Rapid global decline in biodiversity is one of the great challenges facing humanity. Biodiversity is now considered a sort of currency among natural resources managers because it provides an ‘insurance policy’ against climate change, secures the existence of many raw materials, provides valuable ecosystem services, and directly supports rising ecotourism industries globally [15]. Unfortunately, the complexity of biodiversity creates challenges for researchers and land managers trying to develop cost- and time-efficient biodiversity monitoring or assessment schemes for wildlife in their focal areas [16]. In response, the idea of indicator taxa arose and was validated decades ago [17], but land managers continue to struggle to identify the best indicator taxa for assessments of specific management goals and objectives [18,19]. 

This study investigated biodiversity in south Texas, which remains very high despite extensive human land use change, and aimed to quantify landscape-scale relationships between habitat attributes and wildlife abundance and diversity. We focused specifically on private lands that might be useful for a future conservation corridor, even though human impacts are likely high compared to nearby protected lands. After carefully considering butterflies [20] and other arthropod groups [18,19] as candidate indicator taxa, we chose to assess all Lepidoptera. 

Butterflies and other Lepidoptera taxa are widely considered good biodiversity indicators. Butterflies are a well-studied taxa [18,21], and, in most areas, the assemblage of species in a butterfly community is both large and diverse enough to represent the unique features of a landscape, yet also small enough for land managers to sample and assess in a time-efficient manner [9]. Diversity of butterflies and other Lepidoptera has been directly linked to Hymenoptera diversity [10], plant diversity [3,22], and to the degree of urbanization [3]. Inventories of butterfly abundance and diversity have been effectively used to classify and assess habitats and to evaluate land conditions [23,24]. Furthermore, Lepidoptera diversity and community structure are typically correlated with many ecological attributes, including habitat complexity, climate variability, moisture gradients, temperature regimes, and topography [23,24], so Lepidoptera can serve as reliable indicators of environmental conditions or change. Lepidoptera, especially the butterflies and skippers, are ideal for monitoring programs because they are relatively easily identifiable by non-specialists (e.g., citizen scientists, land managers, and field technicians), and their well-documented ecology makes it simple for researchers to link their presence to other ecological patterns, such as host plant abundance or seasonal vegetation phenology [4,12]. Finally, researchers have determined that even short-term butterfly studies can provide enough data for land managers to make informed decisions [4].

### 1.2. Rationale and Related Research

The Lower Rio Grande Valley (LRGV) of southernmost Texas is a conservation hotspot because it has high biodiversity, is home to several endangered species, and is under great pressure from a rapidly growing human population and associated land use changes [25]. We focused specifically on private land, which is broadly underrepresented in ecological studies relative to the proportion of the landscape it occupies in most regions. In Texas, 95.8% of land is privately owned, and only five other U.S. states have a lower percentage of public land [26]. The LRGV is also home to three large National Wildlife Refuges (NWRs) managed by the U.S. Fish and Wildlife Service (USFWS), specifically Laguna Atascosa NWR, Santa Ana NWR, and the Lower Rio Grande Valley NWR. Development of a conservation corridor system linking these NWRs and other protected lands has been a regional conservation goal for the last thirty years. Typically, corridors connecting protected areas must be constructed through a matrix of private lands, yet the ecological status of private lands are often the least well documented or understood. 

Data were collected in a single sampling period in November and December 2018 during the annual fall butterfly migration, which lasts approximately 2.5 months in the LRGV. Researchers have found that short-term studies such as this, despite having many limitations, can provide enough reliable and actionable data for land managers to develop informed management decisions [4]. To increase our ability to detect meaningful ecological patterns, we adopted a more conservative approach by broadening our focal taxa from butterflies to include all taxa in the order Lepidoptera. Upscaling to Lepidoptera is feasible in the LRGV because butterflies, skippers, and moths of the Tamaulipan biotic province (a larger area that includes the LRGV of Texas and northeastern Mexico) is thoroughly described [27,28,29,30]. However, although the regional assemblage of Lepidoptera species in the LRGV and the basic ecology of these species are well studied [31,32,33,34,35,36], there is a large knowledge gap in regard to the landscape and community ecology of Lepidoptera in the LRGV and the interactions between Lepidoptera and other taxa. To the best of our knowledge, there are no published studies on Lepidoptera community ecology in south Texas, other than species lists compiled for regional field guides and visitor checklists (e.g., those available at parks and wildlife refuges). 

Despite longstanding knowledge that Lepidoptera and plant communities are linked, investigations into the subtleties of this linkage, especially in light of anthropogenic modification of landscapes, is only a recent area of research [3,37]. Previous studies have produced nuanced and sometimes mixed results regarding the effects of anthropogenic habitat modification on Lepidoptera communities. Most studies have found that, although some Lepidoptera species benefit, human impacts are generally detrimental to more species and to overall Lepidoptera richness, abundance, and diversity [3,23,37,38,39,40]. However, some studies have documented positive effects of human impacts of Lepidoptera communities, for example, when introduced vegetation provides more attractive nectar sources and can cause host plant switches in butterflies [23]. Nevertheless, negative consequences of human landscape modification abound and are most often related to increased prevalence of invasive plants and declines in native plant species richness, abundance, and/or diversity, which are needed to support healthy populations of native Lepidoptera species [3,23,37,38,39,40]. 

The LRGV is an exemplary location to study linkages between Lepidoptera and plant communities and the impacts of anthropogenic landscape modification on both. First, the LRGV has the highest butterfly diversity in the United States and is located on the path of one of the largest butterfly migration routes in North America [41]. Despite this high diversity, most Lepidoptera species in the LRGV and their ecology are well known and thoroughly described [41]. Butterfly ecotourism is of major economic importance regionally and complements the globally recognized birding ecotourism that is already well-established in the LRGV [42]. Currently, ecotourism generates $59–$300 million per year in the LRGV, and ecotourism in the LRGV is growing at a similar level as the global average which is around 10–30% annually [42,43]. Elucidating relationships between pollinator species and human land use change is regularly touted as important and urgent, and this is particularly true in agriculture-heavy regions such as the LRGV [44]. Furthermore, native plant communities in the LRGV have high conservation value and are ecologically and economically important in their own right, yet are also understudied [41]. There is growing public interest and participation in regional conservation, in part due to the emergence of carbon credit markets and new subsidies for landowners that promote conservation of native wildlife and their habitats [41,45]. Lastly, the LRGV has one of the fastest urbanization rates in the USA, making the needs to understand human impacts and safeguard remaining habitats and biodiversity all the more urgent [41,45]. 

Like many parts of North America, the LRGV has a long history of human modification, especially since the arrival of Europeans in the 1700s [46]. Land modification occurred in distinct phases, starting with overgrazing by sheep in the 18th and 19th centuries [41], hydrologic changes to the Rio Grande river in the 20th century [41], agricultural land expansion and habitat clearing in the 1950s and 1960s [46], and the introduction of numerous Old World grasses in the late 19th and 20th centuries, including *Sorghum halepense* (Johnson grass), *Cynodon dactylon* (Bermuda grass), *Pennisetum ciliare* (buffelgrass), *Urochloa maxima* (Guinea grass), and *Dichanthium annulatum* (Kleberg bluestem), which are now invasive and threaten herbaceous species that account for 75–80% of the plant diversity in the LRGV [46,47]. These modifications resulted in a 95% reduction in native thornscrub forests, a highly diverse habitat type unique to the Tampaulian biotic province, and major regional losses of highly diverse Gulf Coastal prairies, which occur from south Texas to eastern Louisiana but have been reduced from covering 3.8 million ha to only ca. 3800 ha across its entire range. 

Tamulipan thornscrub forests (or thornforest) and shrublands are particularly important habitat types for land managers in the LRGV. Thornforests are preferred habitat for many native reptiles and mammals, including endangered ocelots (*Leopardus pardalis albescens*), which require closed-canopy thornforests with >95% cover [48,49,50]. Thornforests also provide forage and habitat for a diverse and abundant assemblage of bees, beetles, resident and migratory birds and butterflies, and many other organisms, especially when flowering [51].

The importance of thornforests as habitat for regional wildlife, especially ocelots, and the benefits they provide via carbon storage, combined with their historic rate of loss and current threatened status, has made conservation and restoration of thornforests in the LRGV a national and regional priority among USFWS and various NGOs, such as The Nature Conservancy and American Forests. Similarly, accelerating efforts to reconnect high-quality habitat patches in the LRGV via conservation corridors have made both the ecological assessment of lands not currently under federal or NGO ownership and the development of protocols for reliably and efficiently doing so into major conservation priorities in the LRGV region.

### 1.3. Objectives and Hypotheses

This study assessed the impacts of habitat alteration and fragmentation on biodiversity and ecological conditions at a landscape scale in the LRGV by examining plant and Lepidoptera communities at 24 study sites located on private land. The specific study objectives were to (1) quantify plant and Lepidoptera community structure on private lands (that might be useful for future conservation corridors) across a range of habitat types, sizes, and conditions; (2) examine the relationships between wildlife communities and habitat type, patch size, fragmentation, and the prevalence of invasive, exotic, and pest (IEP) plant species; and (3) examine the relationship between plant and Lepidoptera communities. As part of our third objective, we also investigated how the abundances of individual Lepidoptera species were influenced by the availability of particular resources, specifically host plants and nectar sources.

Based on prior studies, we expected higher levels of human disturbance would be associated with decreased plant community diversity or complexity, and that both of these would be associated with reduced Lepidoptera abundance and/or diversity [3,4,8,37,38]; however, intermediate levels of disturbance may demonstrate higher plant and Lepidoptera diversity and/or abundance [3,8]. We also expected physical attributes of habitats (patch size, edge to interior ratio) [52], structural differences (woody species prevalence) [8], and the prevalence of native versus IEP plant species to influence community composition [40,53]. 

## 2. Materials and Methods

### 2.1. Study Site Selection

**Figure 1:**
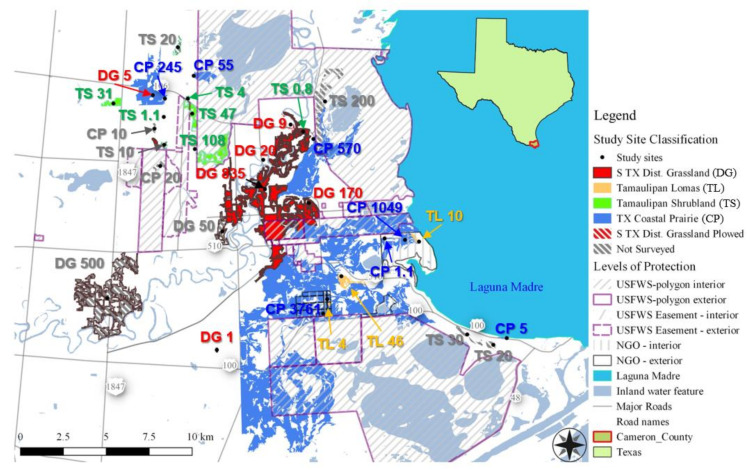
Study sites used for this investigation. Colored polygons represent the spatial extent of habitat patches of different geospatial classes. See Methods for classification details. Numbers after the 2-letter site classification codes denote patch size in ha. Also shown are polygons with different hashing to represent relevant land ownership categories (USFWS, private with a USFWS conservation easement, and conservation-oriented NGO), which confer different levels of habitat protection and permit different types of land use. Land not bounded by a hashed protection polygon is privately owned without any conservation easements.

Plant and Lepidoptera surveys were performed at 24 study sites located across 13 private ranches and farms in the vicinity of the Laguna Atascosa National Wildlife Refuge (NWR) in southern Texas (Figure 1). All study sites are in northeastern Cameron County, Texas, which is bordered by the Gulf of Mexico and falls on the international boundary with Mexico. Cameron County is part of the Lower Rio Grande Valley (LRGV) region, a well-known transition zone between temperate and tropical climates, which contributes to its high species diversity [45]. The LRGV is home to 19 federally threatened and endangered species and 60 state listed species, with some of the most iconic being *Leopardus pardalis albescens* (northern ocelot), *Puma yagouaroundi cacomitli* (Gulf Coast jaguarundi), and *Falco femoralis septentrionalis* (northern Aplomado falcon) [45].

Laguna Atascosa NWR encompasses 36,359 hectares of saline coastal prairie, freshwater wetlands, tidal flats, sand dunes, and thornscrub shrublands and forests [45]. Private ranches and farms in the vicinity have the same habitats found on the refuge, but with higher levels of human disturbance. The amount of protected native habitat on nearby private lands and the level of protection vary, with some habitat patches entirely in a conservation easement, some being restored voluntarily by landowners, and others with no formal protection. Many of the nearby private lands are actively being used for agricultural production (farmland or rangeland) and have been heavily altered. As discussed above, the LRGV has experienced over a 95% reduction in native thornscrub forest habitat, comparable losses of native grasslands, and a major reduction in riparian forests, and this habitat loss and the resulting habitat fragmentation are the primary reasons for the decline of many species found in the LRGV [45]. 

The original study design was based on recommendations of local experts (wildlife biologists from the USFWS South Texas Refuge Complex) to simplify the region’s habitat diversity into two main habitat types (forest or grassland; or, more accurately, woody- or herbaceous-dominated) and two classes of human land use history (“pristine” or disturbed). This approach guided our site selection, which also aimed to capture a gradient of habitat patch sizes, but initial analyses showed that both woody plant prevalence and the level of human disturbance were highly variable within categories, and that both factors were better represented by a continuous gradient rather than a relatively arbitrary distinction between two categories (see below). Habitat categorization is very important, however, both legally and due to its heavy use in geospatial analyses, so we still considered habitat types and defined four classes based on the Texas Mapping System (TMS) habitat classifications. 

Suitable study sites were identified using published geospatial habitat classification data accessed via the Texas Ecosystem Analytical Mapper (TEAM) website and limited to habitat designations present in the focal area [54]. For this study, ‘Tamaulipan Shrubland’ (TS) sites consisted of the following NatureServe and TMS ecosystem classifications described by Elliot [55]: ‘Tamaulipan mixed deciduous thornscrub’ (CES301.983), subclasses ‘South Texas: Clayey Mesquite Mixed Shrubland’ (7004), and ‘South Texas: Clayey Blackbrush Mixed Shrubland’ (7005); and ‘Tamaulipan Savanna Grassland’ (CES301.985), subclasses ‘South Texas: Sandy Mesquite Woodland and Shrubland’ (7104), and ‘South Texas: Sandy Mesquite Dense Shrubland’ (7105). ‘Texas Coastal Prairie’ (CP) sites included the ecosystem classifications of ‘Texas saline coastal prairie’ (CES203.543), subclass ‘Gulf Coast: Salty Prairie’ (2207). ‘Tamaulipan Lomas’ (TL) sites included the classifications ‘Tamaulipan lomas’ (CES301.462), subclass ‘South Texas: Loma Evergreen Shrubland’ (7305). ‘South Texas Disturbed Grassland’ (DG) sites included ‘South Texas: disturbance grassland’ (TMSID 9187). 

To select among the suitable sites identified above and enable us to conduct fieldwork on private lands, we first acquired the contact information of landowners neighboring the Laguna Atascosa NWR, which was provided by the Refuge Manager. We then used the Cameron County property tax appraisal database to look up property boundaries of all landowners loosely overlapping the Laguna Atascosa acquisition boundary and extracted additional landowner contact information, where available, from the appraisal database. Next, we identified candidate sites based on their published patch size in the TEAM system [54] in order to capture a gradient of patch sizes. Where multiple patches with the same habitat class, size range, and estimated land use history were available, we randomly assigned each a priority rank using a random number generator. We then overlaid the private landowner boundary layer and the candidate habitat patch layer and performed spatial queries to see which candidate patches occurred on land with a contactable landowner. In most cases, we had landowner contact information for several finalist sites within the desired habitat type and patch size range, and we contacted landowners according to the site’s assigned priority order to request access. When we lacked contact information for a desired finalist site, we asked the nearest contactable neighbor and/or Laguna Atascosa NWR staff for the missing information, or simply (where possible) knocked on the front door at the property to request access. If these methods failed to lead to landowner contact and ultimately permission, we moved on to the next most similar finalist site until a suitable site with a landowner who granted us research access was found.

The typical plant vegetation structure within our Tamaulipan shrublands class is similar to that described for Tamaulipan mixed deciduous thornscrub habitats, namely a 2–4 m tall thornscrub forest dominated by a canopy of *Prosopis glandulosa* (honey mesquite), *Acacia* species, and other thornscrub tree species such as *Ebenopsis ebano* (Texas ebony) and *Celtis pallida* (spiny hackberry), with a diverse understory of up to 90 species [45]. However, the different subclasses within this class differ in their prevalence of woody species and in the identities of the dominant and subdominant tree and shrub species. Tamaulipan lomas are defined as “windblown sediment deposits that form small, xeric, subtropical, shrubby islands often located in the middle of the salt prairie and tidal flats” [45]. The dominant vegetation in Texas Coastal Prairie generally included *Borrichia frutescens* (sea ox-eye daisy), *Spartina spartinae* (Gulf cordgrass), and other graminoid and forb species [55]. South Texas Disturbed Grasslands consist of both rangelands and old fallow fields, with the later making up all the sites in our study [55]. The soils in the study area are over 90% clay and loam soils with 3% considered sandy [41]. The average annual rainfall in the region is 38 to 76 cm and is very erratic and seasonal, with the highest rainfall in early autumn. Average temperatures in the study area range from 10 °C in winter to 36 °C in summer [41]. The topography of the area is very flat, with elevations of 0–10 m and slopes of less than 1% [56]. 

### 2.2. Lepidoptera Sampling

At each study site, we assessed Lepidoptera diversity and community structure using a linear transect survey method known as the ‘Pollard walk’ [57]. Lepidoptera surveys typically try to maximize coverage of a habitat by utilizing transects that are either lengthy (several km) or numerous [20], but this is often not possible and simplified sampling protocols, such as the rapid assessment methods discussed above, are common. In order to approximate more thorough approaches while also (a) staying within private property boundaries in patches that were often small or irregularly shaped, (b) maximizing sampling effort with a limited workforce (as within a rapid assessment context), and (c) employing systematic and repeatable protocols, we used a gradient of transect lengths for smaller patches (50 m for <2 ha, 100 m for ca. 5 ha, 150 m for ca. 10 ha, and 200 m for ca. 20 ha) and a standard length of 200 m broken into two 100 m transects for patches greater than 20 ha. This cutoff value was based on the abundance of butterfly studies limiting patch sizes to 20 ha or smaller [58]. We included larger patches since recent Lepidoptera studies have found that butterfly species diversity can serve as an indicator of the quality of habitats much larger than traditionally suspected [11]. The paired 100 m transects for patches greater than 20 ha had a minimum spacing of 16 m, which was found to be the minimum distance to observe differences in butterfly composition in the southeastern United States [59]. 

Transects were oriented north to south to run parallel to the assumed primary butterfly migration direction. To position transects, we used QGIS version 2.8 (QGIS Development Team, http://www.qgis.org, accessed on 1 August 2018) geospatial software to select one or two random points within the polygon representing each site (sites over 20 ha had two transects). These random points defined the midpoints of transects. If a random point was close enough to a site boundary that the transect would exceed a habitat or property boundary, a new random point was generated. Transect orientation was modified only if the site shape could not accommodate a north to south transect of the designated length.

During surveys, we used sweep nets to capture all observed Lepidoptera for species identification, unless positive identification was possible without capture. To assist with identification, individual Lepidoptera were placed in an acrylic jar filled with a bed of cotton balls soaked in a solution of 50% water and 50% sports drink (containing both sugar and electrolytes) to reduce the stress of capture [60]. Individuals were either identified to species in the field or photographed for laboratory analysis. A voucher specimen for each Lepidoptera species was collected the first time it was observed, and only from the site at which it was first observed. Vouchers were collected in this manner to minimize the number of specimens collected and to assist in post-survey laboratory identification of species. The initial location and behavior of individuals when they were first observed were also recorded during field surveys. Each transect survey was timed to provide an additional means of quantifying sampling effort. 

Air temperature and wind speed were measured at the start of each survey. Surveys were delayed or canceled if it was actively raining, wind speed exceeded 24 km/h, or the temperature was below 15.5 °C. If precipitation occurred during a survey, this was recorded, but the survey was completed unless it became dangerous to do so. As recommended by a local entomologist, as many transects as possible were surveyed both in the morning (08:00–12:00) and in the afternoon (14:00–18:00) to observe and account for species patterns throughout the day [60]. Laboratory analyses of photographs, voucher specimens, and field notes were performed using reputable field guides [27,28,29,30] to identify Lepidoptera not identified in the field. 

We performed a total of 66 Lepidoptera surveys across 24 study sites (Figure 1). Individual sites were surveyed 1–4 times, with most sites being surveyed twice. Survey effort was roughly equal across habitat classes, except for Tamaulipan lomas, which are rare and for which fewer sites were available. All surveys had the same observer (J.S.) with assistance from 1–2 field technicians and volunteers from a pool of about 10 people. Variation in survey effort was due to unequal accessibility of study sites arising from dynamic road conditions caused by the seasonally heavy rains that occur in early fall and landowner permission. Factors that influenced our access to private lands included the need to acquire permission from all jointly owned property owners (which was as many as 20 individuals for some properties) and postponing surveys to accommodate landowner’s opportunistically scheduled deer hunts. The fall season happens to coincide with deer hunting season in south Texas, which is highly coveted by many landowners for both cultural and economic reasons. Some study sites remained accessible yet could not be resurveyed due to human land use change during the study period; for example, one fallow row crop site was plowed and planted between scheduled surveys. 

### 2.3. Vegetation Sampling

Plant communities were sampled at the same study sites and along the same transects as the Lepidoptera surveys but were performed only once per transect. Plant survey methods were similar to the Gehlhausen et al. [61] protocols for assessing edge herbaceous communities. We quantified percent ground cover by species within a 0.25 m^2^ inner quadrat placed every 10 m along each transect, starting at the transect origin. Within a 4 m^2^ outer quadrat centered on each inner quadrat, we also recorded the presence of any species not found in the inner plot. Woody species in the outer plot were only recorded if their diameter at breast height was at least 2 cm. 

For the same reasons that Lepidoptera sampling effort varied among sites (e.g., road washouts after heavy rain or changes in landowner access permission), not all sites could be surveyed for both Lepidoptera and plant communities before the fall butterfly migration ended in the LRGV. In total, we surveyed Lepidoptera at 23 of our 24 study sites and plants communities at 22 of our 24 sites. We surveyed both plants and Lepidoptera at 21 sites, but nevertheless had site replication and captured a broad gradient of habitat patch sizes. 

### 2.4. Response Variables and Environmental Factors

We normalized all relevant response variables based on sampling effort because of the differences in transect lengths described above. Average percent cover values and Shannon diversity indices did not need normalization because averages are already functions of sampling effort, and Shannon index values derived from at least six samples sufficiently approximate true diversity values in all but the most diverse and/or heterogeneous communities [62,63]. Richness and abundance values were normalized by dividing by either the number of plots (for plants) or transect distance (for Lepidoptera) to produce encounter rates and richness values per unit of sampling effort, respectively. We also calculated Chao 1 estimates of species richness because of the nonlinear nature of species accumulation curves [64], but individual sampling efforts were not always thorough enough for Chao 1 estimates to be reliable. 

For Lepidoptera, we combined the observed values from different surveys of the same site to produce normalized site-level values, which we used in our analyses to avoid pseudoreplication arising from repeated measurements.

As stated above, our original study design attempted to divide sites into two habitat types (woody- or herbaceous-dominated) and two land use history categories (“pristine” or disturbed). However, initial analyses showed that this was an oversimplification, and that nearly all sites exhibited substantial human disturbance (there are essentially no pristine habitats left in the LRGV). Woody plant prevalence and the level of human disturbance were highly variable, even within habitat classes, and distinctions between categories were either subjective or based on limited geospatial data with coarse spatial resolution. Furthermore, broad habitat categories are less useful to land managers seeking to make informed decisions based on specific local conditions. 

Therefore, we analyzed our data using only one categorical predictor (habitat class) and a series of continuous environmental variables. Distinctions between woody- and herbaceous-dominated habitats were readily (and better) achieved by considering woody prevalence as a continuous variable. Human disturbance, however, has many dimensions, and distinctions between more disturbed and less disturbed habitats were more complex. We considered human disturbance using a metric for habitat fragmentation (edge to interior ratio) and, for most analyses, three metrics for invasive, exotic, and pest plant prevalence (explained below). We performed preliminary analyses including patch size as a categorical variable (with two or three levels and different cutoffs between levels) and decided that patch size was best included as a continuous variable. 

Invasive, exotic, and pest plant species are directly and indirectly associated with human disturbance, but in subtly different ways [65]. Plant cover values and encounter rates are not necessarily correlated, and it is reasonable to consider some or all of these categories of plants alone or in combination, thus there are many potential metrics that convey the prevalence of non-native and/or nuisance species. We considered 24 such metrics and selected three that were consistently among those most strongly associated with variation in community composition in preliminary multivariate analyses, and which were not correlated with one another, meaning they could all be included in the same univariate models as well. These three biotic disturbance metrics all used the combined totals of invasive plus exotic plus pest (IEP) plant species and included (1) IEP plant cover, (2) IEP plant encounter rate, and (3) the natural logarithm of the ratio of native plant encounters to IEP plant encounters, which is abbreviated as ‘ln(native:IEP encounters)’ henceforth. 

These metrics were derived from the results of our plant surveys and the native status for observed species listed in the USDA PLANTS database [66]. We defined exotic species as any species not native to Texas, unless we knew a species was native to other regions of Texas but introduced by humans to the LRGV. We defined pest species as those documented as exhibiting weedy traits or posing some sort of nuisance or ecological risk. Inclusion of native pests was important because several native species, such as *Prosopis glandulosa* and *Vachellia fernesiana*, can be very weedy and often pose a nuisance in the habitat types included in this study. Invasive species were defined as those species designated as being both exotic and a pest. 

For Lepidoptera analyses, in addition to the factors above, we considered environmental factors measured during Lepidoptera surveys, namely temperature, wind speed, and rain frequency (the proportion of surveys where rain occurred). We also considered additional plant community metrics derived from our plant surveys hypothesized to influence Lepidoptera communities, specifically plant species richness, plant diversity, total plant cover, blooming species cover, and blooming species encounter rate. We did not quantify the abundance or coverage of blooms during our surveys, but we did record which species were in bloom during the survey period. Thus, our bloom metrics represent the prevalence of species known to have been in bloom at the time, not necessarily the abundance or cover of blooms themselves. 

Lastly, we also considered the abundances of individual Lepidoptera species as response variables but were constrained to analyzing only the 12 most common species due to limited observations. To further address our second and third objectives, we investigated the relationships between the abundances of these Lepidoptera species at each study site and (a) key habitat attributes (habitat class, patch size, edge to interior ratio, IEP plant cover, IEP plant encounter rate, and ln(Native:IEP encounters)); (b) the abundance of potential host plants; (c) blooming plant species encounter rates (i.e., the abundance of likely nectar sources); (d) invasive grass species abundance (hypothesized to be negatively related to both the abundance of host plants, for most species, and nectar sources); and (e) the abundance of plant species with which the Lepidoptera species was observed to be interacting during surveys. All abundance values for individual species were normalized based on sampling effort as before. Potential host plants were quantified by first identifying known host taxa in the literature and then identifying relevant taxa within or sufficiently related to those taxa that were observed in this study. For observed Lepidoptera-plant interactions, we quantified the total combined abundance of plant species that each Lepidoptera species was observed feeding from, foraging on, perched upon, or otherwise interacting with during Lepidoptera surveys.

### 2.5. Statistical Analyses

To characterize the observed wildlife communities and explore the relationships among species and environmental variables, we first performed multivariate analyses of plant, Lepidoptera, and combined communities. We used the ‘metaMDS’ function in the ‘vegan’ package in R version 4.0.5 (R Foundation for Statistical Computing, Vienna, Austria) to fit nonmetric multidimensional scaling (NMDS) ordinations using Bray–Curtis dissimilarity values. We then used the ‘envfit’ function in R to fit the environmental variables listed above as vectors in our ordinations, as well as species vectors for those species most strongly influencing the spread of points. For the combined community analysis, we included the same environmental variables as for Lepidoptera. To visualize these results, we used the ‘ggplot2’ graphing function in R to plot the values generated by ‘metaMDS’ and ‘envfit’.

To test the significance of the effects of habitat class and environmental factors on community composition, we first reduced any sets of correlated environmental variables to a single variable by omitting the variables in a correlated set that explained the least variance. We then used the ‘adonis’ function in R to perform a permutational multiple analysis of covariance (PerMANCOVA) for each community. These PerMANCOVAs used a bootstrapping procedure to generate randomized datasets and compared randomized F statistics to the observed F statistics to calculate *p*-values.

Guided by the results of our multivariate analyses and to further address our second and third objectives, we then performed a series of univariate analyses. First, we used linear and nonlinear regressions to further examine the relationships between our focal environmental variables. We then investigated community level response variables (richness, abundance, and diversity), as above, by first purging the least explanatory of correlated environmental variables. We did so using the ‘step’ function in R to prune relatively complex models (i.e., to remove model terms that explained the least variance) to increase statistical power. Using our pruned models, we examined the effects of the remaining environmental variables by fitting linear models using the ‘lm’ function in R and, in most cases, performing a multifactor Type III analysis of covariance (ANCOVA). If habitat class had a significant effect, we also performed least square means post hoc tests to identify significant differences between individual classes. If the pruned models excluded habitat class, we performed a multiple regression instead of ANCOVA. To confirm these models (and our PerMANCOVAs) met all linear model assumptions, we performed Shapiro–Wilk tests of normality on model residuals, Breusch-Pagan tests for homoscedasticity, and calculated the variance inflation factor for all model terms to quantify multicollinearity using the ‘vif’ function in R. A probability value of *p* < 0.05 was used to determine significance. 

Lastly, we used pruned linear models as described above when analyzing the relationships between the abundance of individual Lepidoptera species and environmental variables. We used simple linear or nonlinear regressions to analyze the relationships between the abundance of individual Lepidoptera species and the abundances of potential host plants, likely nectar sources, invasive grasses, and plants with which the Lepidoptera species were observed to be interacting during this study.

## 3. Results

We observed a total of 160 plant and 112 Lepidopteran morphospecies across all study sites, representing 50 plant families and 16 Lepidoptera families and 38 subfamilies, and from which we were able to positively identify 141 plant and 101 Lepidoptera species (Tables S1 and S2). Of the morphospecies observed, 61% of Lepidoptera and 32% of plants were observed only once. Among the plants, we also observed 10 invasive species (classified as both exotic and pest), 11 exotic species, and 4 pest species. 

The ten most commonly encountered plants were *Borrichia frutescens* (7.9% of plant encounters); *Urochloa maxima* (6.2%, an invasive species); *Parthenium hysterophorus* (4.8%, a pest of uncertain native status); *Monanthochloe littoralis* (4.6%); *Richardia brasiliensis* (4.5%, exotic); *Sorghum bicolor* (4.3%, an introduced grain crop with some weedy behavior); *Pennisetum ciliare* (3.4%, invasive); *Cynodon dactylon* (3.1%, invasive); *Batis maritima* (2.8%); and *Prosopis reptans* (2.8%). Notably, six of the ten most commonly encountered species were invasive, exotic, or a pest. All four of the most common native plants were those prevalent in saline coastal prairies. The 15 most commonly encountered plants in each habitat class are listed in Table 1.

The 11 most frequently observed Lepidoptera were *Libytheana carinenta* (6.7% of observed Lepidoptera), *Pyrisitia lisa* (6.7%), *Danaus gilippus* (4.8%), *Mocis latipes* (4.5%), *Mocis marcida* (4.5%), *Zerene cesonia* (2.6%), *Ascia monuste* (2.2%), *Spoladea recurvalis* (2.2%), *Hemiargus ceraunus* (1.9%), *Hymenia perspectalis* (1.9%), *Panoquina panoquinoides* (1.9%), and *Phyciodes phaon* (1.9%). The 15 most commonly encountered Lepidoptera in each habitat class are listed in Table 2.

Table 3 lists the average values of 26 habitat metrics for the four focal habitat classes surveyed. These values are based on site level metrics quantified for five ‘South Texas Disturbed Grassland’ sites, three ‘Tamaulipan Lomas’ sites, six ‘Tamaulipan Shrublands’ sites, and seven ‘Texas Coastal Prairies’ sites. However, average Lepidoptera abundance, richness, and diversity values for S TX Disturbed Grasslands are based on observations at seven sites, including two where plant community surveys could not be performed and which were excluded from any analyses involving plant metrics.

### 3.1. Multivariate Analyses of Plant and Lepidoptera Communities

#### 3.1.1. Plant Communities

**Figure 2:**
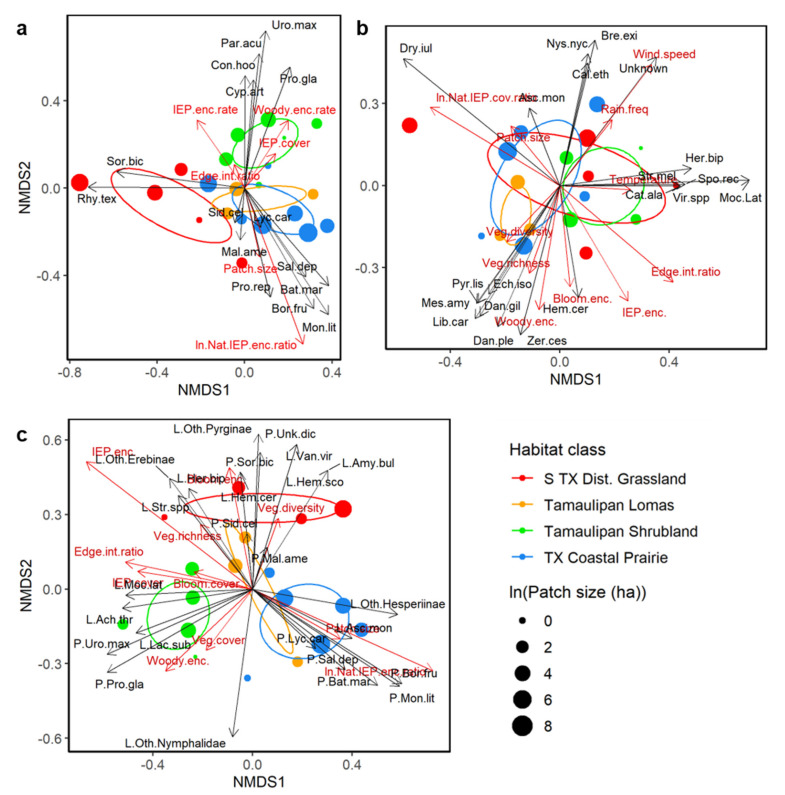
NMDS ordinations representing (**a**) plant, (**b**) Lepidoptera, and (**c**) combined (plant + Lep.) community compositions and similarities among observed communities, which are represented as the position and spatial proximity of points, respectively. Points represent observed communities and correspond to individual study sites. The color and size of points denote habitat class and patch size, as depicted in the inset legend. Black vector arrows denote important species that drove separation among communities in the directions specified based on the observed prevalence of those species. Red vector arrows denote important continuous environmental factors associated with separation among communities in the directions specified. Colored ellipses represent the 95% confidence intervals around the theoretical average communities found in the four focal habitat classes. See Table 4, Table 5, Table 6 (PerMANCOVA results) for additional information related to these ordination. Supplemental Figures S1–S3 depict the same ordinations in a larger, easier to read format. See Tables S1 and S2 for full species names and higher taxonomic information for plants and Lepidoptera, respectively, and for plant species nativity and pest status.

A nonmetric multidimensional scaling (NMDS) ordination of the observed plant communities is shown in Figure 2a. Each point represents one observed plant community and corresponds to a single study site. In all NMDS ordinations, similarity among communities is represented as spatial proximity along the NMDS axes, so the closer together points are, the more similar the communities are that they represent; conversely, the farther points are apart, the less similar are the communities they represent. All ordinations in Figure 2 also include red vectors for key continuous environmental variables and black vectors for species whose abundances most strongly drove the separation of observed communities along the two NMDS axes. Colored ellipses in Figure 2 represent the 95% confidence intervals around the theoretical average communities found in the four habitat classes. Ellipses that do not overlap represent communities considered to be statistically distinct. Supplemental Figure S1 depicts the same ordination as Figure 2a, but in a larger, easier to read format with additional species vectors.

Table 4 shows PerMANCOVA results examining the effects of habitat class and key environmental variables on the positions of observed plant communities in the NMDS ordination shown in Figure 2a. These results indicate that plant communities differed significantly between habitat types and that patch size, woody plant encounter rate, ln(native:IEP encounters), and IEP plant encounter rate were significantly associated with differences in plant community composition. 

Habitat types were separated along both NMDS axes, with S TX Disturbed Grasslands separated from other classes primarily along axis 2 and Tamaulipan Shrublands separated from other classes primarily along axis 1 (Figure 2a and Figure S1). *Sorghum bicolor* (exotic grass) and *Rhynchosia texana* (native vine) most strongly drove separation along NMDS axis 1 and were strongly associated with S TX Disturbed Grassland communities (Figure 2a and Figure S1). *Urochloa maxima* (invasive grass), *Parkinsonia aculeata* (native tree), and *Prosopis glandulosa* (pest tree) most strongly drove separation along NMDS axis 2 and were associated with Tamaulipan Shrubland communities. *Monanthochloe littoralis* (native halophytic grass) and *Batis maritima* and *Borrichia frutescens* (both native halophytic succulents) most strongly drove separation along axes 1 and 2, but in the opposite directions as the prior two species clusters, and were associated with Texas Coastal Prairie communities and, to a lesser extent, Tamaulipan Lomas communities. Tamaulipan Lomas communities overlapped and were positioned within Coastal Prairie plant communities, which reflects the fact that most loma patches are geographically located as islands within a broader matrix of coastal prairie habitats and share many of the same species. 

Patch size and ln(native:IEP encounters) drove separation between communities in the same direction as the cluster of species associated with TX Coastal Prairies listed above, as well as *Salicornia depressa* and *Prosopis reptans* (Figure 2a and Figure S1). However, eigenvectors suggest that ln(native:IEP encounters) had over twice the effect strength of patch size, but both were significant. Edge to interior ratio and IEP plant encounter rate drove separation in approximately the opposite direction as patch size and ln(native:IEP encounters), but edge to interior ratio was not significant in the PerMANCOVA (Table 4). Woody plant encounter rate and IEP plant cover drove separation in a similar direction as those species associated with Tamaulipan Shrublands, as well as *Condalia hookeri* and *Cyperus articulatus*, but IEP plant cover was not significant (Table 4). 

#### 3.1.2. Lepidoptera Communities

Figure 2b and Figure S2 depict an NMDS ordination of observed Lepidoptera communities, with each point representing the community sampled at a single site. Site values include observations from 1–4 surveys performed at a given site that were normalized based on sampling effort. PerMANCOVA results indicate that habitat class, wind speed, edge to interior ratio, and plant diversity significantly influenced Lepidoptera community composition, while temperature had a marginal effect (Table 5). 

Lepidoptera communities were less distinctly separated among habitat classes compared to plant communities and showed substantial overlap among some classes (Figure 2b and Figure S2). As for plants, Lepidoptera communities in Tamaulipan Lomas were positioned within the more variable communities of Texas Coastal Prairies. Separation between Coastal Prairies and Tamaulipan Shrublands was not as distinct as with plants but was still apparent with marginal overlap. Lepidoptera communities of S TX Disturbed Grasslands were extremely variable and broadly overlapped both Coastal Prairies and Tamaulipan Shrubland yet showed considerable separation from and only marginal overlap with Tamaulipan Lomas. 

The Lepidoptera species most strongly driving separation along axis 1 in the positive direction, and thus most strongly associated with Tamaulipan Shrublands, were *Catocala alabamae*, *Herpetogramma bipunctalis*, *Mocis latipes*, *Spoladea recurvalis*, *Strymon melinus*, and *Virbia* spp. (Figure 2b and Figure S2). Most strongly associated with Texas Coastal Prairies and driving separation along both axis 1 (negative) and axis 2 (positive) were *Ascia monuste* and *Dryas iulia*. Those species driving separation most strongly along axis 2 (negative) and weakly along axis 1 (negative) and thus most strongly associated with Tamaulipan Lomas and some Texas Coastal Prairies were *Danaus gilippus*, *Danaus plexippus*, *Echinargus isola*, *Libytheana carinenta*, *Mestra amymone*, *Pyrisitia lisa*, and *Zerene cesonia*. *Hemiargus ceraunus* was near this cluster but drove separation along axis 1 weakly in the positive direction. Most strongly associated with S TX Disturbed Grasslands and some Texas Coastal Prairies and Tamaulipan Shrublands and driving separation strongly along axis 2 (positive) and weakly along axis 1 (positive) were *Brephidium exilis*, *Calpodes ethlius*, *Nyctelius nyctelius*, and a group of unidentifiable Lepidoptera. 

Wind speed drove separation between Lepidoptera communities in the same direction as the cluster of species associated with Disturbed Grasslands and some Coastal Prairies and Shrublands, as well as *Hymenia perspectalis* (an agricultural pest) (Figure 2b and Figure S2). Rain frequency was tightly associated with wind speed but had a lower eigenvalue and was excluded from the final PerMANCOVA model. Edge to interior ratio drove community separation along both axis 1 (positive) and axis 2 (negative) and was thus associated with both Shrublands and Lomas and the species clustering with those habitat classes, but most closely with *Mocis marcida* and *Ringdea cyda*. Even though only edge to interior ratio was significant (Table 5), the eigenvector for IEP plant encounters had a direction and magnitude similar to that of edge to interior ratio, and, as seen for plants, the eigenvectors for ln(native:IEP encounters) and patch size had bearings approximately opposite to that of edge to interior ratio and considerable magnitude. Plant diversity drove separation in the same direction as the cluster of species associated with Tamaulipan Lomas, as well as *Papilio polyxenes*. The plant diversity eigenvector loosely clustered with those of plant richness, woody plant encounter rate, and, to a lesser extent, blooming plant encounter rate, but these factors were not significant (Table 5). Air temperature drove separation along axis 1 (positive) in the same direction as the species cluster associated with Tamaulipan Shrublands and some S TX Disturbed Grasslands. 

Supplementary Figure S3 and Supplementary Table S3 present an alternative ordination of observed Lepidoptera communities and associated PerMANCOVA results, respectively, where each Lepidoptera survey was considered as a standalone observation, rather than using site values that combined observations from multiple surveys. Individual surveys were rather idiosyncratic and highly variable compared to site-level observations and often included only a small number of observations, which together substantially increased variance in community analyses. Nevertheless, results suggest that edge to interior ratio and temperature, which influenced site-level Lepidoptera community composition, as well as ln(Native:IEP encounters) and blooming plant cover significantly influenced survey-level Lepidoptera community composition, while IEP plant cover and whether or not it rained during a survey had marginal effects (Table S3). 

#### 3.1.3. Combined Plant and Lepidoptera Communities

We considered plant and Lepidoptera communities simultaneously primarily to investigate how different plant and Lepidoptera taxa clustered with one another, and we expected Lepidoptera taxa to generally be positioned nearer to higher abundances of plants that serve as food sources for their adults or as host plants for their larvae. 

Figure 2c and Figure S4 show an NMDS ordination of the combined plant and Lepidoptera communities observed in this study, with each point representing a single study site where both plants and Lepidoptera were surveyed. Observations from individual Lepidoptera surveys were pooled by site and normalized based on sampling effort as before. Table 6 shows PerMANOVA results indicating that habitat class, IEP plant encounter rate, ln(Native:IEP encounters), edge to interior ratio, and woody plant encounter rate had significant effects on combined community composition, and that blooming plant cover had a marginal effect. 

Habitat classes separated much as they did for plants previously. Although the positions of groups relative to the NMDS axes differed (which is arbitrary for such ordinations), the relative positioning of habitat class clusters was largely equivalent to that seen for plant communities (Figure 2). S TX Disturbed Grasslands separated from other classes primarily along axis 2, Tamaulipan Shrublands separated along both axes, and Tamaulipan Lomas overlapped with Texas Coastal Prairies. However, unlike before, two of the Lomas communities were separated from the Coastal Prairie cluster (and all others), suggesting that, even if plant and Lepidoptera communities in Lomas overlapped with coastal prairies, they had distinctive species assemblages and/or community structure (Figure 2c and Figure S4). 

Clustering of species and environmental vectors and their associations with particular habitat classes were strongest in the combined community ordination. Tamaulipan Shrublands were characterized by the plants *Urochloa maxima*, *Parkinsonia aculeata*, and *Prosopis glandulosa*, which were clustered with the Lepidoptera *Eantis thraso*, *Lactura subfervens*, and *Mocis latipes*, all of which were associated with higher values for woody plant encounter rate, edge to interior ratio, IEP plant cover, total plant cover, and blooming plant cover (Figure 2c and Figure S4). The plants *Batis maritima*, *Borrichia frutescens*, *Lycium carolinianum*, *Monanthochloe littoralis*, and *Salicornia depressa* clustered with *Ascia monuste*, *Phoebis sennae*, and other Hesperiinae, which were associated with Texas Coastal Prairies (and one Lomas site) and high values for patch size and ln(Native:IEP encounters). S TX Disturbed Grasslands were associated with two discernable clusters. The first, characterized by the plants *Helianthus annuus* and *Richardia brasiliensis* and the Lepidoptera *Herpetogramma bipunctalis*, *Strymon* spp., subfamily Arctiinae, and other Erebinae, exhibited higher plant species richness and a higher IEP plant encounter rate. The second, characterized by the plants *Sorghum bicolor*, *Parthenium hysterophorus*, and *Rhynchosia texana* and the Lepidoptera *Amyna bullula*, *Hemeroplanis scopulepes*, *Hemiargus ceraunus*, *Vanessa virginiensis*, and other Pyrginae, exhibited higher plant diversity and blooming plant encounter rates. The two relatively distinctive Tamaulipan Lomas sites were associated with the plants *Sideroxylon celastrinum* and *Malvastrum americanum*, as well as being relatively weakly associated with the species and environmental variables associated with the second Disturbed Grassland cluster just described. 

### 3.2. Relationships among Key Habitat and Community Attributes

**Figure 3:**
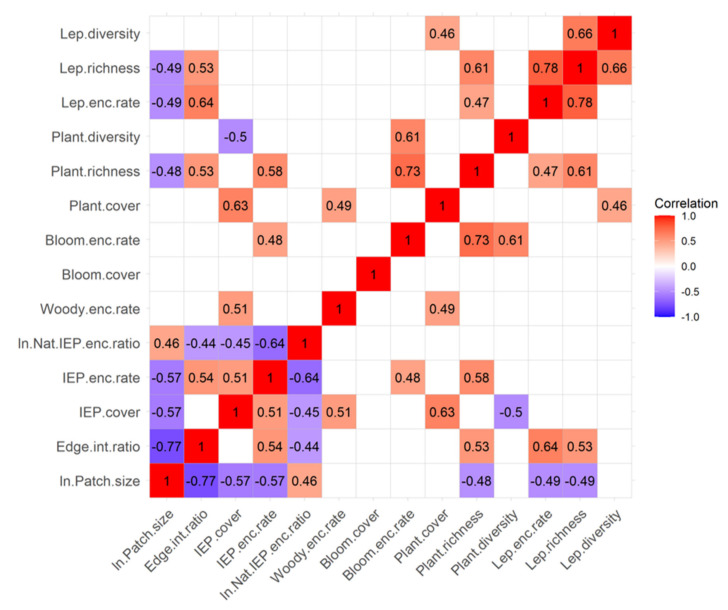
Correlation matrix for 14 key habitat and community attributes. Pearson correlation coefficients are shown for significant correlations (*p* < 0.05), and accompanying cells are colored according to the gradient shown in the inset legend. The 14 variables include, in order from left to right and bottom to top: natural log of patch size (ha), edge to interior ratio, IEP plant cover (%), IEP plant encounters per plot, natural log of the ratio of native to IEP plant encounters per plot, woody plant encounters per plot, blooming species cover (%), blooming species encounters per plot, total plant cover (%), plant richness (species per plot), plant diversity (*H′*), Lepidoptera encounters per 100 m, Lepidoptera richness (species per 100 m), and Lepidoptera diversity (*H′*).

To further address our second and third objectives, we explored and quantified relationships between key habitat and community attributes by performing correlation tests and computing the Pearson correlation coefficient for all possible pairs of 14 focal variables. Figure 3 summarizes these relationships via a colorized correlation matrix that lists the correlation coefficients for significant relationships only (*p* < 0.05). 

Overall, larger habitat patches had lower edge to interior ratios, less IEP plant cover, and fewer IEP plant encounters, which translated into larger patches having a higher ratio of native to IEP plant encounters. However, surprisingly, larger habitat patches also had lower plant and Lepidoptera species richness and lower Lepidoptera abundance (encounter rate). These negative relationships with species richness could simply reflect the fact that our Texas Coastal Prairie sites were larger on average than other habitat classes and, being stressful habitats (high salinity), also had relatively simple communities and a smaller pool of stress-tolerant species. Patch size and edge to interior ratio (EIR) usually have a negative relationship because smaller patches have relatively more edge and less interior area, but irregularly shaped large patches can still have a high EIR, and many of the habitat patches studied had irregular shapes (Figure 1). Negative relationships between patch size and IEP plant cover and IEP plant encounter rate are also expected because larger patches are theoretically harder to invade due to their size, which dampens diffusion of colonizing species. However, how easily new species can move into a habitat depends on its EIR, so the positive relationship between EIR and IEP plant encounter rate is also expected, assuming new species are still dispersing into new areas.

Conversely, habitats with higher edge to interior ratios (EIR) had higher IEP plant encounter rates and lower ratios of native to IEP plant encounters. The positive relationships between EIR and plant richness and Lepidoptera richness and abundance were somewhat unexpected but not surprising because many species of plants and Lepidoptera are edge specialists [53], and because adding IEP species (higher IEP plant encounters) without those IEP species competitively excluding native plants (no relationship to IEP plant cover) should increase richness, especially in highly disturbed landscapes where native plant communities are depauperate.

Higher IEP plant cover was correlated with higher IEP encounter rates, lower native to IEP encounter ratios, higher woody plant encounter rates, higher total plant cover, and lower plant diversity. These first two relationships are logically expected given that they are indicators of greater IEP plant prevalence. The relationship with woody plant cover reflected the fact that the highest IEP plant cover values were observed in the understories of shrublands and savannas, which were often dominated by the invasive grasses, especially *Urochloa maxima*. These same woody understories also tended to have high total plant cover, which may reflect more favorable growing conditions (i.e., higher water availability and lower salinity) that promoted woody plant abundance or were produced by the presence of woody plants (e.g., shading, wind protection, or other nurse plant effects), or both. The negative relationship with plant diversity is also logical because less-competitive species are often displaced when IEP plant cover is high.

Similarly, higher IEP plant encounter rates were correlated with lower native to IEP encounter ratios (which is logical because IEP encounter rate is the denominator), higher blooming plant encounter rates, and higher plant richness. The relationship with blooming plant encounters arose because many of the species in bloom at the time were IEP plants. The presence of more IEP plants (higher encounters rates) without competitive displacement theoretically serves to add species to the local pool and thus increase richness, especially in disturbed habitats with depauperate native plant communities.

Higher woody plant encounter rates were correlated with higher total plant cover for the reasons discussed above related to shrubland and savanna understories. Blooming plant cover was not significantly correlated with any other environmental factors, but blooming plant encounter rates were positively correlated with IEP plant encounter rates (discussed above), plant richness, and plant diversity. The latter two relationships are expected because having more species or higher species diversity increases the likelihood that blooming species would be present.

In addition to the relationships discussed above, higher total plant cover was correlated with higher Lepidoptera diversity, and plant richness was positively correlated with Lepidoptera encounter rates and Lepidoptera richness. All of these positive relationships have often been observed in prior studies [3,8,37,38] and may generally reflect greater resource availability and greater niche diversity for Lepidoptera arising from greater plant abundance and diversity and habitat structural complexity. 

Finally, in addition to the relationships above, higher Lepidoptera encounter rates (abundance) were correlated with higher Lepidoptera richness, and Lepidoptera richness was positively correlated with Lepidoptera diversity. Richness and diversity are mathematically related, but higher abundance does not imply higher richness or diversity, so the prior suggests that observed Lepidoptera communities were relatively diverse and not represented by high abundances of relatively few species. 

### 3.3. Plant Community Univariate Analyses

**Figure 4:**
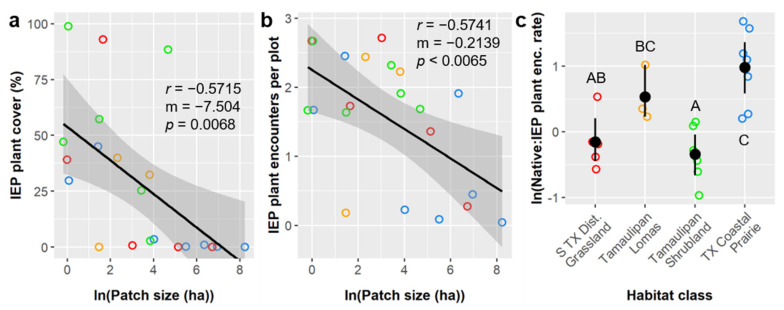
Linear relationships between habitat patch size and (**a**) combined invasive, exotic, and pest (IEP) plant cover and (**b**) IEP plant encounter rate. (**c**) Treatment means with 95% confidence intervals showing the effects of habitat class on the natural log of the ratio of native to IEP plant encounter rates. Open circles denote values from one study site and are colored to denote habitat class as follows: red, S TX Disturbed Grassland; orange, Tamaulipan Lomas; green, Tamaulipan Shrubland; blue, Texas Coastal Prairies. Legend: *r*, Pearson correlation coefficient; m, slope from the regression model (effect size); *p*, *p*-value from the regression model (see Table S4 for *p*-values from the ANCOVA model). Capital letters in panel c denote the results of least square means post hoc tests; groups that share a letter were not significantly different.

We fit a series of linear models and used ANCOVAs and/or regressions to more thoroughly examine how environmental factors influenced key response variables. Despite using them previously as predictors, we first considered our three focal metrics of invasive, exotic, and pest (IEP) plant prevalence as response variables to evaluate the effects of habitat class, patch size, edge to interior ratio, and woody plant prevalence on biotic disturbance. When considered in the same model, none of these habitat attributes had significant marginal effects on IEP plant cover (Type III ANCOVA; *p* > 0.05) or IEP plant encounter rate (Type III ANCOVA; *p* > 0.05). However, pruned models showed that patch size (natural log transformed) had a significant negative effect on both IEP plant cover (regression; m = −7.50, F_1,19_ = 9.22, *p* = 0.0068) and IEP plant encounters per plot (regression; m = −0.214, F_1,19_ = 9.34, *p* = 0.0065) (Figure 4a,b). IEP plant cover and encounter rates varied considerably among habitat classes (Table 3), but this variance was better explained by other factors, and habitat class itself did not significantly influence either. However, habitat class was the only factor that had a significant effect on ln(native:IEP encounters) (Type III ANCOVA; F_6,20_ = 9.95, *p* = 0.0009) (Figure 4c and Table S4). Note, however, that ln(native:IEP encounters) was positively correlated with patch size (Pearson *r* = 0.46) and negatively correlated with edge to interior ratio (*r* = −0.44) when considered alone (Figure 3), and that both of these correlates varied considerably among habitat classes (Table 3).

**Figure 5:**
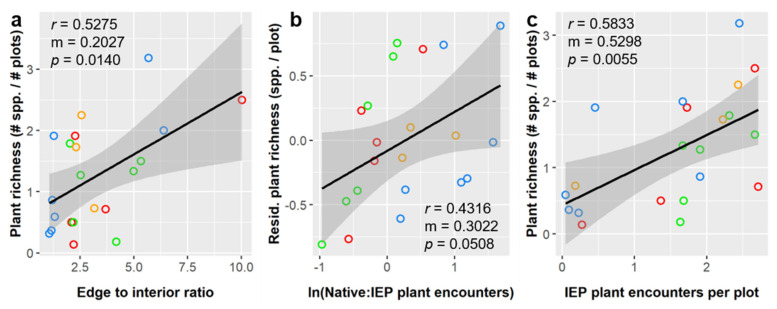
Linear relationships between (**a**) plant richness and edge to interior ratio, (**b**) residual plant richness and the natural log of the ratio of native to IEP plant encounter rates, and (**c**) plant richness and IEP plant encounter rate. Open circles denote values from one study site and are colored to denote habitat class as follows: red, S TX Disturbed Grassland; orange, Tamaulipan Lomas; green, Tamaulipan Shrubland; blue, Texas Coastal Prairies. Legend: *r*, Pearson correlation coefficient; m, slope from the regression model (effect size); *p*, *p*-value from the regression model (see Table S5 for *p*-values from the ANCOVA model).

Plant species richness, normalized as species per plot, averaged 1.25 ± 0.85 spp./plot and was significantly influenced by edge to interior ratio, ln(Native:IEP encounters), and IEP plant encounter rate (Table S5). All three of these factors had a positive linear relationship with plant richness; Figure 5 illustrates these relationships and includes corresponding slope values (effect sizes) and correlation coefficients. 

The relationship between plant richness and ln(Native:IEP encounters) was not significant in our pairwise correlation analyses, but ln(Native:IEP encounters) explained a significant amount of residual variance when considered alongside other factors. This positive relationship suggests that, even though richness was higher in habitats where IEP plant encounter rates were higher, having a higher proportion of native plant encounters relative to IEP plant encounters was also linked to greater plant richness. In other words, richness may be highest in the early stages of invasion, when IEP plant have begun to appear, but before IEP plants begin to overwhelm and displace native species. 

**Figure 6:**
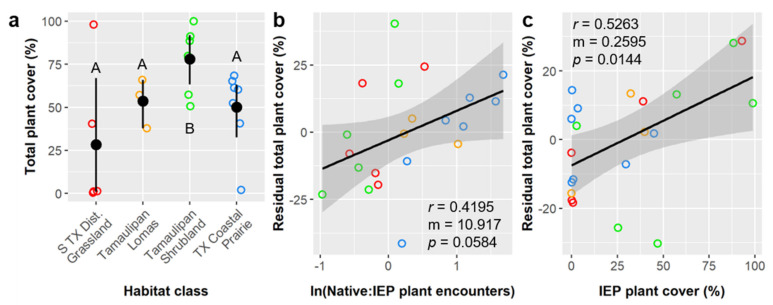
(**a**) Treatment means with 95% confidence intervals showing the effects of habitat class on total plant cover, and the linear relationships between residual total plant cover and (**b**) the natural log of the ratio of native to IEP plant encounter rates and (**c**) IEP plant cover. Open circles denote values from one study site and are colored to denote habitat class as follows: red, S TX Disturbed Grassland; orange, Tamaulipan Lomas; green, Tamaulipan Shrubland; blue, Texas Coastal Prairies. Legend: *r*, Pearson correlation coefficient; m, slope from the regression model (effect size); *p*, *p*-value from the regression model (see Table S6 for *p*-values from the ANCOVA model). Capital letters in panel a denote results of least square means post hoc tests; groups that share a letter were not significantly different.

Plant abundance (total plant cover) averaged 53.3 ± 31.4% overall and was significantly impacted by habitat class, ln(Native:IEP encounters), and IEP plant cover (Table S6). Total plant cover was significantly higher in Tamaulipan Shrublands (77.9%) than in all other habitat classes (Figure 6a). Average plant cover in S TX Disturbed Grasslands (28.3%) was lower than in all other classes but was extremely variable. Residual plant cover had a positive linear relationship with ln(Native:IEP encounters) and IEP plant cover (Figure 6b,c). 

Like with plant richness, ln(Native:IEP encounters) was not significant correlated with plant cover in our pairwise analyses, but it explained significant residual variance in our ANCOVA. Although total cover was higher where IEP plant cover was higher, having a greater proportion of native vs. IEP plant encounters was nevertheless linked to greater total plant cover. This pattern is less likely related to the stage of invasion, as with plant richness, and more likely related to resource (or niche) partitioning, and it suggests that total plant cover (and theoretically net primary productivity) is higher in mixed communities than within invasive monocultures despite interspecific competition. 

**Figure 7:**
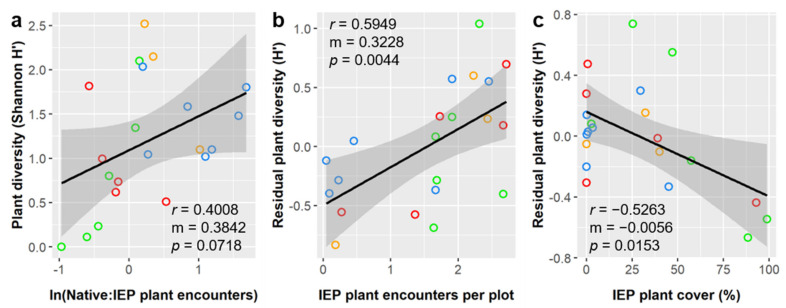
Linear relationships between (**a**) plant diversity and the natural log of the ratio of native to IEP plant encounter rates, (**b**) residual plant diversity and IEP plant encounter rate, and (**c**) residual plant diversity and IEP plant cover. Open circles denote values from one study site and are colored to denote habitat class as follows: red, S TX Disturbed Grassland; orange, Tamaulipan Lomas; green, Tamaulipan Shrubland; blue, Texas Coastal Prairies. Legend: *r*, Pearson correlation coefficient; m, slope from the regression model (effect size); *p*, *p*-value from the regression model (see Table S7 for *p*-values from the ANCOVA model).

Plant diversity (Shannon index, *H’*) averaged 1.20 ± 0.71 and was significantly influenced by ln(Native:IEP encounters), IEP plant encounter rate, and IEP plant cover (Table S7). Plant diversity had a positive linear relationship with ln(Native:IEP encounters) and IEP encounter rate and a negative linear relationship with IEP plant cover (Figure 7).

Like richness, we saw higher plant diversity where the IEP plant encounter rate was higher (Figure 7b), and, like both richness and total cover, diversity was higher where the relative proportion of native vs. IEP plant encounters were higher (Figure 7a). However, unlike total plant cover, plant diversity was lower where IEP plant cover was higher (Figure 7c). A negative relationship between IEP prevalence and diversity is generally expected, and this is consistent with the broader patterns and mechanisms suggested by our previous results. Namely, that lesser degrees of biotic disturbance (e.g., in the early stages of invasion or involving species that are less aggressive or dominant, such as naturalized exotics compared to invasive exotics) generally increased plant community richness, abundance, and diversity. Alternatively, greater degrees of biotic disturbance (e.g., later stages of invasive or involving more dominant invasive or pest species) generally decreased plant diversity (only). Such patterns are consistent with the invasion literature, the intermediate disturbance hypothesis, and a multitude of prior studies. 

### 3.4. Lepidoptera Community Univariate Analyses

**Figure 8:**
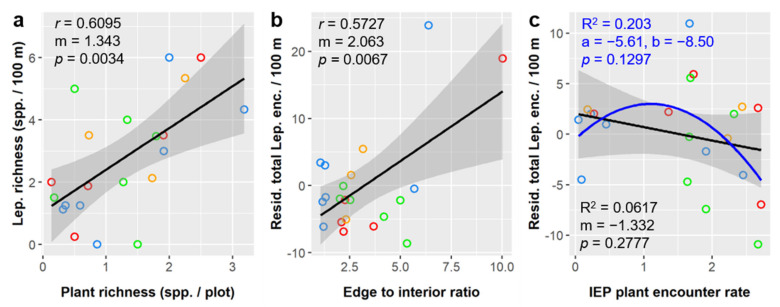
Linear (and nonlinear) relationships between (**a**) Lepidoptera richnness and plant richness, (**b**) residual Lepidoptera abundance (total encounters per 100 m) and edge to interior ratio, and (**c**) residual Lepidoptera abundance and and IEP plant encouter rate. Open circles denote values from one study site and are colored to denote habitat class as follows: red, S TX Disturbed Grassland; orange, Tamaulipan Lomas; green, Tamaulipan Shrubland; blue, Texas Coastal Prairies. The blue curve in panel c is the second-order polynomial best-fit line. Legend: *r*, Pearson correlation coefficient; m, slope from the regression model (effect size); *p*, *p*-value from the regression models shown (see main text and Table 7 for *p*-values from the multiple regression models); R^2^ = coefficient of determination (included to compare the linear and polynomial models); a and b, polynomial equation coefficients.

Lepidoptera richness averaged 2.74 ± 1.88 species per 100 m of survey distance, and, despite the correlations described above, was only significantly influenced by plant richness when different environmental factors were considered together (pruned multiple regression; F_2,20_ = 4.75, *p* = 0.0429). Lepidoptera richness had a positive linear relationship with plant richness (Figure 8a). 

Lepidoptera abundance averaged 6.73 ± 8.32 total individuals encountered per 100 m of survey distance and was significantly influenced by edge to interior ratio, wind speed, IEP plant encounter, temperature, and plant diversity (Table 7). Lepidoptera encounters per 100 m had a positive linear relationship with edge to interior ratio (Figure 8b), temperature (Pearson *r* = 0.3306, m = 0.6456), and plant diversity (*r* = 0.2691, m = 1.903), and a negative linear relationship with wind speed (r = −0.3579, m = −0.4263), and IEP plant encounter rate (Figure 8c). Although the linear relationship with was significant in our multiple regression model, the relationship between residual lepidoptera abundance and IEP plant encounter rate was better described using a second-order polynomial fit (Figure 8c). 

**Figure 9:**
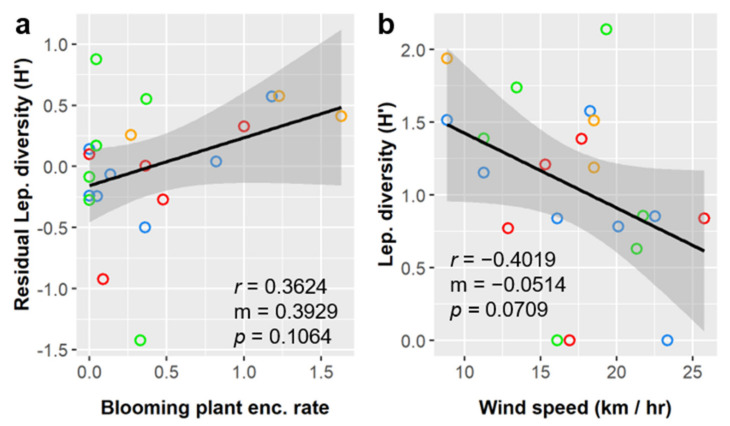
Linear relationships between (**a**) residual Lepidoptera diversity and blooming plant encounter rate and (**b**) Lepidoptera diversity and wind speed. Open circles denote values from one study site and are colored to denote habitat class as follows: red, S TX Disturbed Grassland; orange, Tamaulipan Lomas; green, Tamaulipan Shrubland; blue, Texas Coastal Prairies. Legend: *r*, Pearson correlation coefficient; m, slope from the regression model (effect size); *p*, *p*-value from the simple regression model shown (see Table 8 for *p*-values from the multiple regression model).

Lepidoptera diversity (Shannon index, *H’*) averaged 1.06 ± 0.60 across all sampled sites and was significantly influenced by blooming species encounter rate and was marginally influenced by wind speed (Table 8). Lepidoptera diversity had a positive linear relationship with blooming plant species encounter rate and a negative linear relationship with wind speed (Figure 9). Lepidoptera diversity varied considerably among habitat classes (Table 3) and was highest in Tamaulipan Lomas (1.55) and lowest in S TX Disturbed Grasslands (0.75), but these differences were not significant and were better explained by continuous environmental variables. Some relationships between Lepidoptera diversity and environmental variables were not linear, for example, diversity had a second-order polynomial relationship with patch size (not shown), but these relationships were not statistically significant.

### 3.5. Resource-Based Relationships between Plants and Lepidoptera

Table 9 summarizes the relationships between the abundances of the 12 most common Lepidoptera species and (a) key environmental variables and the abundances of (b) potential host plant species, (c) likely nectar sources, and (d) plant species with which the Lepidoptera species were observed to have interacted. Relationships with the abundance of invasive grasses were also analyzed, but none were significant. The strongest relationship with invasive grasses was exhibited by *Phyciodes phaon* (*r* = 0.33, *p* = 0.1455). 

For five of the 12 most common Lepidoptera species, their abundance was significantly correlated to at least one habitat attribute, but those attributes varied. Two were influenced by edge to interior ratio, two by IEP plant cover, one by habitat class, one by patch size, and one by IEP plant encounter rate. Notably, four of these five species are year-round residents of the LRGV. 

Lepidoptera abundance was significantly correlated with host plant abundance for only one resident Lepidoptera species (*Hemiargus ceraunus*) and one migratory species (*Pyrisitia lisa*). Similarly, the abundance of likely nectar sources (quantified as the blooming plant species encounter rate, which, as discussed above, does not necessarily represent bloom abundance) was correlated with only one resident (*Phyciodes phaon*) and one migratory Lepidoptera species (*Zerene cesonia*). Lastly, the abundance of plant species with which a Lepidoptera species was observed to have interacted during our surveys was also significantly correlated with only one resident (again *Hemiargus ceraunus*) and one migrant Lepidoptera species (*Ascia monuste*). Notably, when Lepidoptera abundance was correlated with the abundance of plant species that it had interacted with during surveys, those plant species differed from the potential host plants but included at least one likely nectar plant. 

We had three general resource-based hypotheses about the abundances of individual Lepidoptera species. First, the same environmental variables that influence community structure and composition will influence individual Lepidoptera species abundance. There was some evidence to support this. The abundances of more species were correlated with a habitat attribute or metric of biotic disturbance than with a particular plant-based resource, but fewer than half showed such a relationship. 

Second, the abundance of individual Lepidoptera species within a habitat patch should be influenced by the abundance of plant species to which that Lepidoptera is ecologically linked, particularly as host plants for larvae or as food (typically nectar) sources for adults. Support for this hypothesis was limited. We saw few significant correlations (two each) between Lepidoptera abundance and host plants or likely nectar sources. Support for this and the prior hypothesis was almost certainly impacted by a scarcity of observations. Although we sampled both plants and Lepidoptera at 21 study sites and most sites were surveyed for Lepidoptera 2 or more times, observations of individual Lepidoptera were still very limited; seven of the twelve most common Lepidoptera were observed fewer than ten times.

Third, we hypothesized that non-resident Lepidoptera species present in the LRGV only as migrants were more likely to be influenced by the abundance of nectar sources than by host plant abundance. By chance, half of the most common Lepidoptera species (6 of 12) are migratory in the LRGV, and the other half are year-round residents. We again saw only two significant correlations each between Lepidoptera abundance and the abundance of host plants or nectar sources, and, in both cases, they were evenly split between resident and migratory species.

## 4. Discussion

As expected, habitat class and our focal environmental variables were associated with significant differences among sites in the plant and/or Lepidoptera communities observed (Table 1, Table 2, Table 3, Figure 2). However, which factors influenced a particular metric varied broadly, and some key response variables such as Lepidoptera diversity were influenced by surprisingly few factors (Table 8). Most of our findings agreed with prior studies; however, some do not, and the effects of patch size, fragmentation, and biotic disturbance were not always what we expected. Thus, one of the core findings of this study agrees with one of the broader conclusions emerging from current research on the biodiversity of Lepidoptera and other taxa across human-impacted landscapes, namely: it is complicated. That is, here and in related studies, multiple drivers are implicated, and these drivers may interact additively, synergistically, or antagonistically, and they operate across different patterns of space and time [37,53,67,68]. This does not mean, however, that patterns of biodiversity and ecological condition are intractably context specific. Rather, there are broad patterns floating in a soup of nuances and complexities. 

### 4.1. Importance of Human Alterations and Biotic Disturbance

The first, and perhaps most important, conclusion we can draw from our findings is that the private lands in the LRGV of south Texas are overwhelmingly highly disturbed. Human alteration and biotic disturbance (IEP plant prevalence) are widespread and often intense in this conservation hotspot. Habitats classified as Tamaulipan Thornscrub (part of our Tamaulipan Shrubland class) are broadly seen as premier habitats of high conservation value, but, while this may be true, even they suffer from major anthropogenic disturbance. Invasive, exotic, or pest plant cover in our Shrubland class averaged 53.3%, we encountered nearly two (1.98) IEP plant species per plot, and most sites had more IEP plant cover or encounters than native plant cover or encounters (leading to negative log ratio values) (Table 3). Biotic disturbance was also high in Lomas and Disturbed Grasslands, and relatively low only in stressful saline Coastal Prairies, where native halophytes remained dominant. 

Furthermore, larger patches and those with lower edge to interior ratios provided relatively little buffer to this biotic invasion or its effects. We saw expected negative relationships between patch size and IEP plant cover and encounter rates (Figure 4a,b), but this did not translate into benefits to richness or diversity. Plant and Lepidoptera richness and Lepidoptera abundance were negatively correlated with patch size (Figure 3), though this pattern was at least partly driven by the fact that saline Coastal Prairie sites had relatively large sizes and simple communities with fewer IEP plants (discussed above). Importantly, plant and Lepidoptera diversity were unrelated to patch size (Figure 3). We also saw positive relationships between edge to interior ratio and both IEP encounter rate and plant richness because the latter two were positively correlated themselves (Figure 3 and Figure 5c). 

However, the effects of greater biotic disturbance were surprisingly weakly negative or indistinct—and sometimes even positive (in the context of the relationships tested)—in this study. Higher IEP plant encounters were significantly linked to higher plant richness (Figure 5c) and higher plant diversity (Figure 7b), and neither IEP plant cover nor encounters significantly influenced Lepidoptera richness or diversity. Higher Lepidoptera richness was indirectly linked to higher IEP plant encounters because plant and Lepidoptera richness were positively related (Figure 8a), and so too were plant richness and IEP plant encounters (Figure 5c). These patterns have important implications related to our first conclusion: that human disturbance is extensive and strong in the LRGV. 

Crucially, despite our best efforts to sample across a gradient of human disturbance, our observations did not include an adequate set of habitats with very low biotic disturbance that could allow us to characterize relatively pristine habitats and provide a robust frame of reference for the more biotically disturbed habitats. In other words, habitats with minimal biotic disturbance were exceedingly rare, so we do not have a good picture of what undisturbed habitats, or the communities therein, look like—if they even exist on private land in the region. This is partly why we saw weak negative and sometimes positive effects of biotic invasion on community metrics. 

More practically, however, this means (biotically) disturbed landscapes are overwhelmingly the norm in south Texas (except perhaps for more stressful saline coastal prairies and long-protected public lands), and our observations basically documented habitats that varied more in terms of their stage or intensity of invasion or human alteration. Therefore, the weakly negative or positive impacts associated with greater biotic disturbance and fragmentation may be a strong indication that invaded and/or disturbed habitats are simply the new normal for the LRGV. That is, perhaps anthropogenic alterations and biotic disturbance are so pervasive and widespread that novel ecosystems with species assemblages rich in (if not dominated by) invasive, exotic, and pest species are now typical and characteristic of south Texas (see Table 1), as is true for many other parts of the world [69,70]. 

### 4.2. Habitat Class

One of our main hypotheses about habitat types, and broadly supported by the literature, was that the distinctiveness of plant communities between habitat types (and particularly woody- vs. herbaceous-dominated habitats) would drive differences in Lepidoptera communities between habitat types as well [4,10,11,71,72]. This was not the case if we oversimplified habitat categories (not shown) or considered individual Lepidoptera surveys, which were often idiosyncratic and/or contained few observations, in our analyses (Figure S3). However, both plant and associated Lepidoptera communities were distinctly different among habitat types when we considered an appropriate set of habitat classes and used site-level Lepidoptera data (Table 4, Table 5, Table 6 and Figure 2, Figures S1, S2, and S4). 

As expected, plant and Lepidoptera communities differed significantly among habitat classes, but distinctions between Tamaulipan Lomas and Texas Coastal Prairies were much weaker than anticipated, and Lepidoptera communities in S TX Disturbed Grasslands overlapped most others (Figure 2). Lepidoptera communities found in Lomas overlapped and appeared as a distinct subset of those found in Coastal Prairies, but this makes sense because Lomas exist geographically as islands in a matrix of Coastal Prairie and share a lot of the same species despite having distinctive features, such as more woody plants (which agrees with the woody encounter vector, for example). Although not as strongly as for plant communities, we see distinct separation between Lepidoptera communities of Shrublands compared to the Coastal Prairie-Lomas complex. This is also logical because these groups represent significantly different plant communities with less species overlap and different vegetative structure. For Lepidoptera, Disturbed Grassland communities overlapped with almost all others, but even this has a straightforward likely explanation: the plants prevalent in Disturbed Grasslands are overwhelmingly weeds and invasive species present almost everywhere else, and the Lepidoptera that occur there are either those associated with the weedy or invasive species, or they are the cosmopolitan species that are habitat generalists and occur almost everywhere. If invasive, exotic, and pest plants were not so highly prevalent in the other habitat classes, the Lepidoptera communities of Disturbed Grasslands would probably cluster away from the rest; however, since all the habitat classes had considerable biotic disturbance, those Lepidoptera communities overlap with all other classes instead. 

Distinctions between habitat types can be blurred by the homogenizing effects of human disturbance [8,11,65,69], and we observed formidable levels of disturbance in this study, but both plant and (to a lesser extent) Lepidoptera communities remained distinct. That said, the cover, encounter rate, and proportional representation of IEP plant species were powerful drivers of separation between observed plant and Lepidoptera communities in our ordinations (Figure 2). We did not, however, see differences between habitat types in overall Lepidoptera richness, abundance, or diversity, nor in overall plant richness or diversity, but none of these metrics need necessarily vary, even if community composition is widely different. This was somewhat surprisingly, though, and could be related to the aforementioned homogenizing effects of disturbance. 

### 4.3. Patch Size and Fragmentation

Our core hypothesis about human landscapes alteration was that habitat loss and fragmentation (smaller patches and higher edge to interior ratios) decrease plant community diversity and/or complexity and thereby drive decreases in Lepidoptera abundance and/or diversity. This process is broadly supported by the literature [3,4,8,37,38] and has logical bases in species area relationships [52] and spatial patterns of habitat heterogeneity [38]; however, it was only weakly supported by our observations. Patch size was a significant environmental variable in our multivariate plant community analysis, but edge to interior ratio was not (Table 4), and neither factor significantly influenced Lepidoptera or combined community compositions (Table 5 and Table 6). It is possible that the mobility of Lepidoptera decoupled their diversity patterns from patch size, given that there is a matrix of available habitats within a reasonable travel distance in the study region [73]. More to the contrary, smaller patch sizes and higher edge to interior ratios were associated with increased plant and Lepidoptera richness and Lepidoptera abundance (Figure 3, Figure 5a, Figure 8b and Table 7, Table S5). 

There were clear links between plant and Lepidoptera communities, though, as seen with community composition (Figure 2); correlations between plant cover and Lepidoptera diversity and between plant richness and Lepidoptera richness and abundance (Figure 3); positive linear relationships between plant and Lepidoptera richness (Figure 8a) and blooming plant encounter rates and Lepidoptera diversity (Figure 9a); and the significant relationships summarized in Table 9. Thus, the disconnect between our observations and our expectations regarding the effects of patch size and fragmentation on wildlife communities had more to do with the nature of these effects than on the links between plants and Lepidoptera.

We expect this disconnect is rooted partly in having relatively large and simple Coastal Prairie habitats (discussed above) but more so in our first major conclusion, namely that human disturbance in the region is so intense and extensive that nearly all private lands are at a relatively early successional stage, and the regional species pool (re)colonizing recently human-modified habitats is now largely characterized by invasive, exotic, and pest species. As a result, patches that are smaller and/or have higher edge to interior ratios have experienced more colonization by native and exotic early-successional plant species in the limited amount of time since their last disturbance. Such an interpretation is consistent with our observations and all those results discussed in this section.

### 4.4. Biotic Disturbance

Also consistent with the prior interpretation are the prevalence of IEP plant species in our study sites (Table 1 and Table 3), the importance of IEP plant species to community composition (Figure 2, Figures S1–S4 and Table 4, Table 5, Table 6), and the abundance of significant effects that IEP plants had throughout our univariate analyses. It appears that biotic disturbance has played a greater role in influencing observed plant and Lepidoptera communities than any other factors we were able to quantify. 

We have previously referred to the intermediate disturbance hypothesis and its suggestions that plant and Lepidoptera diversity and/or abundance could peak in disturbed habitats if they exhibit moderate levels of disturbance and thereby possess a mix of both early and late successional species [3,8], and/or edge and interior specialist species [53], and/or native and exotic species [39,40]. We cannot conclusively test or demonstrate this because, as discussed above, disturbance is so widespread that we lack a good frame of reference for undisturbed habitats in the region. However, several of our findings are consistent with this hypothesis that may, at face value, appear to conflict. Namely, that plant richness, abundance, and diversity all increased with IEP plant cover or encounter rates (Figure 5c, Figure 6c, and Figure 7b, respectively) but also increased when a larger proportion of plant encounters were with natives than with IEP species (Figure 5b, Figure 6b, and Figure 7a, respectively). Furthermore, Lepidoptera abundance follows the characteristic hump-shaped pattern with IEP plant encounters predicted by the intermediate disturbance hypothesis (Figure 8c). These findings largely agree with findings from prior studies [23,39,40]. 

### 4.5. Future Directions

This study had several important limitations due to its limited temporal scope. Any study that samples wildlife, especially highly mobile and variable taxa such as Lepidoptera, during only one year or season is inherently limited in its capacity to fully characterize focal communities. We cannot illustrate or provide any indication of interannual or inter-seasonal variability in plant or Lepidoptera communities, nor can we identify if any observed species are subject to boom-and-bust cycles, and if so, where in those cycles they were at the time of study. Despite our many findings, these gaps mean that the dataset reported here represents a relatively limited baseline. Thus, one important future direction should be simply to expand the sampling approaches used here to include additional years and seasons. 

More generally, additional studies of plant and Lepidoptera communities in private lands are merited to better understand the nature and extent of habitat disturbance and its effects on regional biodiversity, and to get a fuller picture of Lepidoptera ecology in the region. Future studies in private lands should be paired with surveys in protected lands and include habitats that are in a closer to pristine ecological condition. This way, direct comparisons can be made between more impacted ecosystems and more pristine ecosystems with less uncertainty about the extent of human disturbance. This is both important and a major challenge given the long history of landscape alteration in the region (discussed above) [46], especially compared to the relatively recent beginning of efforts (ca. 1982) to preserve and restore biodiversity and important habitats and to establish multicounty wildlife corridors across the LRGV landscape. 

Future studies should also explore the differences between resident Lepidoptera communities versus seasonal communities (e.g., those present during migrations) in the LRGV. If these distinctions are better understood, we would be better able to develop reliable short-duration rapid assessment methods that land managers could use to assess biodiversity and ecological conditions at any time of the year. Additionally, there is a need to better understand the specific behavioral linkages between vegetation communities (particularly host plants and nectar source plants) and the richness, abundance, diversity, and behavior of Lepidoptera at the individual, population, and community levels. Similarly, investigating the linkages between Lepidoptera and plant phenology and flowering behavior at different spatial scales, *sensu* Cariveau et al. [44], would provide a more mechanistic understanding of these behavioral linkages. Thus, greater and more detailed quantification of the abundance, diversity, and spatiotemporal patterns of blooms (actively flowering plants) is merited in the future. Bloom data are important, especially for Lepidoptera, but was very limited in this study. Understanding these relationships could be very important to the conservation of rare or otherwise imperiled plant and Lepidoptera species.

The broad and deep scope of human disturbance and widespread prevalence of invasive, exotic, and pest species documented in this study serve to reaffirm the high value of the few remaining intact and largely non-invaded plant communities in the LRGV, as well as the value of the protected lands that house these plant communities. These findings also illustrate the great parallel needs to conserve relict native plant communities, to manage invasive species, and to actively restore native plant habitats in the region, such as salt prairies and Tamaulipan thornscrub forests. 

## 5. Conclusions

The private lands we surveyed in the LRGV of south Texas were overwhelmingly highly disturbed. Human alteration and biotic disturbance (IEP plant prevalence) were widespread and often intense in this conservation hotspot. Larger patches and those with lower edge to interior ratios had lower IEP plant prevalence, but biotic disturbance was still considerable. However, unexpectedly, the effects of greater biotic disturbance were weakly negative, indistinct, or even positive. For example, there we significant positive relationships between IEP plant encounters and both plant richness (Figure 5c) and plant diversity (Figure 7b), and neither IEP plant cover nor encounters significantly influenced Lepidoptera richness or diversity. This may be a strong indication that invaded and/or disturbed habitats are simply the new normal for the LRGV, and that anthropogenic alterations and biotic disturbance are so pervasive and widespread that novel ecosystems with species assemblages characterized by invasive, exotic, and pest species are now typical of south Texas (see Table 1). Biotic disturbance played a greater role in influencing observed plant and Lepidoptera communities than any other factor we were able to quantify.

Nevertheless, both plant and Lepidoptera communities were distinctly different among habitat types. Distinctions between Tamaulipan Lomas and Texas Coastal Prairies were weaker than anticipated, and Lepidoptera communities in S TX Disturbed Grasslands overlapped communities of most other habitat types. The latter is likely because the plants prevalent in Disturbed Grasslands are overwhelmingly weeds and invasive species present almost everywhere else, and the Lepidoptera that occur there are either species associated with weedy or invasive plants, or they are cosmopolitan habitat generalist species and occur almost everywhere. 

Patch size was a significant environmental variable in our multivariate plant community analysis, but edge to interior ratio was not (Table 4), and neither factor significantly influenced Lepidoptera or combined community compositions (Table 5 and Table 6). The mobility of Lepidoptera may have decoupled their diversity patterns from patch size since there is a matrix of available habitats within a reasonable travel distance in the study region [73]. Surprisingly, smaller patch sizes and higher edge to interior ratios were associated with greater plant and Lepidoptera richness and Lepidoptera abundance, but this makes sense in a context of widespread disturbance where most habitats are essentially recovering and in the early stages of succession. 

## Figures and Tables

**Table 1:** Lists of the 15 most abundance plant species observed within the four focal habitat classes in the Lower Rio Grande Valley of south Texas, ordered by prevalence. Status indicates whether a species is a non-native exotic (E), considered a pest (P), or both exotic and a pest (an invasive species; I); blank values indicate a species is native to the region and not considered a pest. Survey effort varied among habitat classes, so prevalence was normalized and quantified as the proportion of plots where a plant species was encountered (# plots where encountered/total plots). Full species names and taxonomic information corresponding to the species codes shown here are found in Table S1 (the full plant species list). Legend: enc., encounters; spp., species.

Rank	S TX Disturb. Grassland			Tamaulipan Lomas			Tamaulipan Shrubland			TX Coastal Prairie		
	Species	Status	Enc./Plot	Species	Status	Enc./Plot	Species	Status	Enc./Plot	Species	Status	Enc./Plot
1	*Sor.bic*	E	0.63	*Par.hys*	P	0.57	*Uro.max*	I	0.79	*Bor.fru*		0.73
2	*Rhy.lat*		0.21	*Ric.bra*	E	0.35	*Pro.gla*	P	0.30	*Mon.lit*		0.46
3	*Cyn.dac*	I	0.20	*Pen.cil*	I	0.33	*Ric.bra*	E	0.27	*Bat.mar*		0.26
4	*Par.hys*	P	0.20	*Bor.fru*		0.24	*Oxa.str*		0.24	*Sal.dep*		0.22
5	*Unk.dic*		0.15	*Cyn.dac*	I	0.20	*Cyn.dac*	I	0.12	*Pro.rep*		0.19
6	*Ric.bra*	E	0.11	*Teu.cub*		0.20	*Cyp.art*		0.10	*Sua.lin*		0.18
7	*Ipo.hed*	E	0.10	*Cel.pal*		0.18	*Aca.tet*		0.08	*Cyn.bar*		0.17
8	*Sid.acu*		0.09	*Uro.max*	I	0.18	*Bot.isc*	I	0.08	*Pen.cil*	I	0.16
9	*Cyc.lep*	E	0.07	*Opu.eng*		0.18	*Cyc.lep*	E	0.08	*Par.hys*	P	0.15
10	*Cyp.era*		0.07	*Pro.rep*		0.16	*Pen.cil*	I	0.08	*Spa.spa*		0.13
11	*Dig.san*	I	0.07	*Bat.mar*		0.10	*Ana.arv*	E	0.07	Unknown		0.13
12	Unknown		0.06	*Cha.hum*		0.10	*Par.acu*		0.07	*Eup.mac*		0.11
13	*Cor.*spp.		0.05	*Gla.bip*		0.10	*Con.hoo*		0.06	*Nep.pub*		0.11
14	*Pen.cil*	I	0.05	*Lan.urt*		0.10	*Lan.urt*		0.06	*Ric.bra*	E	0.10
15	*Pro.rep*		0.05	*Leu.fru*		0.10	*Tar.off*	I	0.06	*Men.het*		0.09
	Others		0.62	Others		1.86	Others		0.99	Others		1.54
	All plants		2.72	All plants		4.98	All plants		3.45	All plants		4.72

**Table 2:** Lists of the 15 most abundance Lepidoptera species observed within the four focal habitat classes in the Lower Rio Grande Valley of south Texas, ordered by abundance. Survey effort varied among habitat classes, so abundance was normalized and quantified as the number of individuals of a given species encountered per 100 m of survey distance. Full species names and taxonomic information corresponding to the species codes shown here are found in Table S2 (the full Lepidoptera species list). Legend: enc., encounters; spp., species; Leps., Lepidopterans.

Rank	S TX Disturb. Grassland		Tamaulipan Lomas		Tamaulipan Shrubland		TX Coastal Prairie	
	Species	Enc./100 m	Species	Enc./100 m	Species	Enc./100 m	Species	Enc./100 m
1	*Spo.rec*	0.61	*Lib.car*	1.54	*Lib.car*	0.44	*Asc.mon*	1.19
2	*Hem.cer*	0.52	*Pyr.lis*	0.85	*Moc.lat*	0.39	*Lib.car*	0.94
3	Unknown	0.17	*Zer.ces*	0.54	*Pyr.lis*	0.29	*Dan.gil*	0.47
4	*Moc.lat*	0.14	*Dan.gil*	0.46	*Moc.mar*	0.24	*Pyr.lis*	0.47
5	*Moc.mar*	0.14	*Dan.ple*	0.15	Unknown	0.24	*Pan.pan*	0.22
6	*Dan.gil*	0.12	*Hem.cer*	0.15	*Ana.jat*	0.20	Unknown	0.19
7	*Pyr.lis*	0.12	*Kri.lys*	0.15	*Hym.per*	0.20	*Zer.ces*	0.13
8	*Lib.car*	0.12	*Pap.pol*	0.15	*Pan.pan*	0.20	*Eup.ves*	0.09
9	*Rin.cyd*	0.12	*Pho.sen*	0.15	*Ach.thr*	0.15	*Moc.lat*	0.09
10	*Bag.rep*	0.09	*Pyr.alb*	0.15	*Ela.fus*	0.15	*Cal.eth*	0.06
11	*Her.bip*	0.09	*Agr.van*	0.08	*Ere.*spp.	0.15	*Cen.pet*	0.06
12	*Amy.bul*	0.06	*Ana.jat*	0.08	*Rin.cyd*	0.15	*Cym.odi*	0.06
13	*Hel.lav*	0.06	*Ani.ill*	0.08	*Mel.ind*	0.10	*Ech.iso*	0.06
14	*Hem.iso*	0.06	*Ani.sim*	0.08	*Phy.pha*	0.10	*Hel.lav*	0.06
15	*Hem.sco*	0.06	*Cis.plu*	0.08	*Zer.ces*	0.10	*Moc.dis*	0.06
	Other spp.	1.10	Other spp.	1.15	Other spp.	1.61	Other spp.	0.66
	All Leps.	3.57	All Leps.	5.85	All Leps.	4.68	All Leps.	4.81

**Table 3:** Average values of 26 habitat metrics for the four focal habitat classes surveyed. Bloom abundance was not quantified for individual plots or sites, but species in bloom during surveys were recorded; thus, bloom metrics represent the prevalence of species known to have been in bloom at the time, not necessarily the abundance or cover of blooms themselves.

Habitat Metric	S TX Disturbed Grasslands	Tamaulipan Lomas	Tamaulipan Shrublands	TX Coastal Prairies
Patch size (ha)	206.4	20.1	31.9	812.2
ln(Patch size (ha))	3.31	2.54	2.22	4.65
Edge:Interior ratio (km/ha × 100)	4.06	2.68	3.54	2.59
Total plant cover (%)	28.3	53.5	77.9	50.0
Plant richness (spp./site)	13.0	27.3	15.3	19.4
Plant richness (spp./plot)	1.15	1.57	1.10	1.32
Plant diversity (*H′*)	0.94	1.92	0.77	1.44
Lep. abundance (encounters/100m)	3.57	5.85	4.68	4.81
Lep. richness (spp./site)	10.1	13.3	10.2	7.9
Lep. richness (spp./100 m)	2.31	3.65	2.66	2.42
Lep. Diversity (*H′*)	0.75	1.55	1.13	0.96
Woody plant encounters/plot	0.04	0.74	0.70	0.35
Blooming plant cover (%)	0.3	10.5	9.0	0.6
Blooming plant encounters/plot	0.39	1.04	0.13	0.36
Invasive (I) plant cover (%)	26.1	14.8	51.0	10.6
Exotic (E) plant cover (%)	0.1	1.9	2.0	0.1
Pest (P) plant cover (%)	0.3	7.5	0.3	0.7
I+E+P plant cover (%)	26.6	24.1	53.3	11.3
Invasive plant encounters/plot	0.69	0.64	1.14	0.47
Exotic plant encounters/plot	0.78	0.41	0.36	0.21
Pest plant encounters/plot	0.28	0.56	0.48	0.29
I+E+P plant encounters/plot	1.75	1.62	1.98	0.98
Native:I+E+P plant cover ratio	0.97	13.96	5.19	27.82
ln(Native:I+E+P plant cover ratio)	−0.94	0.99	−1.58	1.83
Native:I+E+P plant enc./plot ratio	1.09	2.50	1.10	3.68
ln(Native:I+E+P plant enc./plot ratio)	−0.15	0.53	−0.34	0.98

**Table 4:** Permutational multiple analysis of covariance (PerMANCOVA) results examining the effects of habitat type, patch size, edge to interior ratio, woody plant encounter rate, ln(Native:IEP encounters), IEP plant encounter rate, and IEP plant cover on plant community composition. More complex models with additional terms and interactions between terms were considered prior to model pruning. Environmental factors not included here can be interpreted as being insignificant. Legend: *, 0.01 ≤ *p* < 0.05; **, 0.001 ≤ *p* < 0.01; ***, *p* < 0.001.

Factor	d.f.	F_9,20_	*p*	
Habitat class	3	2.98	<0.0001	***
ln(Patch size)	1	2.14	0.0060	**
Edge:Interior ratio	1	1.18	0.2547	
Woody plant enc. Rate	1	1.74	0.0279	*
ln(Native:IEP encounters)	1	1.98	0.0086	**
IEP plant enc. Rate	1	2.75	<0.0001	***
IEP plant cover	1	0.83	0.6851	
Model	9			

**Table 5:** PerMANCOVA results examining the effects of habitat type, ln(Native:IEP encounters), wind speed, edge to interior ratio, woody plant encounter rate, blooming plant encounter rate, plant diversity, patch size, and temperature on Lepidoptera community composition. More complex models with additional terms and interactions between terms were considered prior to model pruning. Environmental factors not included here can be interpreted as being insignificant. Legend: ., 0.1 ≤ *p* < 0.05; *, 0.01 ≤ *p* < 0.05.

Factor	d.f.	F_11,17_	*p*	
Habitat class	3	1.37	0.0404	*
ln(Native:IEP encounters)	1	1.23	0.1766	
Wind speed	1	1.57	0.0362	*
Edge to interior ratio	1	1.62	0.0223	*
Woody plant enc. rate	1	1.19	0.2178	
Blooming plant enc. rate	1	1.04	0.4003	
Plant diversity	1	1.57	0.0374	*
ln(Patch size)	1	0.97	0.5539	
Temperature	1	1.47	0.0579	.
Model	11			

**Table 6:** PerMANCOVA results examining the effects of habitat type, IEP plant encounter rate, ln(Native:IEP encounters), edge to interior ratio, blooming plant encounter rate, woody plant encounter rate, plant diversity, IEP plant cover, and blooming plant encounter rate on combined plant and Lepidoptera community composition. More complex models with additional terms and interactions between terms were considered prior to model pruning. Environmental factors not included here can be interpreted as being insignificant. Legend: ., 0.05 ≤ *p* < 0.1; *, 0.01 ≤ *p* < 0.05; ***, *p* < 0.001.

Factor	d.f.	F_10,17_	*p*	
Habitat class	3	3.11	0.0001	***
IEP plant enc. rate	1	3.24	0.0001	***
ln(Native:IEP encounters)	1	1.72	0.0293	*
Edge to interior ratio	1	1.84	0.0179	*
Blooming plant enc. rate	1	1.38	0.1149	
Woody plant enc. rate	1	1.68	0.0373	*
IEP plant cover	1	1.15	0.2806	
Blooming plant cover	1	1.51	0.0501	.
Model	10			

**Table 7:** Multiple regression results using Type III sums of squares examining the effects of edge to interior ratio, wind speed, IEP plant encounter rate, temperature, plant diversity, plant richness, and the natural log of the ratio of native to IEP plant encounter rate on total Lepidoptera encounters per 100 m. Legend: *, 0.01 ≤ *p* < 0.05; **, 0.001 ≤ *p* < 0.01; ***, *p* < 0.001.

Factor	d.f.	F_7,20_	*p*	
Edge to interior ratio	1	35.92	0.0000	***
Wind speed	1	10.93	0.0057	**
IEP plant enc. rate	1	6.47	0.0245	*
Temperature	1	5.96	0.0297	*
Plant diversity	1	6.07	0.0285	*
Plant richness	1	2.48	0.1392	
ln(Native:IEP plant enc. rate)	1	1.34	0.2683	
Model	7	5.68	0.0036	**

**Table 8:** Multiple regression results (after model pruning) using Type III sums of squares examining the effects of IEP plant encounter rate, IEP plant cover, blooming plant encounter rate, and wind speed on Lepidoptera diversity (Shannon index). Legend: ., 0.05 ≤ *p* < 0.1; *, 0.01 ≤ *p* < 0.05.

Factor	d.f.	F_4,20_	*p*	
IEP plant enc. rate	1	0.06	0.8151	
IEP plant cover	1	0.80	0.3835	
Blooming plant enc. rate	1	4.91	0.0415	*
Wind speed	1	3.76	0.0705	.
Model	4	2.38	0.0950	.

**Table 9:** List of the 12 most commonly encountered Lepidoptera species observed in this study, with the total number of encounters and migratory status in the LRGV for each. Also shown for each Lepidoptera species are the relationships between the abundance of that species at each study site and (a) key habitat attributes, (b) the abundance of potential host plants, (c) the abundance of plant species in bloom during surveys (i.e., likely nectar sources), and (d) the abundance of plant species with which the Lepidoptera species was observed to be interacting during surveys. All abundance values were normalized based on sampling effort. Each relationship is summarized by its Pearson correlation coefficient (*r*) and its linear model *p*-value (*p*); or by the coefficient of determination (R^2^) instead of *r* where habitat class or multiple environmental variables had a significant relationship with Lepidoptera species abundance. For habitat attributes, the same six core environmental variables were considered as previously, namely habitat class, patch size, edge to interior ratio (EIR), IEP plant cover, IEP plant encounter rate, and ln (Native: IEP encounters). For host plants, known host taxa are named, and then relevant taxa within or related to that group that were observed in this study are denoted in parentheses. Blooming species include the same set of plants as for the blooming plant cover and encounter rate variables considered previously. For observed Lepidoptera-plant interactions, specific plant species that the Lepidoptera taxa was observed feeding from, foraging on, perched upon, or otherwise interacting with are listed. Relationships with invasive grass abundance were also considered, but none were significant, and they are not shown. Full species names and taxonomic information corresponding to the species codes shown here are found in Tables S1 and S2 (the full species lists).

Lep. Species	Enc.	Migrant in LRGV	Habitat Attributes			Host Plant(s)			Blooming Species		Obs. Lep.-plant Interactions		
			Var(s).	*r* or R^2^	*p*	Host Taxa (Observed Taxa)	*r*	*p*	*r*	*p*	Plant spp.	*r*	*p*
*Libytheana carinenta*	18	Yes	Patch size	−0.30	0.19	*Celtis* spp. (*Cel.pal*)	−0.07	0.77	−0.05	0.82	*Sid.cel*, *Pro.gla*	−0.05	0.85
*Pyrisitia lisa*	18	Yes	Patch size	−0.25	0.28	*Chamaecrista* spp. (*Pro.rep)*	0.53	0.01	0.10	0.66	*Mal.ame*, *Ric.bra*, *May.phy*, *Uro.max*	−0.11	0.62
*Danaus gilippus*	13	Yes	EIR	0.27	0.24	*Asclepias* spp. (none obs.)	NA	NA	−0.08	0.73	*Sid.cel*	0.01	0.98
*Mocis latipes*	12	No	EIR	0.68	<0.01	Various row crops (*Sor.bic*)	−0.19	0.41	−0.14	0.55	*Pen.cil*, *Uro.max*	0.05	0.82
*Mocis marcida*	12	No	IEP cover	0.57	0.01	Poaceae (all Poaceae)	0.00	0.99	−0.40	0.70	*Uro.max*	0.26	0.26
*Zerene* *cesonia*	7	Yes	Habitat class	0.64	0.03	Fabaceae (all Fabaceae)	0.34	0.14	0.50	0.02	*Ray.ann*	−0.08	0.74
*Ascia monuste*	6	Yes	IEP enc.	−0.32	0.16	*Brassica* spp. (none obs.)	NA	NA	−0.15	0.53	*Lyc.car*, *Mon.lit*, *Bat.mar*	0.68	<0.01
*Spoladea* *recurvalis*	6	No	EIR, patch size	0.64	<0.01	*Beta* spp. (all Amaranthaceae)	−0.08	0.74	−0.01	0.96	*Uro.max*	−0.10	0.67
*Hemiargus ceraunus*	5	No	IEP enc., IEP cover	0.26	0.06	woody Fabaceae (*Rhynchosia* spp.)	0.93	<0.01	0.19	0.40	*Lan.urt*, *Cyn.bar*, *Rhy.lat*	0.65	<0.01
*Hymenia perspectalis*	5	Yes	Patch size	−0.35	0.12	Amaranthaceae (all Amaranthaceae)	−0.10	0.67	−0.12	0.60	*Dic.ann*, *Cel.pal*, *Zan.fag*	−0.03	0.91
*Panoquina panoquinoides*	5	No	IEP cover	0.25	0.27	Several exotic grasses (*Cyn.dac*)	−0.05	0.85	−0.07	0.78	*Lyc.car*, *Pen.cil*, *Dic.ann*, *Phy.str*	−0.15	0.51
*Phyciodes phaon*	5	No	IEP enc.	0.35	0.12	Verbenaceae (all Verbenaceae)	0.32	0.16	0.40	0.07	*Leu.fru*	0.35	0.13

## Data Availability

The data generated by this study and used for the analyses reported are available upon request from the corresponding author.

## Supplementary Materials

The following are available online at https://www.mdpi.com/article/10.3390/insects12090777/s1; Table S1. Plant species list; Table S2. Lepidoptera species list; Table S3. PerMANCOVA results for Lepidoptera community composition using individual surveys as observations; Table S4. ANCOVA results for the natural log of the ratio of native to IEP plant encounter rates; Table S5. ANCOVA results for plant richness; Table S6. ANCOVA results for total plant cover; Table S7. ANCOVA results for plant diversity; Figure S1. NMDS ordination of plant community composition; Figure S2. NMDS ordination of Lepidoptera community composition using sites as observations; Figure S3. NMDS ordination of Lepidoptera community composition using surveys as observations; Figure S4. NMDS ordination of combined plant and Lepidoptera community composition.

## References

[1] DeFries R., Karanth K.K., Pareeth S. (2010). Interactions between Protected Areas and Their Surroundings in Human-Dominated Tropical Landscapes. Biol. Conserv..

[2] Foley J.A., Ramankutty N., Brauman K.A., Cassidy E.S., Gerber J.S., Johnston M., Mueller N.D., O’Connell C., Ray D.K., West P.C. (2011). Solutions for a Cultivated Planet. Nature.

[3] Blair R.B., Launer A.E. (1997). Butterfly Diversity and Human Land Use: Species Assemblages along an Urban Gradient. Biol. Conserv..

[4] Bonebrake T.C., Sorto R. (2009). Butterfly (Papilionoidea and Hesperioidea) Rapid Assessment of a Coastal Countryside in El Salvador. Trop. Conserv. Sci..

[5] Rudnick D., Ryan S.J., Beier P., Cushman S.A., Dieffenbach F., Epps C., Gerber L.R., Hartter J.N., Jenness J.S., Kintsch J. (2012). The Role of Landscape Connectivity in Planning and Implementing Conservation and Restoration Priorities. Issues in Ecology.

[6] Crooks K.R., Sanjayan M., Crooks K.R., Sanjayan M. (2006). Connectivity conservation: Maintaining connections for nature. Connectivity Conservation.

[7] Franklin J.F., Lindenmayer D.B. (2009). Importance of Matrix Habitats in Maintaining Biological Diversity. Proc. Natl. Acad. Sci. USA.

[8] Jew E.K.K., Loos J., Dougill A.J., Sallu S.M., Benton T.G. (2015). Butterfly Communities in Miombo Woodland: Biodiversity Declines with Increasing Woodland Utilisation. Biol. Conserv..

[9] Kremen C., Colwell R., Erwin T., Murphy D., Noss R., Sanjayan M. (1993). Terrestrial Arthropod Assemblages—Their Use in Conservation Planning. Conserv. Biol..

[10] Kerr J.T., Sugar A., Packer L. (2000). Indicator Taxa, Rapid Biodiversity Assessment, and Nestedness in an Endangered Ecosystem. Conserv. Biol..

[11] Koh L.P., Sodhi N.S. (2004). Importance of Reserves, Fragments, and Parks for Butterfly Conservation in a Tropical Urban Landscape. Ecol. Appl..

[12] Miller J.R., Snyder S.A., Skibbe A.M., Haight R.G. (2009). Prioritizing Conservation Targets in a Rapidly Urbanizing Landscape. Landsc. Urban Plan..

[13] Takacs D. (1996). The Idea of Biodiversity: Philosophies of Paradise.

[14] Fuller T., Munguía M., Mayfield M., Sánchez-Cordero V., Sarkar S. (2006). Incorporating Connectivity into Conservation Planning: A Multi-Criteria Case Study from Central Mexico. Biol. Conserv..

[15] Wilhere G.F. (2008). The How-Much-Is-Enough Myth. Conserv. Biol..

[16] Pellet J., Bried J.T., Parietti D., Gander A., Heer P.O., Cherix D., Arlettaz R. (2012). Monitoring Butterfly Abundance: Beyond Pollard Walks. PLoS ONE.

[17] Tansley A.G. (1935). The Use and Abuse of Vegetational Concepts and Terms. Ecology.

[18] Nelson S., Andersen D. (1994). An Assessment of Riparian Environmental-Quality by Using Butterflies and Disturbance Susceptibility Scores. Southw. Nat..

[19] Medeiros M.J., Eiben J.A., Haines W.P., Kaholoaa R.L., King C.B.A., Krushelnycky P.D., Magnacca K.N., Rubinoff D., Starr F., Starr K. (2013). The Importance of Insect Monitoring to Conservation Actions in Hawaii. Proc. Hawaii. Entomol. Soc..

[20] Kadlec T., Tropek R., Konvicka M. (2012). Timed Surveys and Transect Walks as Comparable Methods for Monitoring Butterflies in Small Plots. J. Insect Conserv..

[21] Ries L., Debinski D.M., Wieland M.L. (2001). Conservation Value of Roadside Prairie Restoration to Butterfly Communities. Conserv. Biol..

[22] Sparrow H.R., Sisk T.D., Ehrlich P.R., Murphy D.D. (1994). Techniques and Guidelines for Monitoring Neotropical Butterflies. Conserv. Biol..

[23] Simonson S.E., Opler P.A., Stohlgren T.J., Chong G.W. (2001). Rapid Assessment of Butterfly Diversity in a Montane Landscape. Biodivers. Conserv..

[24] Tettey C.N.D., Anderson R.S., Kyerematen R. (2020). Rapid Assessment of Butterfly Diversity of Two Proposed Community Resource Management Areas (CREMAs) in the Western North Region of Ghana: Implication for Conservation. Biodiversitas.

[25] Ricketts T., Imhoff M. (2003). Biodiversity, Urban Areas, and Agriculture: Locating Priority Ecoregions for Conservation. Conserv. Ecol..

[26] U.S. Bureau of the Census (1991). Statistical Abstract of the United States: 1991.

[27] Wauer R.H. (2004). Butterflies of the Lower Rio Grande Valley.

[28] Glassberg J. (2017). A Swift Guide to Butterflies of North America.

[29] Glassberg J. (2018). A Swift Guide to Butterflies of Mexico and Central America.

[30] Leckie S., Beadle D. (2018). Peterson Field Guide to Moths of Southeastern North America.

[31] Showler A.T. (2019). Mexican Rice Borer Control Tactics in United States Sugarcane. Insects.

[32] Showler A.T., Reagan T.E. (2017). Mexican Rice Borer, *Eoreuma Loftini* (Dyar) (Lepidoptera: Crambidae): Range Expansion, Biology, Ecology, Control Tactics, and New Resistance Factors in United States Sugarcane. Am. Entomol..

[33] Wilson B.E., Vanweelden M.T., Beuzelin J.M., Reagan T.E., Way M.O., White W.H., Wilson L.T., Showler A.T. (2015). A Relative Resistance Ratio for Evaluation of Mexican Rice Borer (Lepidoptera: Crambidae) Susceptibility Among Sugarcane Cultivars. J. Econ. Entomol..

[34] Showler A.T., Wilson B.E., Reagan T.E. (2012). Mexican Rice Borer (Lepidoptera: Crambidae) Injury to Corn Greater Than to Sorghum and Sugarcane Under Field Conditions. J. Econ. Entomol..

[35] He X.D., Chen W., Liu T.X. (2003). Attraction of Diamondback Moth to Three Commercial Sex Pheromone Lures under Laboratory and Field Conditions. Southw. Entomol..

[36] Greenberg S.M., Adamczyk J.J. (2010). Effectiveness of Transgenic Bt Cottons against Noctuids in the Lower Rio Grande Valley of Texas. Southw. Entomol..

[37] Wagner D.L., Fox R., Salcido D.M., Dyer L.A. (2021). A Window to the World of Global Insect Declines: Moth Biodiversity Trends Are Complex and Heterogeneous. Proc. Natl. Acad. Sci. USA.

[38] Uhl B., Woelfling M., Fiedler K. (2021). Qualitative and Quantitative Loss of Habitat at Different Spatial Scales Affects Functional Moth Diversity. Front. Ecol. Evol..

[39] Clem C.S., Held D.W. (2015). Species Richness of Eruciform Larvae Associated with Native and Alien Plants in the Southeastern United States. J. Insect Conserv..

[40] Stireman J.O., Devlin H., Doyle A.L. (2014). Habitat Fragmentation, Tree Diversity, and Plant Invasion Interact to Structure Forest Caterpillar Communities. Oecologia.

[41] Jahrsdoerfer S.E., Leslie D.M. (1988). Tamaulipan Brushland of the Lower Rio Grande Valley of South. Texas: Description, Human Impacts, and Management Options.

[42] Mathis M., Matisoff D., Pritchett T. (2004). The Economic Value of Water for Ecosystem Preservation: Ecotourism in the Texas Lower Rio Grande Valley.

[43] Woosnam K.M., Dudensing R.M., Hanselka D., An S. (2011). An Initial Examination of the Economic Impact of Nature Tourism on the Rio Grande Valley.

[44] Cariveau A.B., Anderson E., Baum K.A., Hopwood J., Lonsdorf E., Nootenboom C., Tuerk K., Oberhauser K., Snell-Rood E. (2019). Rapid Assessment of Roadsides as Potential Habitat for Monarchs and Other Pollinators. Front. Ecol. Evol..

[45] Leslie D.M. (2016). An International Borderland of Concern: Conservation of Biodiversity in the Lower Rio Grande Valley.

[46] Best C., Van Devender T.R., Espinosa-Garcia F.J., Harper-Lore B.L., Hubbard T. (2006). Fighting weeds with weeds: Battling invasive grasses in the Rio Grande Delta of Texas. Invasive Plants on the Move: Controlling Them in North America; Based on Presentations from Weeds Across Borders 2006 Conference.

[47] Wied J.P., Perotto-Baldivieso H.L., Conkey A.A.T., Brennan L.A., Mata J.M. (2020). Invasive Grasses in South Texas Rangelands: Historical Perspectives and Future Directions. Invasive Plant. Sci. Manag..

[48] Harveson P.M., Tewes M.E., Anderson G.L., Laack L.L. (2004). Habitat Use by Ocelots in South Texas: Implications for Restoration. Wildl. Soc. Bull..

[49] Jackson V.L., Laack L.L., Zimmerman E.G. (2005). Landscape Metrics Associated with Habitat Use by Ocelots in South Texas. J. Wildl. Manag..

[50] Lombardi J.V., Tewes M.E., Perotto-Baldivieso H.L., Mata J.M., Campbell T.A. (2020). Spatial Structure of Woody Cover Affects Habitat Use Patterns of Ocelots in Texas. Mammal. Res..

[51] Cuéllar-Rodríguez G., Jurado E., Flores J. (2017). Beetle Diversity in Fragmented Thornscrub and Isolated Trees. Braz. J. Biol..

[52] Ockinger E., Schweiger O., Crist T.O., Debinski D.M., Krauss J., Kuussaari M., Petersen J.D., Poyry J., Settele J., Summerville K.S. (2010). Life-History Traits Predict Species Responses to Habitat Area and Isolation: A Cross-Continental Synthesis. Ecol. Lett..

[53] Ewers R.M., Didham R.K. (2006). Confounding Factors in the Detection of Species Responses to Habitat Fragmentation. Biol. Rev..

[54] Elliot L.F., Diamond D.D., True C.D., Blodgett C.F., Pursell D., German D., Treuer-Kuehn A. (2014). Ecological Mapping Systems of Texas: Summary Report.

[55] Elliot L. (2014). Descriptions of Systems, Mapping Subsystems, and Vegetation Types for Texas.

[56] Horne J.S., Haines A.M., Tewes M.E., Laack L.L. (2009). Habitat Partitioning by Sympatric Ocelots and Bobcats: Implications for Recovery of Ocelots in Southern Texas. Southw. Nat..

[57] Pollard E., Yates T.J. (1993). Monitoring Butterflies for Ecology and Conservation: The British Butterfly Monitoring Scheme.

[58] Skorka P., Settele J., Woyciechowski M. (2007). Effects of Management Cessation on Grassland Butterflies in Southern Poland. Agric. Ecosyst. Environ..

[59] Haddad N.M. (1999). Corridor and Distance Effects on Interpatch Movements: A Landscape Experiment with Butterflies. Ecol. Appl..

[60] Kariyat R.R. (2018). Personal Communication.

[61] Gehlhausen S.M., Schwartz M.W., Augspurger C.K. (2000). Vegetation and Microclimatic Edge Effects in Two Mixed-Mesophytic Forest Fragments. Plant. Ecol..

[62] Wilhm J.L. (1970). Effect of Sample Size on Shannon’s Formula. Southwest. Nat..

[63] Soetaert K., Heip C. (1990). Sample-Size Dependence of Diversity Indices and the Determination of Sufficient Sample Size in a High-Diversity Deep-Sea Environment. Mar. Ecol. Prog. Ser..

[64] Gotelli N.J., Colwell R.K., Magurran A.E., McGill B.J. (2011). Estimating Species Richness. Biological Diversity: Frontiers in Measurement and Assessment.

[65] Mack R.N., Simberloff D., Lonsdale W.M., Evans H., Clout M., Bazzaz F.A. (2000). Biotic Invasions: Causes, Epidemiology, Global Consequences, and Control. Ecol. Appl..

[66] USDA NRCS The PLANTS Database. Http://Plants.Usda.Gov.

[67] Habel J.C., Trusch R., Schmitt T., Ochse M., Ulrich W. (2019). Long-Term Large-Scale Decline in Relative Abundances of Butterfly and Burnet Moth Species across South-Western Germany. Sci. Rep..

[68] Dirzo R., Young H.S., Galetti M., Ceballos G., Isaac N.J.B., Collen B. (2014). Defaunation in the Anthropocene. Science.

[69] Hobbs R.J., Arico S., Aronson J., Baron J.S., Bridgewater P., Cramer V.A., Epstein P.R., Ewel J.J., Klink C.A., Lugo A.E. (2006). Novel Ecosystems: Theoretical and Management Aspects of the New Ecological World Order. Glob. Ecol. Biogeogr..

[70] Hobbs R.J., Higgs E., Harris J.A. (2009). Novel Ecosystems: Implications for Conservation and Restoration. Trends Ecol. Evol..

[71] Gilbert L.E., Rankin M.A. (1985). Ecological factors which influence migratory behavior in two butterflies of the semi-arid shrublands of South Texas. Migration: Mechanisms and Adaptive Significance.

[72] Dantas de Miranda M., Pereira H.M., Corley M.F.V., Merckx T. (2019). Beta Diversity Patterns Reveal Positive Effects of Farmland Abandonment on Moth Communities. Sci. Rep..

[73] MacDonald Z.G., Anderson I.D., Acorn J.H., Nielsen S.E. (2018). Decoupling Habitat Fragmentation from Habitat Loss: Butterfly Species Mobility Obscures Fragmentation Effects in a Naturally Fragmented Landscape of Lake Islands. Oecologia.

