# Supplementary material for: Effects of Patch Size, Fragmentation, and Invasive Species on Plant and Lepidoptera Communities in Southern Texas
Source: Insects. 2021 Aug 29;12(9):777. doi: 10.3390/insects12090777 (PMC8472066; doi:10.3390/insects12090777)
Supplement: Supplementary file 1 [file insects-12-00777-s001.zip › Figure S3.pdf]

Effects of patch size, fragmentation, and invasive species on plant and Lepidoptera communities in southern Texas
James A. Stilley and Christopher A. Gabler

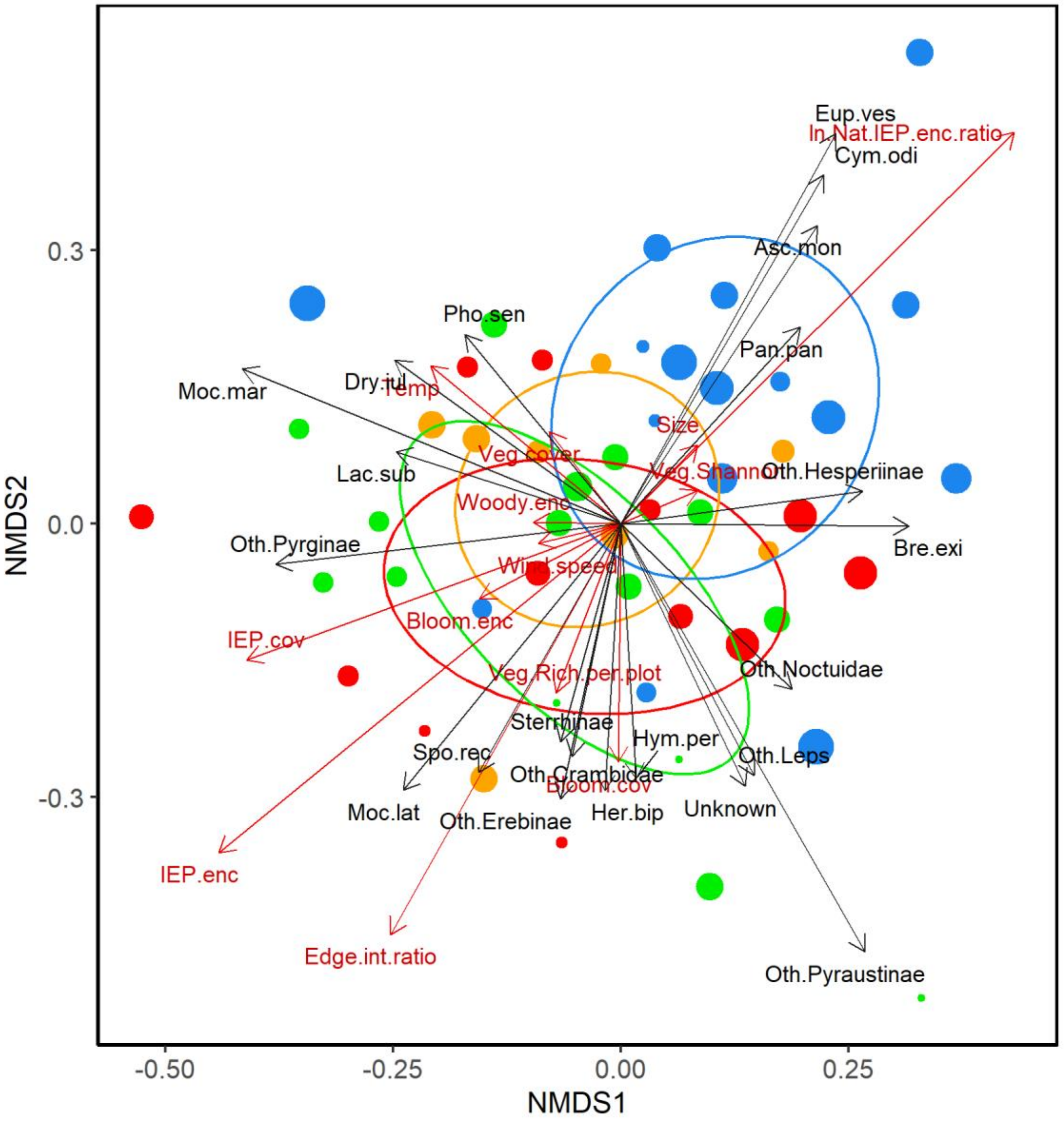

Figure S3. NMDS ordinations representing Lepidoptera community compositions and similarities among observed communities, which are represented as the position and spatial proximity of points, respectively. Points represent observed communites and correspond to individual Lepidoptera surveys. The color and size of points denote habitat class and patch size, as depicted in the inset legend. Black vector arrows denote important species that drove separation among communities in the directions specified based on the observed prevalence of those species. Red vector arrows denote important continuous environmental factors associated with separation among communities in the directions specified. Colored ellipses represent the 95% confidence intervals around the theoretical average communities found in the four focal habitat classes. See Tables S3 (PerMANCOVA results) for additional information related to this ordination. See Table S2 for full species names and higher taxonomic information for Lepidoptera.
