# Supplementary material for: Effects of Patch Size, Fragmentation, and Invasive Species on Plant and Lepidoptera Communities in Southern Texas
Source: Insects. 2021 Aug 29;12(9):777. doi: 10.3390/insects12090777 (PMC8472066; doi:10.3390/insects12090777)
Supplement: Supplementary file 1 [file insects-12-00777-s001.zip › Figure S4.pdf]

Effects of patch size, fragmentation, and invasive species on plant and Lepidoptera communities in southern Texas
James A. Stilley and Christopher A. Gabler

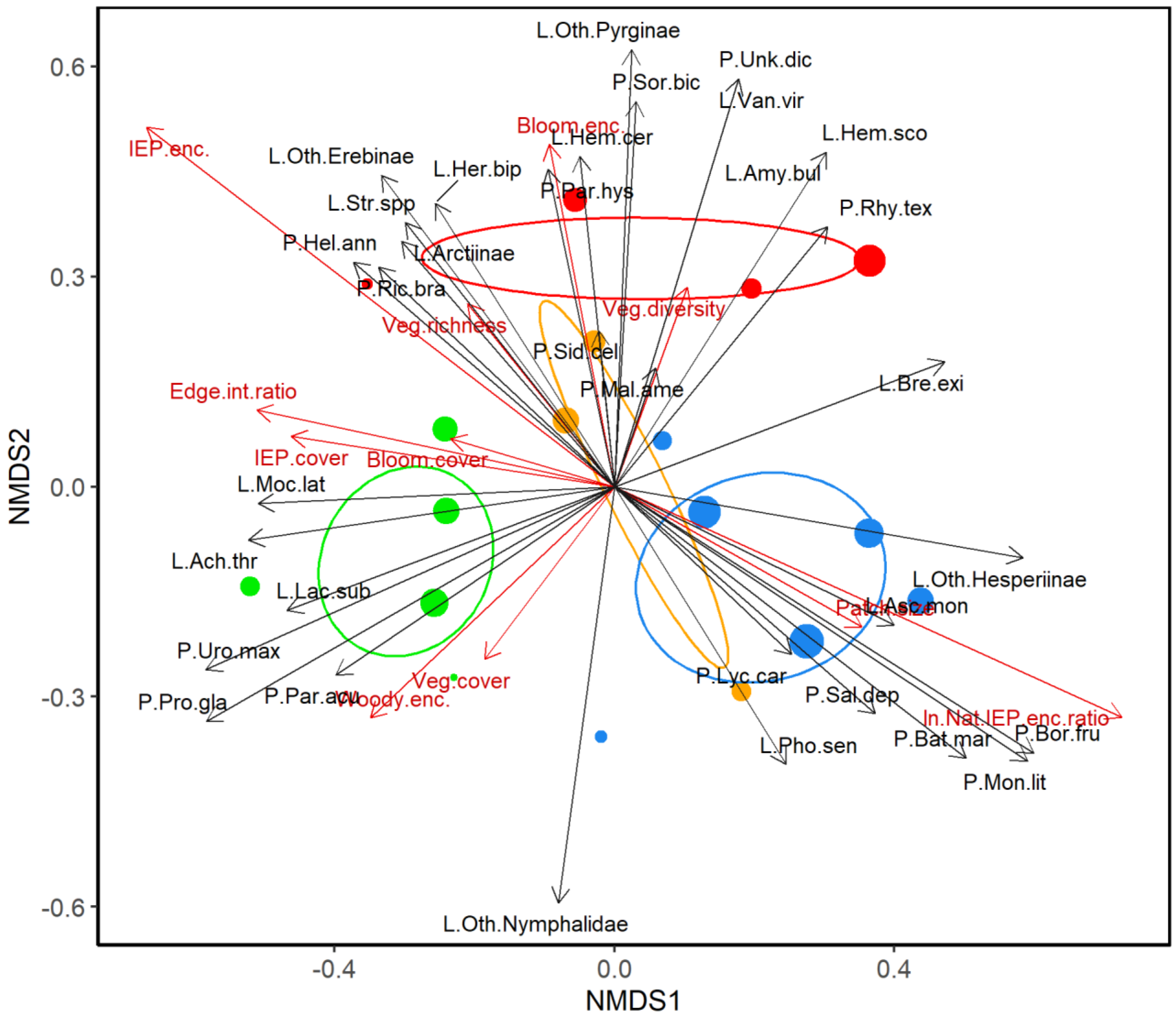

Figure S4. NMDS ordinations representing combined plant and Lepidoptera community compositions and similarities among observed communities, which are represented as the position and spatial proximity of points, respectively. Points represent observed communites and correspond to individual study sites. The color and size of points denote habitat class and patch size, as depicted in the inset legend. Black vector arrows denote important species that drove separation among communities in the directions specified based on the observed prevalence of those species. Red vector arrows denote important continuous environmental factors associated with separation among communities in the directions specified. Colored ellipses represent the 95% confidence intervals around the theoretical average communities found in the four focal habitat classes. See Tables 6 (PerMANCOVA results) for additional information related to this ordination. See Tables S1 and S2 for full species names and higher taxonomic information for plants and Lepidoptera, respectively, and for plant species nativity and pest status.
