# Supplementary material for: Effects of Patch Size, Fragmentation, and Invasive Species on Plant and Lepidoptera Communities in Southern Texas
Source: Insects. 2021 Aug 29;12(9):777. doi: 10.3390/insects12090777 (PMC8472066; doi:10.3390/insects12090777)
Supplement: Supplementary file 1 [file insects-12-00777-s001.zip › Table S1.pdf]

Table S1a. List of observed plant species, with the native and pest status, woody designation, whether it was observed to be in bloom during the survey period, and number of encounters for each taxa, ordered by species.

Code	Family (or group)	Genus	Species	Common name	Native Status	Pest Status	Woody	Obs. Bloom	Encounters
Aca.tet	Cactaceae	*Acanthocereus*	*tetragonus*	Triangle cactus	Native	No	No	No	8
Ama.pol	Amaranthaceae	*Amaranthus*	*polygonoides*	Tropical amaranth	Native	No	No	No	1
Amm.coc	Lythraceae	*Ammannia*	*coccinea*	Valley redstem	Native	No	No	No	5
Ana.arv	Primulaceae	*Anagallis*	*arvensis*	Scarlet pimpernel	Exotic	No	No	No	12
Aph.ski	Asteraceae	*Aphanostephus*	*skirrhobasis*	Arkansas dozedaisy	Native	No	No	No	1
Asc.asp	Asclepiadaceae	*Asclepias*	*asperula*	Spider milkweed	Native	No	No	No	1
Ast.nut	Fabaceae	*Astragalus*	*nuttallianus*	Smallflower milkvetch	Native	No	No	No	1
Aye.lim	Sterculiaceae	*Ayenia*	*limitaris*	Rio Grande ayenia	Native	No	No	No	1
Bac.neg	Asteraceae	*Baccharis*	*neglecta*	Rooseveltweed	Native	No	No	No	2
Bat.mar	Bataceae	*Batis*	*maritima*	Turtleweed	Native	No	No	No	38
Bol.mar	Cyperaceae	*Bolboschoenus*	*maritimus*	Cosmopolitan bulrush	Native	No	No	No	2
Bor.fru	Asteraceae	*Borrichia*	*frutescens*	Bushy seaside tansy	Native	No	No	No	108
Bot.isc	Poaceae	*Bothriochloa*	*ischaemum*	Yellow bluestem	Exotic	Yes	No	No	7
Bud.ses	Buddlejaceae	*Buddleja*	*sessiliflora*	Rio Grande butterflybush	Native	No	No	No	1
Cas.ere	Simaroubaceae	*Castela*	*erecta*	Goatbush	Native	No	Yes	No	1
Cel.pal	Cannabaceae	*Celtis*	*pallida*	Granjeno	Native	No	Yes	No	12
Cen.asi	Apiaceae	*Centella*	*asiatica*	Spadeleaf	Exotic	No	No	No	3
Cha.cor	Euphorbiaceae	*Chamaesyce*	*cordifolia*	Heartleaf sandmat	Native	No	No	No	1
Cha.gly	Euphorbiaceae	*Chamaesyce*	*glyptosperma*	Ribseed sandmat	Native	No	No	No	1
Cha.hum	Euphorbiaceae	*Chamaesyce*	*humistrata*	Spreading sandmat	Native	No	No	No	8
Cha.mac	Euphorbiaceae	*Chamaesyce*	*maculata*	Spotted sandmat	Native	No	No	No	17
Chi.alb	Rubiaceae	*Chiococca*	*alba*	West Indian milkberry	Native	No	Yes	No	1
Chr.odo	Asteraceae	*Chromolaena*	*odorata*	Crucita blue mistflower	Native	No	No	Yes	7
Cic.sp.	Apiaceae	*Cicuta*	sp.	Water hemlock	Native	No	No	No	1
Cis.tri	Vitaceae	*Cissus*	*trifoliata*	Sorrelvine	Native	No	No	No	6
Cit.ber	Verbenaceae	*Citharexylum*	*berlandieri*	Berlandier fiddlewood	Native	No	Yes	No	3
Cla.sua	Asteraceae	*Clappia*	*suaedifolia*	Fleshy clapdaisy	Native	No	No	No	1
Con.hoo	Rhamnaceae	*Condalia*	*hookeri*	Brazilian bluewood	Native	No	Yes	No	5
Con.bet	Asteraceae	*Conoclinium*	*betonicifolium*	Mist flower	Native	No	No	No	2
Con.coe	Asteraceae	*Conoclinium*	*coelestinum*	Blue mistflower	Native	No	No	No	1
Con.ram	Asteraceae	*Conyza*	*ramosissima*	Dwarf horseweed	Native	No	No	No	1
Cor.sp.	Asteraceae	*Coreopsis*	sp.	Tickseed	Native	No	No	No	4
Cre.nud	Convolvulaceae	*Cressa*	*nudicaulis*	Nakedstem alkaliweed	Native	No	No	No	1

Cro.cap	Euphorbiaceae	*Croton*	*capitatus*	Lindheimer's hogwort	Native	No	No	No	1
Cro.hum	Euphorbiaceae	*Croton*	*humilis*	Pepperbush	Native	No	No	No	1
Cro.sp.	Euphorbiaceae	*Croton*	sp.	Croton	Native	No	No	No	2
Cyc.lep	Apiaceae	*Cyclospermum*	*leptophyllum*	Marsh parsley	Exotic	No	No	No	16
Cyn.bar	Asclepiadaceae	*Cynanchum*	*barbigerum*	Bearded swallow-wort	Native	No	No	No	21
Cyn.dac	Poaceae	*Cynodon*	*dactylon*	Bermudagrass	Exotic	Yes	No	No	42
Cyp.art	Cyperaceae	*Cyperus*	*articulatus*	Jointed flatsedge	Native	No	No	No	10
Cyp.era	Cyperaceae	*Cyperus*	*eragrostis*	Tall Flatsedge	Native	No	No	No	6
Cyp.esc	Cyperaceae	*Cyperus*	*esculentus*	Yellow nutsedge	Exotic	No	No	No	5
Dic.ann	Poaceae	*Dichanthium*	*annulatum*	Diaz bluestem	Exotic	Yes	No	No	2
Dig.san	Poaceae	*Digitaria*	*sanguinalis*	Hairy crabgrass	Exotic	Yes	No	No	6
Dio.rad	Rubiaceae	*Diodia*	*radula*	Rough buttonweed	Exotic	No	No	No	2
Ebe.eba	Fabaceae	*Ebenopsis*	*ebano*	Texas ebony	Native	No	Yes	No	10
Ehr.ana	Boraginaceae	*Ehretia*	*anacua*	Knockaway	Native	No	Yes	No	2
Ele.sp.	Cyperaceae	*Eleocharis*	sp.	Spikerush	Native	No	No	No	3
Evo.als	Convolvulaceae	*Evolvulus*	*alsinoides*	Slender dwarf morning glory	Native	No	No	No	1
Fim.cas	Cyperaceae	*Fimbristylis*	*castanea*	Marsh fimbry	Native	No	No	No	1
Flo.tri	Asteraceae	*Florestina*	*tripteris*	Sticky florestina	Native	No	No	No	4
For.ang	Oleaceae	*Forestiera*	*angustifolia*	Texas swampprivet	Native	No	Yes	No	5
Gna.pen	Asteraceae	*Gamochaeta*	*pensylvanica*	Pennsylvania everlasting	Native	No	No	No	1
Gla.bip	Verbenaceae	*Glandularia*	*bipinnatifida*	Dakota mock vervain	Native	No	No	No	7
Gla.pum	Verbenaceae	*Glandularia*	*pumila*	Pink mock vervain	Native	No	No	No	2
Gna.fal	Asteraceae	*Gnaphalium*	*falcatum*	narrowleaf purple everlasting	Native	No	No	No	2
Gom.nea	Amaranthaceae	*Gomphrena*	*nealleyi*	Nealley's globe amaranth	Native	No	No	No	1
Hei.sal	Lythraceae	*Heimia*	*salicifolia*	Shrubby yellowcrest	Native	No	No	No	1
Hel.ann	Asteraceae	*Helianthus*	*annuus*	Common sunflower	Native	No	No	Yes	2
Ibe.lin	Cucurbitaceae	*Ibervillea*	*lindheimeri*	Lindheimer's globeberry	Native	No	No	No	1
Ibe.ten	Cucurbitaceae	*Ibervillea*	*tenuisecta*	Slimlobe globeberry	Native	No	No	No	1
Ipo.cor	Convolvulaceae	*Ipomoea*	*cordatotriloba*	Tievine	Native	No	No	No	1
Ipo.hed	Convolvulaceae	*Ipomoea*	*hederacea*	Ivyleaf morningglory	Exotic	No	No	No	8
Kal.dai	Crassulaceae	*Kalanchoe*	*daigremontiana*	Devil's backbone	Exotic	Yes	No	No	3
Kar.hum	Rhamnaceae	*Karwinskia*	*humboldtiana*	Coyotillo	Native	No	Yes	No	2
Lan.urt	Verbenaceae	*Lantana*	*urticoides*	Texas lantana	Native	No	No	Yes	13
Leu.fru	Scrophulariaceae	*Leucophyllum*	*frutescens*	Texas barometer bush	Native	No	Yes	Yes	5
Lim.car	Plumbaginaceae	*Limonium*	*carolinianum*	Lavender thrift	Native	No	No	No	5
Lyc.ber	Solanaceae	*Lycium*	*berlandieri*	Berlandier's wolfberry	Native	No	No	No	1
Lyc.car	Solanaceae	*Lycium*	*carolinianum*	Carolina desert-thorn	Native	No	No	Yes	8

Lyt.ala	Lythraceae	*Lythrum*	*alatum*	Winged lythrum	Native	No	No	No	2
Lyt.cal	Lythraceae	*Lythrum*	*californicum*	California loosestrife	Native	No	No	No	2
Mal.par	Malvaceae	*Malva*	*parviflora*	Cheeseweed mallow	Exotic	No	No	No	2
Mal.ame	Malvaceae	*Malvastrum*	*americanum*	Indian valley false mallow	Native	No	No	Yes	14
Mar.ves	Marsileaceae	*Marsilea*	*vestita*	Hairy waterclover	Native	No	No	No	3
May.phy	Celastraceae	*Maytenus*	*phyllanthoides*	Florida mayten	Native	No	Yes	No	15
Mec.pro	Scrophulariaceae	*Mecardonia*	*procumbens*	Baby jump-up	Native	No	No	No	1
Men.het	Oleaceae	*Menodora*	*heterophylla*	Low menodora	Native	No	No	No	11
Mol.ver	Molluginaceae	*Mollugo*	*verticillata*	green carpetweed	Native	No	No	No	1
Mon.lit	Poaceae	*Monanthochloe*	*littoralis*	Shoregrass	Native	No	No	No	63
Mon.fru	Lamiaceae	*Monarda*	*fruticulosa*	Spotted beebalm	Native	No	No	No	1
Nam.par	Boraginaceae	*Nama*	*parvifolia*	Small-leaf fiddleleaf	Native	No	No	No	1
Nep.pub	Fabaceae	*Neptunia*	*pubescens*	Tropical puff	Native	No	No	No	15
Oen.spe	Onagraceae	*Oenothera*	*speciosa*	Pinkladies	Native	No	No	No	1
Opu.eng	Cactaceae	*Opuntia*	*engelmannii*	Texas pricklypear	Native	No	No	Yes	16
Opu.lep	Cactaceae	*Opuntia*	*leptocaulis*	Christmas cactus	Native	No	No	No	3
Oxa.dic	Oxalidaceae	*Oxalis*	*dichondrifolia*	Peonyleaf woodsorrel	Native	No	No	No	2
Oxa.str	Oxalidaceae	*Oxalis*	*stricta*	Common yellow oxalis	Native	No	No	No	31
Par.acu	Fabaceae	*Parkinsonia*	*aculeata*	Jerusalem thorn	Native	No	Yes	No	6
Par.con	Asteraceae	*Parthenium*	*confertum*	Gray's feverfew	Native	No	No	No	1
Par.hys	Asteraceae	*Parthenium*	*hysterophorus*	Santa Maria feverfew	Uncertain	Yes	No	Yes	65
Pen.cil	Poaceae	*Pennisetum*	*ciliare*	Buffelgrass	Exotic	Yes	No	No	47
Phy.pol	Euphorbiaceae	*Phyllanthus*	*polygonoides*	Smartweed leafflower	Native	No	No	No	5
Pla.sp.	Plantaginaceae	*Plantago*	sp.	Plantain	Unknown	Unknown	No	No	1
Por.ole	Portulacaceae	*Portulaca*	*oleracea*	Little hogweed	Exotic	Yes	No	No	1
Pro.lou	Pedaliaceae	*Proboscidea*	*louisianica*	Ram's horn	Native	No	No	No	2
Pro.gla	Fabaceae	*Prosopis*	*glandulosa*	Honey mesquite	Native	Yes	Yes	No	31
Pro.rep	Fabaceae	*Prosopis*	*reptans*	Tornillo	Native	No	No	No	38
Ran.rha	Rubiaceae	*Randia*	*rhagocarpa*	Crucillo	Native	No	Yes	No	3
Ray.ann	Asteraceae	*Rayjacksonia*	*annua*	Viscid tansyaster	Native	No	No	No	10
Rhy.ame	Fabaceae	*Rhynchosia*	*americana*	American snoutbean	Native	No	No	No	3
Rhy.lat	Fabaceae	*Rhynchosia*	*latifolia*	Prairie snoutbean	Native	No	No	No	17
Rhy.tex	Fabaceae	*Rhynchosia*	*texana*	Texas snoutbean	Native	No	No	No	2
Rhy.col	Cyperaceae	*Rhynchospora*	*colorata*	Starrush whitetop	Native	No	No	No	2
Ric.bra	Rubiaceae	*Richardia*	*brasiliensis*	Tropical Mexican clover	Exotic	No	No	No	62
Rud.hir	Asteraceae	*Rudbeckia*	*hirta*	Blackeyed Susan	Native	No	No	No	1
Rue.cae	Acanthaceae	*Ruellia*	*caerulea*	Britton's wild petunia	Exotic	Yes	No	No	1

Rue.nud	Acanthaceae	*Ruellia*	*nudiflora*	Violet wild petunia	Native	No	No	No	6
Rue.run	Acanthaceae	*Ruellia*	*nudiflora* var. *runyonii*	Runyon's wild petunia	Native	No	No	No	1
Rue.sp.	Acanthaceae	*Ruellia*	sp.	Wild petunia	Unknown	Unknown	No	No	2
Sal.dep	Chenopodiaceae	*Salicornia*	*depressa*	Virginia glasswort	Native	No	No	No	30
Sal.bal	Lamiaceae	*Salvia*	*ballotaeflora*	Shrubby blue sage	Native	No	No	No	2
Sch.cun	Celastraceae	*Schaefferia*	*cuneifolia*	Desert yaupon	Native	No	Yes	No	2
Scu.ocm	Lamiaceae	*Scutellaria*	*ocmulgee*	Ocmulgee skullcap	Exotic	No	No	No	2
Ses.her	Fabaceae	*Sesbania*	*herbacea*	Bigpod sesbania	Native	No	Yes	No	2
Ses.por	Aizoaceae	*Sesuvium*	*portulacastrum*	Shoreline seapurslane	Native	No	No	No	1
Ses.ses	Aizoaceae	*Sesuvium*	*sessile*	Western seapurslane	Native	No	No	No	1
Set.leu	Poaceae	*Setaria*	*leucopila*	Streambed bristlegrass	Native	No	No	No	4
Sid.acu	Malvaceae	*Sida*	*acuta*	Common wireweed	Native	No	No	No	7
Sid.cor	Malvaceae	*Sida*	*cordifolia*	flannel weed	Native	No	No	No	3
Sid.lin	Malvaceae	*Sida*	*lindheimeri*	Showy fanpetals	Native	No	No	No	1
Sid.rho	Malvaceae	*Sida*	*rhombifolia*	Cuban jute	Native	No	No	No	3
Sid.sp.	Malvaceae	*Sida*	sp.	Fanpetal	Native	No	No	No	2
Sid.cel	Sapotaceae	*Sideroxylon*	*celastrinum*	Saffron plum	Native	No	Yes	Yes	2
Smi.sp.	Smilacaeae	*Smilax*	sp.	Greenbriar	Native	No	No	No	2
Sol.ela	Solanaceae	*Solanum*	*elaeagnifolium*	Silverleaf nightshade	Native	No	No	No	6
Sol.sp.	Solanaceae	*Solanum*	sp.	Nightshade	Unknown	Unknown	No	No	2
Son.ole	Asteraceae	*Sonchus*	*oleraceus*	Common sowthistle	Exotic	No	No	No	1
Son.sp.	Asteraceae	*Sonchus*	sp.	Sowthistle	Unknown	Unknown	No	No	1
Sor.bic	Poaceae	*Sorghum*	*bicolor*	grain sorghum	Exotic	No	No	No	58
Spa.spa	Poaceae	*Spartina*	*spartinae*	Gulf cordgrass	Native	No	No	No	21
Sph.sp.	Malvaceae	*Sphaeralcea*	sp.	Globemallow	Native	No	No	No	1
Spo.cry	Poaceae	*Sporobolus*	*cryptandrus*	Spike dropseed	Native	No	No	No	1
Spo.vir	Poaceae	*Sporobolus*	*virginicus*	Seashore dropseed	Native	No	No	No	3
Spo.wri	Poaceae	*Sporobolus*	*wrightii*	Big sacaton	Native	No	No	No	1
Sta.dru	Lamiaceae	*Stachys*	*drummondii*	Drummond's hedgenettle	Native	No	No	No	4
Sua.lin	Chenopodiaceae	*Suaeda*	*linearis*	Annual seepweed	Native	No	No	No	23
Tam.azu	Asteraceae	*Tamaulipa*	*azurea*	Blue bonset	Native	No	No	No	3
Tar.off	Asteraceae	*Taraxacum*	*officinale*	Common dandelion	Exotic	Yes	No	No	12
Teu.cub	Lamiaceae	*Teucrium*	*cubense*	Small coastal germander	Native	No	No	No	10
Teu.sp.	Lamiaceae	*Teucrium*	sp.	Germander	Native	No	No	No	1
Uro.max	Poaceae	*Urochloa*	*maxima*	Guineagrass	Exotic	Yes	No	No	84
Uro.pla	Poaceae	*Urochloa*	*platyphylla*	Broadleaf signalgrass	Native	Mixed	No	No	2
Vac.far	Fabaceae	*Vachellia*	*farnesiana*	Sweet acacia	Native	Yes	Yes	No	5

Ver.hal	Verbenaceae	*Verbena*	*halei*	Texas vervain	Native	No	No	No	3
Ver.pli	Verbenaceae	*Verbena*	*plicata*	Fanleaf vervain	Native	No	No	No	1
Vib.den	Caprifoliaceae	*Viburnum*	*dentatum*	Southern arrowwood	Native	No	Yes	No	3
Vig.lut	Fabaceae	*Vigna*	*luteola*	Hairypod cowpea	Native	No	No	No	3
Vig.ste	Asteraceae	*Viguiera*	*stenoloba*	Resinbush	Native	No	No	No	4
Xan.str	Asteraceae	*Xanthium*	*strumarium*	Rough cocklebur	Native	Yes	No	No	7
Xan.sp.	Asteraceae	*Xanthocephalum*	sp.	Yellow head aster	Native	No	No	No	1
Yuc.tre	Agavaceae	*Yucca*	*treculeana*	Don Quixote's lace	Native	No	Yes	No	1
Zan.fag	Rutaceae	*Zanthoxylum*	*fagara*	Lime pricklyash	Native	No	Yes	No	10
Asteraceae	Asteraceae			Aster family	Mixed	Mixed	No	No	4
Caryophyllaceae	Caryophyllaceae			Carnation family	Unknown	Unknown	No	No	1
Fabaceae	Fabaceae			Legume family	Mixed	Mixed	Mixed	No	1
Unk.dic	Dicot			Unknown dicot	Unknown	Unknown		No	23
Unk.mon	Monocot			Unknown monocot	Unknown	Unknown		No	9
Unknown	Unknown			Unknown	Unknown	Unknown		No	31

Table S1b. List of observed plant species, with the native and pest status, woody designation, whether it was observed to be in bloom during the survey period, and number of encounters for each taxa, ordered by family.

Code	Family (or group)	Genus	Species	Common name	Native Status	Pest Status	Woody	Obs. Bloom	Encounters
Rue.cae	Acanthaceae	*Ruellia*	*caerulea*	Britton's wild petunia	Exotic	Yes	No	No	1
Rue.nud	Acanthaceae	*Ruellia*	*nudiflora*	Violet wild petunia	Native	No	No	No	6
Rue.run	Acanthaceae	*Ruellia*	*nudiflora* var. *runyonii*	Runyon's wild petunia	Native	No	No	No	1
Rue.sp.	Acanthaceae	*Ruellia*	sp.	Wild petunia	Unknown	Unknown	No	No	2
Yuc.tre	Agavaceae	*Yucca*	*treculeana*	Don Quixote's lace	Native	No	Yes	No	1
Ses.por	Aizoaceae	*Sesuvium*	*portulacastrum*	Shoreline seapurslane	Native	No	No	No	1
Ses.ses	Aizoaceae	*Sesuvium*	*sessile*	Western seapurslane	Native	No	No	No	1
Ama.pol	Amaranthaceae	*Amaranthus*	*polygonoides*	Tropical amaranth	Native	No	No	No	1
Gom.nea	Amaranthaceae	*Gomphrena*	*nealleyi*	Nealley's globe amaranth	Native	No	No	No	1
Cen.asi	Apiaceae	*Centella*	*asiatica*	Spadeleaf	Exotic	No	No	No	3
Cic.sp.	Apiaceae	*Cicuta*	sp.	Water hemlock	Native	No	No	No	1
Cyc.lep	Apiaceae	*Cyclospermum*	*leptophyllum*	Marsh parsley	Exotic	No	No	No	16
Asc.asp	Asclepiadaceae	*Asclepias*	*asperula*	Spider milkweed	Native	No	No	No	1
Cyn.bar	Asclepiadaceae	*Cynanchum*	*barbigerum*	Bearded swallow-wort	Native	No	No	No	21
Aph.ski	Asteraceae	*Aphanostephus*	*skirrhobasis*	Arkansas dozedaisy	Native	No	No	No	1
Bac.neg	Asteraceae	*Baccharis*	*neglecta*	Rooseveltweed	Native	No	No	No	2
Bor.fru	Asteraceae	*Borrichia*	*frutescens*	Bushy seaside tansy	Native	No	No	No	108
Chr.odo	Asteraceae	*Chromolaena*	*odorata*	Crucita blue mistflower	Native	No	No	Yes	7
Cla.sua	Asteraceae	*Clappia*	*suaedifolia*	Fleshy clapdaisy	Native	No	No	No	1
Con.bet	Asteraceae	*Conoclinium*	*betonicifolium*	Mist flower	Native	No	No	No	2
Con.coe	Asteraceae	*Conoclinium*	*coelestinum*	Blue mistflower	Native	No	No	No	1
Con.ram	Asteraceae	*Conyza*	*ramosissima*	Dwarf horseweed	Native	No	No	No	1
Cor.sp.	Asteraceae	*Coreopsis*	sp.	Tickseed	Native	No	No	No	4
Flo.tri	Asteraceae	*Florestina*	*tripteris*	Sticky florestina	Native	No	No	No	4
Gna.pen	Asteraceae	*Gamochaeta*	*pensylvanica*	Pennsylvania everlasting	Native	No	No	No	1
Gna.fal	Asteraceae	*Gnaphalium*	*falcatum*	narrowleaf purple everlasting	Native	No	No	No	2
Hel.ann	Asteraceae	*Helianthus*	*annuus*	Common sunflower	Native	No	No	Yes	2
Par.con	Asteraceae	*Parthenium*	*confertum*	Gray's feverfew	Native	No	No	No	1
Par.hys	Asteraceae	*Parthenium*	*hysterophorus*	Santa Maria feverfew	Uncertain	Yes	No	Yes	65
Ray.ann	Asteraceae	*Rayjacksonia*	*annua*	Viscid tansyaster	Native	No	No	No	10
Rud.hir	Asteraceae	*Rudbeckia*	*hirta*	Blackeyed Susan	Native	No	No	No	1
Son.ole	Asteraceae	*Sonchus*	*oleraceus*	Common sowthistle	Exotic	No	No	No	1
Son.sp.	Asteraceae	*Sonchus*	sp.	Sowthistle	Unknown	Unknown	No	No	1

Tam.azu	Asteraceae	*Tamaulipa*	*azurea*	Blue bonset	Native	No	No	No	3
Tar.off	Asteraceae	*Taraxacum*	*officinale*	Common dandelion	Exotic	Yes	No	No	12
Vig.ste	Asteraceae	*Viguiera*	*stenoloba*	Resinbush	Native	No	No	No	4
Xan.str	Asteraceae	*Xanthium*	*strumarium*	Rough cocklebur	Native	Yes	No	No	7
Xan.sp.	Asteraceae	*Xanthocephalum*	sp.	Yellow head aster	Native	No	No	No	1
Asteraceae	Asteraceae			Aster family	Mixed	Mixed	No	No	4
Bat.mar	Bataceae	*Batis*	*maritima*	Turtleweed	Native	No	No	No	38
Ehr.ana	Boraginaceae	*Ehretia*	*anacua*	Knockaway	Native	No	Yes	No	2
Nam.par	Boraginaceae	*Nama*	*parvifolia*	Small-leaf fiddleleaf	Native	No	No	No	1
Bud.ses	Buddlejaceae	*Buddleja*	*sessiliflora*	Rio Grande butterflybush	Native	No	No	No	1
Aca.tet	Cactaceae	*Acanthocereus*	*tetragonus*	Triangle cactus	Native	No	No	No	8
Opu.eng	Cactaceae	*Opuntia*	*engelmannii*	Texas pricklypear	Native	No	No	Yes	16
Opu.lep	Cactaceae	*Opuntia*	*leptocaulis*	Christmas cactus	Native	No	No	No	3
Cel.pal	Cannabaceae	*Celtis*	*pallida*	Granjeno	Native	No	Yes	No	12
Vib.den	Caprifoliaceae	*Viburnum*	*dentatum*	Southern arrowwood	Native	No	Yes	No	3
Caryophyllaceae	Caryophyllaceae			Carnation family	Unknown	Unknown	No	No	1
May.phy	Celastraceae	*Maytenus*	*phyllanthoides*	Florida mayten	Native	No	Yes	No	15
Sch.cun	Celastraceae	*Schaefferia*	*cuneifolia*	Desert yaupon	Native	No	Yes	No	2
Sal.dep	Chenopodiaceae	*Salicornia*	*depressa*	Virginia glasswort	Native	No	No	No	30
Sua.lin	Chenopodiaceae	*Suaeda*	*linearis*	Annual seepweed	Native	No	No	No	23
Cre.nud	Convolvulaceae	*Cressa*	*nudicaulis*	Nakedstem alkaliweed	Native	No	No	No	1
Evo.als	Convolvulaceae	*Evolvulus*	*alsinoides*	Slender dwarf morning glory	Native	No	No	No	1
Ipo.cor	Convolvulaceae	*Ipomoea*	*cordatotriloba*	Tievine	Native	No	No	No	1
Ipo.hed	Convolvulaceae	*Ipomoea*	*hederacea*	Ivyleaf morningglory	Exotic	No	No	No	8
Kal.dai	Crassulaceae	*Kalanchoe*	*daigremontiana*	Devil's backbone	Exotic	Yes	No	No	3
Ibe.lin	Cucurbitaceae	*Ibervillea*	*lindheimeri*	Lindheimer's globeberry	Native	No	No	No	1
Ibe.ten	Cucurbitaceae	*Ibervillea*	*tenuisecta*	Slimlobe globeberry	Native	No	No	No	1
Bol.mar	Cyperaceae	*Bolboschoenus*	*maritimus*	Cosmopolitan bulrush	Native	No	No	No	2
Cyp.art	Cyperaceae	*Cyperus*	*articulatus*	Jointed flatsedge	Native	No	No	No	10
Cyp.era	Cyperaceae	*Cyperus*	*eragrostis*	Tall Flatsedge	Native	No	No	No	6
Cyp.esc	Cyperaceae	*Cyperus*	*esculentus*	Yellow nutsedge	Exotic	No	No	No	5
Ele.sp.	Cyperaceae	*Eleocharis*	sp.	Spikerush	Native	No	No	No	3
Fim.cas	Cyperaceae	*Fimbristylis*	*castanea*	Marsh fimbry	Native	No	No	No	1
Rhy.col	Cyperaceae	*Rhynchospora*	*colorata*	Starrush whitetop	Native	No	No	No	2
Cha.cor	Euphorbiaceae	*Chamaesyce*	*cordifolia*	Heartleaf sandmat	Native	No	No	No	1
Cha.gly	Euphorbiaceae	*Chamaesyce*	*glyptosperma*	Ribseed sandmat	Native	No	No	No	1
Cha.hum	Euphorbiaceae	*Chamaesyce*	*humistrata*	Spreading sandmat	Native	No	No	No	8

Cha.mac	Euphorbiaceae	*Chamaesyce*	*maculata*	Spotted sandmat	Native	No	No	No	17
Cro.cap	Euphorbiaceae	*Croton*	*capitatus*	Lindheimer's hogwort	Native	No	No	No	1
Cro.hum	Euphorbiaceae	*Croton*	*humilis*	Pepperbush	Native	No	No	No	1
Cro.sp.	Euphorbiaceae	*Croton*	sp.	Croton	Native	No	No	No	2
Phy.pol	Euphorbiaceae	*Phyllanthus*	*polygonoides*	Smartweed leafflower	Native	No	No	No	5
Ast.nut	Fabaceae	*Astragalus*	*nuttallianus*	Smallflower milkvetch	Native	No	No	No	1
Ebe.eba	Fabaceae	*Ebenopsis*	*ebano*	Texas ebony	Native	No	Yes	No	10
Nep.pub	Fabaceae	*Neptunia*	*pubescens*	Tropical puff	Native	No	No	No	15
Par.acu	Fabaceae	*Parkinsonia*	*aculeata*	Jerusalem thorn	Native	No	Yes	No	6
Pro.gla	Fabaceae	*Prosopis*	*glandulosa*	Honey mesquite	Native	Yes	Yes	No	31
Pro.rep	Fabaceae	*Prosopis*	*reptans*	Tornillo	Native	No	No	No	38
Rhy.ame	Fabaceae	*Rhynchosia*	*americana*	American snoutbean	Native	No	No	No	3
Rhy.lat	Fabaceae	*Rhynchosia*	*latifolia*	Prairie snoutbean	Native	No	No	No	17
Rhy.tex	Fabaceae	*Rhynchosia*	*texana*	Texas snoutbean	Native	No	No	No	2
Ses.her	Fabaceae	*Sesbania*	*herbacea*	Bigpod sesbania	Native	No	Yes	No	2
Vac.far	Fabaceae	*Vachellia*	*farnesiana*	Sweet acacia	Native	Yes	Yes	No	5
Vig.lut	Fabaceae	*Vigna*	*luteola*	Hairypod cowpea	Native	No	No	No	3
Fabaceae	Fabaceae			Legume family	Mixed	Mixed	Mixed	No	1
Mon.fru	Lamiaceae	*Monarda*	*fruticulosa*	Spotted beebalm	Native	No	No	No	1
Sal.bal	Lamiaceae	*Salvia*	*ballotaeflora*	Shrubby blue sage	Native	No	No	No	2
Scu.ocm	Lamiaceae	*Scutellaria*	*ocmulgee*	Ocmulgee skullcap	Exotic	No	No	No	2
Sta.dru	Lamiaceae	*Stachys*	*drummondii*	Drummond's hedgenettle	Native	No	No	No	4
Teu.cub	Lamiaceae	*Teucrium*	*cubense*	Small coastal germander	Native	No	No	No	10
Teu.sp.	Lamiaceae	*Teucrium*	sp.	Germander	Native	No	No	No	1
Amm.coc	Lythraceae	*Ammannia*	*coccinea*	Valley redstem	Native	No	No	No	5
Hei.sal	Lythraceae	*Heimia*	*salicifolia*	Shrubby yellowcrest	Native	No	No	No	1
Lyt.ala	Lythraceae	*Lythrum*	*alatum*	Winged lythrum	Native	No	No	No	2
Lyt.cal	Lythraceae	*Lythrum*	*californicum*	California loosestrife	Native	No	No	No	2
Mal.par	Malvaceae	*Malva*	*parviflora*	Cheeseweed mallow	Exotic	No	No	No	2
Mal.ame	Malvaceae	*Malvastrum*	*americanum*	Indian valley false mallow	Native	No	No	Yes	14
Sid.acu	Malvaceae	*Sida*	*acuta*	Common wireweed	Native	No	No	No	7
Sid.cor	Malvaceae	*Sida*	*cordifolia*	flannel weed	Native	No	No	No	3
Sid.lin	Malvaceae	*Sida*	*lindheimeri*	Showy fanpetals	Native	No	No	No	1
Sid.rho	Malvaceae	*Sida*	*rhombifolia*	Cuban jute	Native	No	No	No	3
Sid.sp.	Malvaceae	*Sida*	sp.	Fanpetal	Native	No	No	No	2
Sph.sp.	Malvaceae	*Sphaeralcea*	sp.	Globemallow	Native	No	No	No	1
Mar.ves	Marsileaceae	*Marsilea*	*vestita*	Hairy waterclover	Native	No	No	No	3

Mol.ver	Molluginaceae	*Mollugo*	*verticillata*	green carpetweed	Native	No	No	No	1
For.ang	Oleaceae	*Forestiera*	*angustifolia*	Texas swampprivet	Native	No	Yes	No	5
Men.het	Oleaceae	*Menodora*	*heterophylla*	Low menodora	Native	No	No	No	11
Oen.spe	Onagraceae	*Oenothera*	*speciosa*	Pinkladies	Native	No	No	No	1
Oxa.dic	Oxalidaceae	*Oxalis*	*dichondrifolia*	Peonyleaf woodsorrel	Native	No	No	No	2
Oxa.str	Oxalidaceae	*Oxalis*	*stricta*	Common yellow oxalis	Native	No	No	No	31
Pro.lou	Pedaliaceae	*Proboscidea*	*louisianica*	Ram's horn	Native	No	No	No	2
Pla.sp.	Plantaginaceae	*Plantago*	sp.	Plantain	Unknown	Unknown	No	No	1
Lim.car	Plumbaginaceae	*Limonium*	*carolinianum*	Lavender thrift	Native	No	No	No	5
Bot.isc	Poaceae	*Bothriochloa*	*ischaemum*	Yellow bluestem	Exotic	Yes	No	No	7
Cyn.dac	Poaceae	*Cynodon*	*dactylon*	Bermudagrass	Exotic	Yes	No	No	42
Dic.ann	Poaceae	*Dichanthium*	*annulatum*	Diaz bluestem	Exotic	Yes	No	No	2
Dig.san	Poaceae	*Digitaria*	*sanguinalis*	Hairy crabgrass	Exotic	Yes	No	No	6
Mon.lit	Poaceae	*Monanthochloe*	*littoralis*	Shoregrass	Native	No	No	No	63
Pen.cil	Poaceae	*Pennisetum*	*ciliare*	Buffelgrass	Exotic	Yes	No	No	47
Set.leu	Poaceae	*Setaria*	*leucopila*	Streambed bristlegrass	Native	No	No	No	4
Sor.bic	Poaceae	*Sorghum*	*bicolor*	grain sorghum	Exotic	No	No	No	58
Spa.spa	Poaceae	*Spartina*	*spartinae*	Gulf cordgrass	Native	No	No	No	21
Spo.cry	Poaceae	*Sporobolus*	*cryptandrus*	Spike dropseed	Native	No	No	No	1
Spo.vir	Poaceae	*Sporobolus*	*virginicus*	Seashore dropseed	Native	No	No	No	3
Spo.wri	Poaceae	*Sporobolus*	*wrightii*	Big sacaton	Native	No	No	No	1
Uro.max	Poaceae	*Urochloa*	*maxima*	Guineagrass	Exotic	Yes	No	No	84
Uro.pla	Poaceae	*Urochloa*	*platyphylla*	Broadleaf signalgrass	Native	Mixed	No	No	2
Por.ole	Portulacaceae	*Portulaca*	*oleracea*	Little hogweed	Exotic	Yes	No	No	1
Ana.arv	Primulaceae	*Anagallis*	*arvensis*	Scarlet pimpernel	Exotic	No	No	No	12
Con.hoo	Rhamnaceae	*Condalia*	*hookeri*	Brazilian bluewood	Native	No	Yes	No	5
Kar.hum	Rhamnaceae	*Karwinskia*	*humboldtiana*	Coyotillo	Native	No	Yes	No	2
Chi.alb	Rubiaceae	*Chiococca*	*alba*	West Indian milkberry	Native	No	Yes	No	1
Dio.rad	Rubiaceae	*Diodia*	*radula*	Rough buttonweed	Exotic	No	No	No	2
Ran.rha	Rubiaceae	*Randia*	*rhagocarpa*	Crucillo	Native	No	Yes	No	3
Ric.bra	Rubiaceae	*Richardia*	*brasiliensis*	Tropical Mexican clover	Exotic	No	No	No	62
Zan.fag	Rutaceae	*Zanthoxylum*	*fagara*	Lime pricklyash	Native	No	Yes	No	10
Sid.cel	Sapotaceae	*Sideroxylon*	*celastrinum*	Saffron plum	Native	No	Yes	Yes	2
Leu.fru	Scrophulariaceae	*Leucophyllum*	*frutescens*	Texas barometer bush	Native	No	Yes	Yes	5
Mec.pro	Scrophulariaceae	*Mecardonia*	*procumbens*	Baby jump-up	Native	No	No	No	1
Cas.ere	Simaroubaceae	*Castela*	*erecta*	Goatbush	Native	No	Yes	No	1
Smi.sp.	Smilacaceae	*Smilax*	sp.	Greenbriar	Native	No	No	No	2

Lyc.ber	Solanaceae	*Lycium*	*berlandieri*	Berlandier's wolfberry	Native	No	No	No	1
Lyc.car	Solanaceae	*Lycium*	*carolinianum*	Carolina desert-thorn	Native	No	No	Yes	8
Sol.ela	Solanaceae	*Solanum*	*elaeagnifolium*	Silverleaf nightshade	Native	No	No	No	6
Sol.sp.	Solanaceae	*Solanum*	sp.	Nightshade	Unknown	Unknown	No	No	2
Aye.lim	Sterculiaceae	*Ayenia*	*limitaris*	Rio Grande ayenia	Native	No	No	No	1
Cit.ber	Verbenaceae	*Citharexylum*	*berlandieri*	Berlandier fiddlewood	Native	No	Yes	No	3
Gla.bip	Verbenaceae	*Glandularia*	*bipinnatifida*	Dakota mock vervain	Native	No	No	No	7
Gla.pum	Verbenaceae	*Glandularia*	*pumila*	Pink mock vervain	Native	No	No	No	2
Lan.urt	Verbenaceae	*Lantana*	*urticoides*	Texas lantana	Native	No	No	Yes	13
Ver.hal	Verbenaceae	*Verbena*	*halei*	Texas vervain	Native	No	No	No	3
Ver.pli	Verbenaceae	*Verbena*	*plicata*	Fanleaf vervain	Native	No	No	No	1
Cis.tri	Vitaceae	*Cissus*	*trifoliata*	Sorrelvine	Native	No	No	No	6
Unk.dic	Dicot			Unknown dicot	Unknown	Unknown		No	23
Unk.mon	Monocot			Unknown monocot	Unknown	Unknown		No	9
Unknown	Unknown			Unknown	Unknown	Unknown		No	31

Table S1c. List of observed plant species, with the native and pest status, woody designation, whether it was observed to be in bloom during the survey period, and number of encounters for each taxa, ordered by encounters.

Code	Family (or group)	Genus	Species	Common name	Native Status	Pest Status	Woody	Obs. Bloom	Encounters
Bor.fru	Asteraceae	*Borrichia*	*frutescens*	Bushy seaside tansy	Native	No	No	No	108
Uro.max	Poaceae	*Urochloa*	*maxima*	Guineagrass	Exotic	Yes	No	No	84
Par.hys	Asteraceae	*Parthenium*	*hysterophorus*	Santa Maria feverfew	Uncertain	Yes	No	Yes	65
Mon.lit	Poaceae	*Monanthochloe*	*littoralis*	Shoregrass	Native	No	No	No	63
Ric.bra	Rubiaceae	*Richardia*	*brasiliensis*	Tropical Mexican clover	Exotic	No	No	No	62
Sor.bic	Poaceae	*Sorghum*	*bicolor*	grain sorghum	Exotic	No	No	No	58
Pen.cil	Poaceae	*Pennisetum*	*ciliare*	Buffelgrass	Exotic	Yes	No	No	47
Cyn.dac	Poaceae	*Cynodon*	*dactylon*	Bermudagrass	Exotic	Yes	No	No	42
Bat.mar	Bataceae	*Batis*	*maritima*	Turtleweed	Native	No	No	No	38
Pro.rep	Fabaceae	*Prosopis*	*reptans*	Tornillo	Native	No	No	No	38
Oxa.str	Oxalidaceae	*Oxalis*	*stricta*	Common yellow oxalis	Native	No	No	No	31
Pro.gla	Fabaceae	*Prosopis*	*glandulosa*	Honey mesquite	Native	Yes	Yes	No	31
Unknown	Unknown			Unknown	Unknown	Unknown		No	31
Sal.dep	Chenopodiaceae	*Salicornia*	*depressa*	Virginia glasswort	Native	No	No	No	30
Sua.lin	Chenopodiaceae	*Suaeda*	*linearis*	Annual seepweed	Native	No	No	No	23
Unk.dic	Dicot			Unknown dicot	Unknown	Unknown		No	23
Cyn.bar	Asclepiadaceae	*Cynanchum*	*barbigerum*	Bearded swallow-wort	Native	No	No	No	21
Spa.spa	Poaceae	*Spartina*	*spartinae*	Gulf cordgrass	Native	No	No	No	21
Cha.mac	Euphorbiaceae	*Chamaesyce*	*maculata*	Spotted sandmat	Native	No	No	No	17
Rhy.lat	Fabaceae	*Rhynchosia*	*latifolia*	Prairie snoutbean	Native	No	No	No	17
Cyc.lep	Apiaceae	*Cyclospermum*	*leptophyllum*	Marsh parsley	Exotic	No	No	No	16
Opu.eng	Cactaceae	*Opuntia*	*engelmannii*	Texas pricklypear	Native	No	No	Yes	16
May.phy	Celastraceae	*Maytenus*	*phyllanthoides*	Florida mayten	Native	No	Yes	No	15
Nep.pub	Fabaceae	*Neptunia*	*pubescens*	Tropical puff	Native	No	No	No	15
Mal.ame	Malvaceae	*Malvastrum*	*americanum*	Indian valley false mallow	Native	No	No	Yes	14
Lan.urt	Verbenaceae	*Lantana*	*urticoides*	Texas lantana	Native	No	No	Yes	13
Ana.arv	Primulaceae	*Anagallis*	*arvensis*	Scarlet pimpernel	Exotic	No	No	No	12
Cel.pal	Cannabaceae	*Celtis*	*pallida*	Granjeno	Native	No	Yes	No	12
Tar.off	Asteraceae	*Taraxacum*	*officinale*	Common dandelion	Exotic	Yes	No	No	12
Men.het	Oleaceae	*Menodora*	*heterophylla*	Low menodora	Native	No	No	No	11
Cyp.art	Cyperaceae	*Cyperus*	*articulatus*	Jointed flatsedge	Native	No	No	No	10
Ebe.eba	Fabaceae	*Ebenopsis*	*ebano*	Texas ebony	Native	No	Yes	No	10
Ray.ann	Asteraceae	*Rayjacksonia*	*annua*	Viscid tansyaster	Native	No	No	No	10

Teu.cub	Lamiaceae	*Teucrium*	*cubense*	Small coastal germander	Native	No	No	No	10
Zan.fag	Rutaceae	*Zanthoxylum*	*fagara*	Lime pricklyash	Native	No	Yes	No	10
Unk.mon	Monocot			Unknown monocot	Unknown	Unknown		No	9
Aca.tet	Cactaceae	*Acanthocereus*	*tetragonus*	Triangle cactus	Native	No	No	No	8
Cha.hum	Euphorbiaceae	*Chamaesyce*	*humistrata*	Spreading sandmat	Native	No	No	No	8
Ipo.hed	Convolvulaceae	*Ipomoea*	*hederacea*	Ivyleaf morningglory	Exotic	No	No	No	8
Lyc.car	Solanaceae	*Lycium*	*carolinianum*	Carolina desert-thorn	Native	No	No	Yes	8
Bot.isc	Poaceae	*Bothriochloa*	*ischaemum*	Yellow bluestem	Exotic	Yes	No	No	7
Chr.odo	Asteraceae	*Chromolaena*	*odorata*	Crucita blue mistflower	Native	No	No	Yes	7
Gla.bip	Verbenaceae	*Glandularia*	*bipinnatifida*	Dakota mock vervain	Native	No	No	No	7
Sid.acu	Malvaceae	*Sida*	*acuta*	Common wireweed	Native	No	No	No	7
Xan.str	Asteraceae	*Xanthium*	*strumarium*	Rough cocklebur	Native	Yes	No	No	7
Cis.tri	Vitaceae	*Cissus*	*trifoliata*	Sorrelvine	Native	No	No	No	6
Cyp.era	Cyperaceae	*Cyperus*	*eragrostis*	Tall Flatsedge	Native	No	No	No	6
Dig.san	Poaceae	*Digitaria*	*sanguinalis*	Hairy crabgrass	Exotic	Yes	No	No	6
Par.acu	Fabaceae	*Parkinsonia*	*aculeata*	Jerusalem thorn	Native	No	Yes	No	6
Rue.nud	Acanthaceae	*Ruellia*	*nudiflora*	Violet wild petunia	Native	No	No	No	6
Sol.ela	Solanaceae	*Solanum*	*elaeagnifolium*	Silverleaf nightshade	Native	No	No	No	6
Amm.coc	Lythraceae	*Ammannia*	*coccinea*	Valley redstem	Native	No	No	No	5
Con.hoo	Rhamnaceae	*Condalia*	*hookeri*	Brazilian bluewood	Native	No	Yes	No	5
Cyp.esc	Cyperaceae	*Cyperus*	*esculentus*	Yellow nutsedge	Exotic	No	No	No	5
For.ang	Oleaceae	*Forestiera*	*angustifolia*	Texas swampprivet	Native	No	Yes	No	5
Leu.fru	Scrophulariaceae	*Leucophyllum*	*frutescens*	Texas barometer bush	Native	No	Yes	Yes	5
Lim.car	Plumbaginaceae	*Limonium*	*carolinianum*	Lavender thrift	Native	No	No	No	5
Phy.pol	Euphorbiaceae	*Phyllanthus*	*polygonoides*	Smartweed leafflower	Native	No	No	No	5
Vac.far	Fabaceae	*Vachellia*	*farnesiana*	Sweet acacia	Native	Yes	Yes	No	5
Cor.sp.	Asteraceae	*Coreopsis*	sp.	Tickseed	Native	No	No	No	4
Flo.tri	Asteraceae	*Florestina*	*tripteris*	Sticky florestina	Native	No	No	No	4
Set.leu	Poaceae	*Setaria*	*leucopila*	Streambed bristlegrass	Native	No	No	No	4
Sta.dru	Lamiaceae	*Stachys*	*drummondii*	Drummond's hedgenettle	Native	No	No	No	4
Vig.ste	Asteraceae	*Viguiera*	*stenoloba*	Resinbush	Native	No	No	No	4
Asteraceae	Asteraceae			Aster family	Mixed	Mixed	No	No	4
Cen.asi	Apiaceae	*Centella*	*asiatica*	Spadeleaf	Exotic	No	No	No	3
Cit.ber	Verbenaceae	*Citharexylum*	*berlandieri*	Berlandier fiddlewood	Native	No	Yes	No	3
Ele.sp.	Cyperaceae	*Eleocharis*	sp.	Spikerush	Native	No	No	No	3
Kal.dai	Crassulaceae	*Kalanchoe*	*daigremontiana*	Devil's backbone	Exotic	Yes	No	No	3
Mar.ves	Marsileaceae	*Marsilea*	*vestita*	Hairy waterclover	Native	No	No	No	3

Opu.lep	Cactaceae	*Opuntia*	*leptocaulis*	Christmas cactus	Native	No	No	No	3
Ran.rha	Rubiaceae	*Randia*	*rhagocarpa*	Crucillo	Native	No	Yes	No	3
Rhy.ame	Fabaceae	*Rhynchosia*	*americana*	American snoutbean	Native	No	No	No	3
Sid.cor	Malvaceae	*Sida*	*cordifolia*	flannel weed	Native	No	No	No	3
Sid.rho	Malvaceae	*Sida*	*rhombifolia*	Cuban jute	Native	No	No	No	3
Spo.vir	Poaceae	*Sporobolus*	*virginicus*	Seashore dropseed	Native	No	No	No	3
Tam.azu	Asteraceae	*Tamaulipa*	*azurea*	Blue bonset	Native	No	No	No	3
Ver.hal	Verbenaceae	*Verbena*	*halei*	Texas vervain	Native	No	No	No	3
Vib.den	Caprifoliaceae	*Viburnum*	*dentatum*	Southern arrowwood	Native	No	Yes	No	3
Vig.lut	Fabaceae	*Vigna*	*luteola*	Hairypod cowpea	Native	No	No	No	3
Bac.neg	Asteraceae	*Baccharis*	*neglecta*	Rooseveltweed	Native	No	No	No	2
Bol.mar	Cyperaceae	*Bolboschoenus*	*maritimus*	Cosmopolitan bulrush	Native	No	No	No	2
Con.bet	Asteraceae	*Conoclinium*	*betonicifolium*	Mist flower	Native	No	No	No	2
Cro.sp.	Euphorbiaceae	*Croton*	sp.	Croton	Native	No	No	No	2
Dic.ann	Poaceae	*Dichanthium*	*annulatum*	Diaz bluestem	Exotic	Yes	No	No	2
Dio.rad	Rubiaceae	*Diodia*	*radula*	Rough buttonweed	Exotic	No	No	No	2
Ehr.ana	Boraginaceae	*Ehretia*	*anacua*	Knockaway	Native	No	Yes	No	2
Gla.pum	Verbenaceae	*Glandularia*	*pumila*	Pink mock vervain	Native	No	No	No	2
Gna.fal	Asteraceae	*Gnaphalium*	*falcatum*	narrowleaf purple everlasting	Native	No	No	No	2
Hel.ann	Asteraceae	*Helianthus*	*annuus*	Common sunflower	Native	No	No	Yes	2
Kar.hum	Rhamnaceae	*Karwinskia*	*humboldtiana*	Coyotillo	Native	No	Yes	No	2
Lyt.ala	Lythraceae	*Lythrum*	*alatum*	Winged lythrum	Native	No	No	No	2
Lyt.cal	Lythraceae	*Lythrum*	*californicum*	California loosestrife	Native	No	No	No	2
Mal.par	Malvaceae	*Malva*	*parviflora*	Cheeseweed mallow	Exotic	No	No	No	2
Oxa.dic	Oxalidaceae	*Oxalis*	*dichondrifolia*	Peonyleaf woodsorrel	Native	No	No	No	2
Pro.lou	Pedaliaceae	*Proboscidea*	*louisianica*	Ram's horn	Native	No	No	No	2
Rhy.tex	Fabaceae	*Rhynchosia*	*texana*	Texas snoutbean	Native	No	No	No	2
Rhy.col	Cyperaceae	*Rhynchospora*	*colorata*	Starrush whitetop	Native	No	No	No	2
Rue.sp.	Acanthaceae	*Ruellia*	sp.	Wild petunia	Unknown	Unknown	No	No	2
Sal.bal	Lamiaceae	*Salvia*	*ballotaeflora*	Shrubby blue sage	Native	No	No	No	2
Sch.cun	Celastraceae	*Schaefferia*	*cuneifolia*	Desert yaupon	Native	No	Yes	No	2
Scu.ocm	Lamiaceae	*Scutellaria*	*ocmulgee*	Ocmulgee skullcap	Exotic	No	No	No	2
Ses.her	Fabaceae	*Sesbania*	*herbacea*	Bigpod sesbania	Native	No	Yes	No	2
Sid.sp.	Malvaceae	*Sida*	sp.	Fanpetal	Native	No	No	No	2
Sid.cel	Sapotaceae	*Sideroxylon*	*celastrinum*	Saffron plum	Native	No	Yes	Yes	2
Smi.sp.	Smilacaceae	*Smilax*	sp.	Greenbriar	Native	No	No	No	2
Sol.sp.	Solanaceae	*Solanum*	sp.	Nightshade	Unknown	Unknown	No	No	2

Uro.pla	Poaceae	*Urochloa*	*platyphylla*	Broadleaf signalgrass	Native	Mixed	No	No	2
Ama.pol	Amaranthaceae	*Amaranthus*	*polygonoides*	Tropical amaranth	Native	No	No	No	1
Aph.ski	Asteraceae	*Aphanostephus*	*skirrhobasis*	Arkansas dozedaisy	Native	No	No	No	1
Asc.asp	Asclepiadaceae	*Asclepias*	*asperula*	Spider milkweed	Native	No	No	No	1
Ast.nut	Fabaceae	*Astragalus*	*nuttallianus*	Smallflower milkvetch	Native	No	No	No	1
Aye.lim	Sterculiaceae	*Ayenia*	*limitaris*	Rio Grande ayenia	Native	No	No	No	1
Bud.ses	Buddlejaceae	*Buddleja*	*sessiliflora*	Rio Grande butterflybush	Native	No	No	No	1
Cas.ere	Simaroubaceae	*Castela*	*erecta*	Goatbush	Native	No	Yes	No	1
Cha.cor	Euphorbiaceae	*Chamaesyce*	*cordifolia*	Heartleaf sandmat	Native	No	No	No	1
Cha.gly	Euphorbiaceae	*Chamaesyce*	*glyptosperma*	Ribseed sandmat	Native	No	No	No	1
Chi.alb	Rubiaceae	*Chiococca*	*alba*	West Indian milkberry	Native	No	Yes	No	1
Cic.sp.	Apiaceae	*Cicuta*	sp.	Water hemlock	Native	No	No	No	1
Cla.sua	Asteraceae	*Clappia*	*suaedifolia*	Fleshy clapdaisy	Native	No	No	No	1
Con.coe	Asteraceae	*Conoclinium*	*coelestinum*	Blue mistflower	Native	No	No	No	1
Con.ram	Asteraceae	*Conyza*	*ramosissima*	Dwarf horseweed	Native	No	No	No	1
Cre.nud	Convolvulaceae	*Cressa*	*nudicaulis*	Nakedstem alkaliweed	Native	No	No	No	1
Cro.cap	Euphorbiaceae	*Croton*	*capitatus*	Lindheimer's hogwort	Native	No	No	No	1
Cro.hum	Euphorbiaceae	*Croton*	*humilis*	Pepperbush	Native	No	No	No	1
Evo.als	Convolvulaceae	*Evolvulus*	*alsinoides*	Slender dwarf morning glory	Native	No	No	No	1
Fim.cas	Cyperaceae	*Fimbristylis*	*castanea*	Marsh fimbry	Native	No	No	No	1
Gna.pen	Asteraceae	*Gamochaeta*	*pensylvanica*	Pennsylvania everlasting	Native	No	No	No	1
Gom.nea	Amaranthaceae	*Gomphrena*	*nealleyi*	Nealley's globe amaranth	Native	No	No	No	1
Hei.sal	Lythraceae	*Heimia*	*salicifolia*	Shrubby yellowcrest	Native	No	No	No	1
Ibe.lin	Cucurbitaceae	*Ibervillea*	*lindheimeri*	Lindheimer's globeberry	Native	No	No	No	1
Ibe.ten	Cucurbitaceae	*Ibervillea*	*tenuisecta*	Slimlobe globeberry	Native	No	No	No	1
Ipo.cor	Convolvulaceae	*Ipomoea*	*cordatotriloba*	Tievine	Native	No	No	No	1
Lyc.ber	Solanaceae	*Lycium*	*berlandieri*	Berlandier's wolfberry	Native	No	No	No	1
Mec.pro	Scrophulariaceae	*Mecardonia*	*procumbens*	Baby jump-up	Native	No	No	No	1
Mol.ver	Molluginaceae	*Mollugo*	*verticillata*	green carpetweed	Native	No	No	No	1
Mon.fru	Lamiaceae	*Monarda*	*fruticulosa*	Spotted beebalm	Native	No	No	No	1
Nam.par	Boraginaceae	*Nama*	*parvifolia*	Small-leaf fiddleleaf	Native	No	No	No	1
Oen.spe	Onagraceae	*Oenothera*	*speciosa*	Pinkladies	Native	No	No	No	1
Par.con	Asteraceae	*Parthenium*	*confertum*	Gray's feverfew	Native	No	No	No	1
Pla.sp.	Plantaginaceae	*Plantago*	sp.	Plantain	Unknown	Unknown	No	No	1
Por.ole	Portulacaceae	*Portulaca*	*oleracea*	Little hogweed	Exotic	Yes	No	No	1
Rud.hir	Asteraceae	*Rudbeckia*	*hirta*	Blackeyed Susan	Native	No	No	No	1
Rue.cae	Acanthaceae	*Ruellia*	*caerulea*	Britton's wild petunia	Exotic	Yes	No	No	1

Rue.run	Acanthaceae	*Ruellia*	*nudiflora* var. *runyonii*	Runyon's wild petunia	Native	No	No	No	1
Ses.por	Aizoaceae	*Sesuvium*	*portulacastrum*	Shoreline seapurslane	Native	No	No	No	1
Ses.ses	Aizoaceae	*Sesuvium*	*sessile*	Western seapurslane	Native	No	No	No	1
Sid.lin	Malvaceae	*Sida*	*lindheimeri*	Showy fanpetals	Native	No	No	No	1
Son.ole	Asteraceae	*Sonchus*	*oleraceus*	Common sowthistle	Exotic	No	No	No	1
Son.sp.	Asteraceae	*Sonchus*	sp.	Sowthistle	Unknown	Unknown	No	No	1
Sph.sp.	Malvaceae	*Sphaeralcea*	sp.	Globemallow	Native	No	No	No	1
Spo.cry	Poaceae	*Sporobolus*	*cryptandrus*	Spike dropseed	Native	No	No	No	1
Spo.wri	Poaceae	*Sporobolus*	*wrightii*	Big sacaton	Native	No	No	No	1
Teu.sp.	Lamiaceae	*Teucrium*	sp.	Germander	Native	No	No	No	1
Ver.pli	Verbenaceae	*Verbena*	*plicata*	Fanleaf vervain	Native	No	No	No	1
Xan.sp.	Asteraceae	*Xanthocephalum*	sp.	Yellow head aster	Native	No	No	No	1
Yuc.tre	Agavaceae	*Yucca*	*treculeana*	Don Quixote's lace	Native	No	Yes	No	1
Caryophyllaceae	Caryophyllaceae			Carnation family	Unknown	Unknown	No	No	1
Fabaceae	Fabaceae			Legume family	Mixed	Mixed	Mixed	No	1
