# Supplementary material for: Effects of Patch Size, Fragmentation, and Invasive Species on Plant and Lepidoptera Communities in Southern Texas
Source: Insects. 2021 Aug 29;12(9):777. doi: 10.3390/insects12090777 (PMC8472066; doi:10.3390/insects12090777)
Supplement: Supplementary file 1 [file insects-12-00777-s001.zip › Table S2.pdf]

Table S2a. List of observed lepidoptera species and the number of times each was encountered, ordered by species.

Code	Family	Subfamily	Genus	Species	Common name	Encounters
Ach.alb	Hesperiidae	Eudaminae	*Achalarus*	*albociliatus*	Skinner's cloudywing	1
Ach.ran	Crambidae	Pyraustinae	*Achyra*	*rantalis*	Garden webworm	1
Agr.van	Nymphalidae	Heliconiinae	*Agraulis*	*vanillae*	Gulf fritillary	1
Amb.cel	Hesperiidae	Hesperiinae	*Amblyscirtes*	*celia*	Celia's roadside-skipper	2
Amo.ros	Erebidae	Erebinae	*Amolita*	*roseola*	Owlet moth	1
Amy.bul	Noctuidae	Bagisarinae	*Amyna*	*bullula*	Hook-tipped amyna	2
Ana.jat	Nymphalidae	Nymphalinae	*Anartia*	*jatrophae*	White peacock	4
Ani.ill	Noctuidae	Noctuinae	*Anicla*	*illapsa*	Snowy dart	1
Ani.sim	Noctuidae	Noctuinae	*Anicla*	*simplicius*	Simplicius dart moth	1
Ano.pri	Erebidae	Scoliopteryginae	*Anomis*	*privata*	Hibiscus-leaf caterpillar moth	1
Pyr.ara	Hesperiidae	Pyrginae	*Apyrrothrix*	*araxes*	Dull firetip	1
Asc.mon	Pieridae	Pierinae	*Ascia*	*monuste*	Great southern white	6
Bag.rep	Noctuidae	Bagisarinae	*Bagisara*	*repanda*	Wavy lined mallow moth	2
Bat.phi	Papilionidae	Papilioninae	*Battus*	*philenor*	Pipevine swallowtail	1
Ble.san	Erebidae	Herminiinae	*Bleptina*	*sangamonia*	Owlet moth	1
Bre.exi	Lycaenidae	Polyommatinae	*Brephidium*	*exilis*	Western pygmy-blue	2
Cal.eth	Hesperiidae	Hesperiinae	*Calpodes*	*ethlius*	Brazilian skipper	1
Cat.lin	Noctuidae	Oncocnemidinae	*Catabena*	*lineolata*	Five-lined sallow	1
Cat.ala	Erebidae	Erebinae	*Catocala*	*alabamae*	Alabama underwing	1
Cen.pet	Tortricidae	Tortricinae	*Cenopis*	*pettitana*	Maple-basswood leafroller moth	1
Cis.plu	Erebidae	Arctiinae	*Cisthene*	*plumbea*	Lead colored lichen moth	1
Cna.coc	Crambidae	Spilomelinae	*Cnaphalocrocis*	*cochrusalis*	Marasmia moth	1
Cop.min	Hesperiidae	Hesperiinae	*Copaeodes*	*minima*	Southern skipperling	1
Cym.odi	Hesperiidae	Hesperiinae	*Cymaenes*	*trebius*	Fawn-spotted skipper	2
Dan.gil	Nymphalidae	Danainae	*Danaus*	*gilippus*	Queen butterfly	13
Dan.ple	Nymphalidae	Danainae	*Danaus*	*plexippus*	Monarch butterfly	3
Dec.per	Hesperiidae	Hesperiinae	*Decinea*	*percosius*	Double-dotted Skipper	1
Don.mel	Crambidae	Schoenobiinae	*Donacaula*	*melinellus*		1
Don.sor	Crambidae	Schoenobiinae	*Donacaula*	*sordidellus*		1
Dry.iul	Nymphalidae	Heliconiinae	*Dryas*	*iulia*	Julia Heliconian	3
Dys.pun	Erebidae	Hypenodinae	*Dyspyralis*	*puncticosta*	Spot-edged Dyspyralis moth	1
Ean.thr	Hesperiidae	Pyrginae	*Eantis*	*thraso*	Sickle-winged skipper	2
Ech.iso	Lycaenidae	Polyommatinae	*Echinargus*	*isola*	Reakirts blue	4

Ela.fus	Noctuidae	Noctuinae	*Elaphria*	*fuscimacula*	Cutworm	1
Ela.nuc	Noctuidae	Noctuinae	*Elaphria*	*nucicolora*	Sugarcane midget	1
Ela.spp	Noctuidae	Noctuinae	*Elaphria*	sp.	Midgets	2
Eor.den	Crambidae	Crambinae	*Eoreuma*	*densella*	Wainscot grass-veneer	1
Ery.fun	Hesperiidae	Pyrginae	*Erynnis*	*funeralis*	Funereal duskywing	1
Eup.ves	Hesperiidae	Hesperiinae	*Euphyes*	*vestris*	Dun skipper	2
Eup.cla	Nymphalidae	Heliconiinae	*Euptoieta*	*claudia*	Variegated fritillary	1
Fru.sp.	Gelechiidae	Gelechiinae	*Frumenta*	sp.	Twirler moth	1
Hel.lav	Hesperiidae	Pyrginae	*Heliopetes*	*laviana*	Laviana white-skipper	3
Hel.mac	Hesperiidae	Pyrginae	*Heliopetes*	*macaira*	Turks-cap white skipper	1
Hem.sco	Erebidae	Boletobiinae	*Hemeroplanis*	*scopulepes*	Variable tropic	2
Hem.cer	Lycaenidae	Polyommatinae	*Hemiargus*	*ceraunus*	Ceraunus blue	5
Her.sos	Nymphalidae	Satyrinae	*Hermeuptychia*	*sosybius*	Carolina satyr	1
Her.aeg	Crambidae	Spilomelinae	*Herpetogramma*	*aeglealis*	Serpentine webworm	1
Her.bip	Crambidae	Spilomelinae	*Herpetogramma*	*bipunctalis*	Southern beet webworm moth	4
Her.flu	Crambidae	Spilomelinae	*Herpetogramma*	*fluctuosalis*	Greater sweet potato webworm moth	1
Her.sp.	Crambidae	Spilomelinae	*Herpetogramma*	sp.	Webworm moth	2
Hom.ele	Pyralidae	Phycitinae	*Homeosoma*	*electella*	Sunflower moth	1
Hym.per	Crambidae	Spilomelinae	*Hymenia*	*perspectalis*	Spotted beet webworm moth	5
Hyp.min	Erebidae	Hypeninae	*Hypena*	*minualis*	Sooty bomolocha moth	1
Ida.obf	Geometridae	Sterrhinae	*Idaea*	*obfusaria*	Rippled wave	1
Iso.ten	Erebidae	Boletobiinae	*Isogona*	*tenuis*	Thin-lined owlet	1
Kri.lys	Pieridae	Coliadinae	*Kricogonia*	*lyside*	Lyside sulphur	2
Lac.sub	Lacturidae		*Lactura*	*subfervens*	Speckled lactura	1
Ler.acc	Hesperiidae	Hesperiinae	*Lerema*	*accius*	Clouded skipper	3
Ler.euf	Hesperiidae	Hesperiinae	*Lerodea*	*eufala*	Eufala skipper	2
Leu.inc	Noctuidae	Noctuinae	*Leucania*	*incognita*	Wainscot moth	1
Lib.car	Nymphalidae	Libytheinae	*Libytheana*	*carinenta*	American snout	18
Lob.per	Geometridae	Sterrhinae	*Lobocleta*	*peralbata*		1
Mac.bic	Geometridae	Ennominae	*Macaria*	*bicolorata*	Bicolored angle	1
Mel.ind	Erebidae	Erebinae	*Melipotis*	*indomita*	Indomitable melipotis	2
Mes.amy	Nymphalidae	Biblidinae	*Mestra*	*amymone*	Common mestra	2
Mim.ruf	Crambidae	Odontiinae	*Mimoschinia*	*rufofascialis*	Rufous-banded crambid moth	1
Moc.dis	Erebidae	Erebinae	*Mocis*	*disseverans*	Yellow mocis	3
Moc.lat	Erebidae	Erebinae	*Mocis*	*latipes*	Small mocis	12

Moc.mar	Erebidae	Erebinae	*Mocis*	*marcida*	Withered mocis	12
Moc.tex	Erebidae	Erebinae	*Mocis*	*texana*	Texas mocis	2
Nat.iol	Papilionidae	Pierinae	*Nathalis*	*iole*	Dainty sulphur	1
Noc.pro	Noctuidae	Noctuinae	*Noctua*	*pronuba*	European yellow underwing	1
Nom.nea	Psychodomorpha	Psychodidae	*Nomophila*	*nearctica*	American celery webworm	1
Nyc.nyc	Hesperiidae	Hesperiinae	*Nyctelius*	*nyctelius*	Violet-banded skipper	3
Pal.fle	Crambidae	Pyraustinae	*Palpita*	*flegia*	Satin white palpita moth	1
Pal.qua	Crambidae	Spilomelinae	*Palpita*	*quadristigmalis*	Four-spotted palpita moth	1
Pan.rep	Erebidae	Eulepidotinae	*Panopoda*	*repanda*	Orange panopoda	1
Pan.oco	Hesperiidae	Hesperiinae	*Panoquina*	*ocola*	Ocola skipper	2
Pan.pan	Hesperiidae	Hesperiinae	*Panoquina*	*panoquinoides*	Obscure skipper	5
Pap.pol	Papilionidae	Papilioninae	*Papilio*	*polyxenes*	Black swallowtail	3
Pho.aga	Pieridae	Coliadinae	*Phoebis*	*agarithe*	Large orange sulphur	1
Pho.phi	Pieridae	Coliadinae	*Phoebis*	*philea*	Orange-barred sulphur	1
Pho.sen	Pieridae	Coliadinae	*Phoebis*	*sennae*	Cloudless sulphur	3
Phy.pha	Nymphalidae	Nymphalinae	*Phyciodes*	*phaon*	Phaon crescent	5
Pol.fla	Crambidae	Pyraustinae	*Polygrammodes*	*flavidalis*	Ironweed root moth	1
Pon.pro	Pieridae	Pierinae	*Pontia*	*protodice*	Checkered white	1
Psa.aby	Geometridae	Ennominae	*Psamatodes*	*abydata*	Dot-lined angle	1
Pse.san	Crambidae	Pyraustinae	*Pseudopyrausta*	*santatalis*	Crambid snout moth	1
Pyr.sp.	Crambidae	Pyraustinae	*Pyrausta*	sp.	Crambid snout moths	1
Pyr.alb	Hesperiidae	Pyrginae	*Pyrgus*	*albescens*	White checkered-skipper	4
Pyr.com	Hesperiidae	Pyrginae	*Pyrgus*	*communis*	Common checkered-skipper	1
Pyr.lis	Pieridae	Coliadinae	*Pyrisitia*	*lisa*	Little yellow	18
Pyr.pro	Pieridae	Coliadinae	*Pyrisitia*	*proterpia*	Tailed orange	1
Ren.sp.	Erebidae	Herminiinae	*Renia*	sp.	Erebid moths	1
Rin.sig	Ennominae	Geometridae	*Rindgea*	*s-signata*	Signate looper moth	1
Rin.cyd	Ennominae	Geometridae	*Ringdea*	*cyda*	Mesquite looper moth	3
Sch.sp.	Erebidae	Hypenodinae	*Schrankia*	sp.	Schrankia	1
Sco.lim	Geometridae	Sterrhinae	*Scopula*	*limboundata*	Large lace-border	1
Sco.umb	Geometridae	Sterrhinae	*Scopula*	*umbilicata*	Swag-lined wave	1
Spo.rec	Crambidae	Pyraustinae	*Spoladea*	*recurvalis*	Hawaiian beet webworm	6
Str.ist	Lycaenidae	Theclinae	*Strymon*	*istapa*	Mallow Scrub-Hairstreak	1
Str.mel	Lycaenidae	Theclinae	*Strymon*	*melinus*	Gray hairstreak	1
Van.ata	Nymphalidae	Nymphalinae	*Vanessa*	*atalanta*	Red admiral	2

Van.car	Nymphalidae	Nymphalinae	*Vanessa*	*cardui*	Painted lady	1
Van.vir	Nymphalidae	Nymphalinae	*Vanessa*	*virginiensis*	American painted lady	2
Vid.per	Hesperiidae	Hesperiinae	*Vidius*	*perigenes*	Pale-rayed Skipper	1
Vir.sp.	Erebidae	Arctiinae	*Virbia*	sp.	Virbia	1
Zer.ces	Pieridae	Coliadinae	*Zerene*	*cesonia*	Southern dogface	7
Lymantriinae	Erebidae	Lymantriinae			Tussock moth	1
Erebidae	Erebidae				Erebid moths	1
Scythrididae	Scythrididae				Flower moths	1
Unknown	Unknown				Unknown	10

Table S2b. List of observed lepidoptera species and the number of times each was encountered, ordered by family and subfamily.

Code	Family	Subfamily	Genus	Species	Common name	Encounters
Eor.den	Crambidae	Crambinae	*Eoreuma*	*densella*	Wainscot grass-veneer	1
Mim.ruf	Crambidae	Odontiinae	*Mimoschinia*	*rufofascialis*	Rufous-banded crambid moth	1
Ach.ran	Crambidae	Pyraustinae	*Achyra*	*rantalis*	Garden webworm	1
Pal.fle	Crambidae	Pyraustinae	*Palpita*	*flegia*	Satin white palpita moth	1
Pol.fla	Crambidae	Pyraustinae	*Polygrammodes*	*flavidalis*	Ironweed root moth	1
Pse.san	Crambidae	Pyraustinae	*Pseudopyrausta*	*santatalis*	Crambid snout moth	1
Pyr.sp.	Crambidae	Pyraustinae	*Pyrausta*	sp.	Crambid snout moths	1
Spo.rec	Crambidae	Pyraustinae	*Spoladea*	*recurvalis*	Hawaiian beet webworm	6
Don.mel	Crambidae	Schoenobiinae	*Donacaula*	*melinellus*		1
Don.sor	Crambidae	Schoenobiinae	*Donacaula*	*sordidellus*		1
Cna.coc	Crambidae	Spilomelinae	*Cnaphalocrocis*	*cochrusalis*	Marasmia moth	1
Her.aeg	Crambidae	Spilomelinae	*Herpetogramma*	*aeglealis*	Serpentine webworm	1
Her.bip	Crambidae	Spilomelinae	*Herpetogramma*	*bipunctalis*	Southern beet webworm moth	4
Her.flu	Crambidae	Spilomelinae	*Herpetogramma*	*fluctuosalis*	Greater sweet potato webworm moth	1
Her.sp.	Crambidae	Spilomelinae	*Herpetogramma*	sp.	Webworm moth	2
Hym.per	Crambidae	Spilomelinae	*Hymenia*	*perspectalis*	Spotted beet webworm moth	5
Pal.qua	Crambidae	Spilomelinae	*Palpita*	*quadristigmalis*	Four-spotted palpita moth	1
Rin.sig	Ennominae	Geometridae	*Rindgea*	*s-signata*	Signate looper moth	1
Rin.cyd	Ennominae	Geometridae	*Ringdea*	*cyda*	Mesquite looper moth	3
Sch.sp.	Erebidae	Hypenodinae	*Schrankia*	sp.	Schrankia	1
Cis.plu	Erebidae	Arctiinae	*Cisthene*	*plumbea*	Lead colored lichen moth	1
Vir.sp.	Erebidae	Arctiinae	*Virbia*	sp.	Virbia	1
Hem.sco	Erebidae	Boletobiinae	*Hemeroplanis*	*scopulepes*	Variable tropic	2
Iso.ten	Erebidae	Boletobiinae	*Isogona*	*tenuis*	Thin-lined owlet	1
Amo.ros	Erebidae	Erebinae	*Amolita*	*roseola*	Owlet moth	1
Cat.ala	Erebidae	Erebinae	*Catocala*	*alabamae*	Alabama underwing	1
Mel.ind	Erebidae	Erebinae	*Melipotis*	*indomita*	Indomitable melipotis	2
Moc.dis	Erebidae	Erebinae	*Mocis*	*disseverans*	Yellow mocis	3
Moc.lat	Erebidae	Erebinae	*Mocis*	*latipes*	Small mocis	12
Moc.mar	Erebidae	Erebinae	*Mocis*	*marcida*	Withered mocis	12
Moc.tex	Erebidae	Erebinae	*Mocis*	*texana*	Texas mocis	2
Pan.rep	Erebidae	Eulepidotinae	*Panopoda*	*repanda*	Orange panopoda	1
Ble.san	Erebidae	Herminiinae	*Bleptina*	*sangamonia*	Owlet moth	1

Ren.sp.	Erebidae	Herminiinae	*Renia*	sp.	Erebid moths	1
Hyp.min	Erebidae	Hypeninae	*Hypena*	*minualis*	Sooty bomolocha moth	1
Dys.pun	Erebidae	Hypenodinae	*Dyspyralis*	*puncticosta*	Spot-edged Dyspyralis moth	1
Lymantriinae	Erebidae	Lymantriinae			Tussock moth	1
Ano.pri	Erebidae	Scoliopteryginae	*Anomis*	*privata*	Hibiscus-leaf caterpillar moth	1
Erebidae	Erebidae				Erebid moths	1
Fru.sp.	Gelechiidae	Gelechiinae	*Frumenta*	sp.	Twirler moth	1
Mac.bic	Geometridae	Ennominae	*Macaria*	*bicolorata*	Bicolored angle	1
Psa.aby	Geometridae	Ennominae	*Psamatodes*	*abydata*	Dot-lined angle	1
Ida.obf	Geometridae	Sterrhinae	*Idaea*	*obfusaria*	Rippled wave	1
Lob.per	Geometridae	Sterrhinae	*Lobocleta*	*peralbata*		1
Sco.lim	Geometridae	Sterrhinae	*Scopula*	*limboundata*	Large lace-border	1
Sco.umb	Geometridae	Sterrhinae	*Scopula*	*umbilicata*	Swag-lined wave	1
Ach.alb	Hesperiidae	Eudaminae	*Achalarus*	*albociliatus*	Skinner's cloudywing	1
Amb.cel	Hesperiidae	Hesperiinae	*Amblyscirtes*	*celia*	Celia's roadside-skipper	2
Cal.eth	Hesperiidae	Hesperiinae	*Calpodes*	*ethlius*	Brazilian skipper	1
Cop.min	Hesperiidae	Hesperiinae	*Copaeodes*	*minima*	Southern skipperling	1
Cym.odi	Hesperiidae	Hesperiinae	*Cymaenes*	*trebius*	Fawn-spotted skipper	2
Dec.per	Hesperiidae	Hesperiinae	*Decinea*	*percosius*	Double-dotted Skipper	1
Eup.ves	Hesperiidae	Hesperiinae	*Euphyes*	*vestris*	Dun skipper	2
Ler.acc	Hesperiidae	Hesperiinae	*Lerema*	*accius*	Clouded skipper	3
Ler.euf	Hesperiidae	Hesperiinae	*Lerodea*	*eufala*	Eufala skipper	2
Nyc.nyc	Hesperiidae	Hesperiinae	*Nyctelius*	*nyctelius*	Violet-banded skipper	3
Pan.oco	Hesperiidae	Hesperiinae	*Panoquina*	*ocola*	Ocola skipper	2
Pan.pan	Hesperiidae	Hesperiinae	*Panoquina*	*panoquinoides*	Obscure skipper	5
Vid.per	Hesperiidae	Hesperiinae	*Vidius*	*perigenes*	Pale-rayed Skipper	1
Pyr.ara	Hesperiidae	Pyrginae	*Apyrrothrix*	*araxes*	Dull firetip	1
Ean.thr	Hesperiidae	Pyrginae	*Eantis*	*thraso*	Sickle-winged skipper	2
Ery.fun	Hesperiidae	Pyrginae	*Erynnis*	*funeralis*	Funereal duskywing	1
Hel.lav	Hesperiidae	Pyrginae	*Heliopetes*	*laviana*	Laviana white-skipper	3
Hel.mac	Hesperiidae	Pyrginae	*Heliopetes*	*macaira*	Turks-cap white skipper	1
Pyr.alb	Hesperiidae	Pyrginae	*Pyrgus*	*albescens*	White checkered-skipper	4
Pyr.com	Hesperiidae	Pyrginae	*Pyrgus*	*communis*	Common checkered-skipper	1
Lac.sub	Lacturidae		*Lactura*	*subfervens*	Speckled lactura	1
Bre.exi	Lycaenidae	Polyommatinae	*Brephidium*	*exilis*	Western pygmy-blue	2

Ech.iso	Lycaenidae	Polyommatinae	*Echinargus*	*isola*	Reakirts blue	4
Hem.cer	Lycaenidae	Polyommatinae	*Hemiargus*	*ceraunus*	Ceraunus blue	5
Str.ist	Lycaenidae	Theclinae	*Strymon*	*istapa*	Mallow Scrub-Hairstreak	1
Str.mel	Lycaenidae	Theclinae	*Strymon*	*melinus*	Gray hairstreak	1
Amy.bul	Noctuidae	Bagisarinae	*Amyna*	*bullula*	Hook-tipped amyna	2
Bag.rep	Noctuidae	Bagisarinae	*Bagisara*	*repanda*	Wavy lined mallow moth	2
Ani.ill	Noctuidae	Noctuinae	*Anicla*	*illapsa*	Snowy dart	1
Ani.sim	Noctuidae	Noctuinae	*Anicla*	*simplicius*	Simplicius dart moth	1
Ela.fus	Noctuidae	Noctuinae	*Elaphria*	*fuscimacula*	Cutworm	1
Ela.nuc	Noctuidae	Noctuinae	*Elaphria*	*nucicolora*	Sugarcane midget	1
Ela.spp	Noctuidae	Noctuinae	*Elaphria*	sp.	Midgets	2
Leu.inc	Noctuidae	Noctuinae	*Leucania*	*incognita*	Wainscot moth	1
Noc.pro	Noctuidae	Noctuinae	*Noctua*	*pronuba*	European yellow underwing	1
Cat.lin	Noctuidae	Oncocnemidinae	*Catabena*	*lineolata*	Five-lined sallow	1
Mes.amy	Nymphalidae	Biblidinae	*Mestra*	*amymone*	Common mestra	2
Dan.gil	Nymphalidae	Danainae	*Danaus*	*gilippus*	Queen butterfly	13
Dan.ple	Nymphalidae	Danainae	*Danaus*	*plexippus*	Monarch butterfly	3
Agr.van	Nymphalidae	Heliconiinae	*Agraulis*	*vanillae*	Gulf fritillary	1
Dry.iul	Nymphalidae	Heliconiinae	*Dryas*	*iulia*	Julia Heliconian	3
Eup.cla	Nymphalidae	Heliconiinae	*Euptoieta*	*claudia*	Variegated fritillary	1
Lib.car	Nymphalidae	Libytheinae	*Libytheana*	*carinenta*	American snout	18
Ana.jat	Nymphalidae	Nymphalinae	*Anartia*	*jatrophae*	White peacock	4
Phy.pha	Nymphalidae	Nymphalinae	*Phyciodes*	*phaon*	Phaon crescent	5
Van.ata	Nymphalidae	Nymphalinae	*Vanessa*	*atalanta*	Red admiral	2
Van.car	Nymphalidae	Nymphalinae	*Vanessa*	*cardui*	Painted lady	1
Van.vir	Nymphalidae	Nymphalinae	*Vanessa*	*virginiensis*	American painted lady	2
Her.sos	Nymphalidae	Satyrinae	*Hermeuptychia*	*sosybius*	Carolina satyr	1
Bat.phi	Papilionidae	Papilioninae	*Battus*	*philenor*	Pipevine swallowtail	1
Pap.pol	Papilionidae	Papilioninae	*Papilio*	*polyxenes*	Black swallowtail	3
Nat.iol	Papilionidae	Pierinae	*Nathalis*	*iole*	Dainty sulphur	1
Kri.lys	Pieridae	Coliadinae	*Kricogonia*	*lyside*	Lyside sulphur	2
Pho.aga	Pieridae	Coliadinae	*Phoebis*	*agarithe*	Large orange sulphur	1
Pho.phi	Pieridae	Coliadinae	*Phoebis*	*philea*	Orange-barred sulphur	1
Pho.sen	Pieridae	Coliadinae	*Phoebis*	*sennae*	Cloudless sulphur	3
Pyr.lis	Pieridae	Coliadinae	*Pyrisitia*	*lisa*	Little yellow	18

Pyr.pro	Pieridae	Coliadinae	*Pyrisitia*	*proterpia*	Tailed orange	1
Zer.ces	Pieridae	Coliadinae	*Zerene*	*cesonia*	Southern dogface	7
Asc.mon	Pieridae	Pierinae	*Ascia*	*monuste*	Great southern white	6
Pon.pro	Pieridae	Pierinae	*Pontia*	*protodice*	Checkered white	1
Nom.nea	Psychodomorpha	Psychodidae	*Nomophila*	*nearctica*	American celery webworm	1
Hom.ele	Pyralidae	Phycitinae	*Homeosoma*	*electella*	Sunflower moth	1
Scythrididae	Scythrididae				Flower moths	1
Cen.pet	Tortricidae	Tortricinae	*Cenopis*	*pettitana*	Maple-basswood leafroller moth	1
Unknown	Unknown				Unknown	10

Table S2c. List of observed lepidoptera species and the number of times each was encountered, ordered by encounters.

Code	Family	Subfamily	Genus	Species	Common name	Encounters
Lib.car	Nymphalidae	Libytheinae	*Libytheana*	*carinenta*	American snout	18
Pyr.lis	Pieridae	Coliadinae	*Pyrisitia*	*lisa*	Little yellow	18
Dan.gil	Nymphalidae	Danainae	*Danaus*	*gilippus*	Queen butterfly	13
Moc.lat	Erebidae	Erebinae	*Mocis*	*latipes*	Small mocis	12
Moc.mar	Erebidae	Erebinae	*Mocis*	*marcida*	Withered mocis	12
Unknown	Unknown				Unknown	10
Zer.ces	Pieridae	Coliadinae	*Zerene*	*cesonia*	Southern dogface	7
Asc.mon	Pieridae	Pierinae	*Ascia*	*monuste*	Great southern white	6
Spo.rec	Crambidae	Pyraustinae	*Spoladea*	*recurvalis*	Hawaiian beet webworm	6
Hem.cer	Lycaenidae	Polyommatinae	*Hemiargus*	*ceraunus*	Ceraunus blue	5
Hym.per	Crambidae	Spilomelinae	*Hymenia*	*perspectalis*	Spotted beet webworm moth	5
Pan.pan	Hesperiidae	Hesperiinae	*Panoquina*	*panoquinoides*	Obscure skipper	5
Phy.pha	Nymphalidae	Nymphalinae	*Phyciodes*	*phaon*	Phaon crescent	5
Ana.jat	Nymphalidae	Nymphalinae	*Anartia*	*jatrophae*	White peacock	4
Ech.iso	Lycaenidae	Polyommatinae	*Echinargus*	*isola*	Reakirts blue	4
Her.bip	Crambidae	Spilomelinae	*Herpetogramma*	*bipunctalis*	Southern beet webworm moth	4
Pyr.alb	Hesperiidae	Pyrginae	*Pyrgus*	*albescens*	White checkered-skipper	4
Dan.ple	Nymphalidae	Danainae	*Danaus*	*plexippus*	Monarch butterfly	3
Dry.iul	Nymphalidae	Heliconiinae	*Dryas*	*iulia*	Julia Heliconian	3
Hel.lav	Hesperiidae	Pyrginae	*Heliopetes*	*laviana*	Laviana white-skipper	3
Ler.acc	Hesperiidae	Hesperiinae	*Lerema*	*accius*	Clouded skipper	3
Moc.dis	Erebidae	Erebinae	*Mocis*	*disseverans*	Yellow mocis	3
Nyc.nyc	Hesperiidae	Hesperiinae	*Nyctelius*	*nyctelius*	Violet-banded skipper	3
Pap.pol	Papilionidae	Papilioninae	*Papilio*	*polyxenes*	Black swallowtail	3
Pho.sen	Pieridae	Coliadinae	*Phoebis*	*sennae*	Cloudless sulphur	3
Rin.cyd	Ennominae	Geometridae	*Ringdea*	*cyda*	Mesquite looper moth	3
Amb.cel	Hesperiidae	Hesperiinae	*Amblyscirtes*	*celia*	Celia's roadside-skipper	2
Amy.bul	Noctuidae	Bagisarinae	*Amyna*	*bullula*	Hook-tipped amyna	2
Bag.rep	Noctuidae	Bagisarinae	*Bagisara*	*repanda*	Wavy lined mallow moth	2
Bre.exi	Lycaenidae	Polyommatinae	*Brephidium*	*exilis*	Western pygmy-blue	2
Cym.odi	Hesperiidae	Hesperiinae	*Cymaenes*	*trebius*	Fawn-spotted skipper	2
Ean.thr	Hesperiidae	Pyrginae	*Eantis*	*thraso*	Sickle-winged skipper	2
Ela.spp	Noctuidae	Noctuinae	*Elaphria*	sp.	Midgets	2

Eup.ves	Hesperiidae	Hesperiinae	*Euphyes*	*vestris*	Dun skipper	2
Hem.sco	Erebidae	Boletobiinae	*Hemeroplanis*	*scopulepes*	Variable tropic	2
Her.sp.	Crambidae	Spilomelinae	*Herpetogramma*	sp.	Webworm moth	2
Kri.lys	Pieridae	Coliadinae	*Kricogonia*	*lyside*	Lyside sulphur	2
Ler.euf	Hesperiidae	Hesperiinae	*Lerodea*	*eufala*	Eufala skipper	2
Mel.ind	Erebidae	Erebinae	*Melipotis*	*indomita*	Indomitable melipotis	2
Mes.amy	Nymphalidae	Biblidinae	*Mestra*	*amymone*	Common mestra	2
Moc.tex	Erebidae	Erebinae	*Mocis*	*texana*	Texas mocis	2
Pan.oco	Hesperiidae	Hesperiinae	*Panoquina*	*ocola*	Ocola skipper	2
Van.ata	Nymphalidae	Nymphalinae	*Vanessa*	*atalanta*	Red admiral	2
Van.vir	Nymphalidae	Nymphalinae	*Vanessa*	*virginiensis*	American painted lady	2
Ach.alb	Hesperiidae	Eudaminae	*Achalarus*	*albociliatus*	Skinner's cloudywing	1
Ach.ran	Crambidae	Pyraustinae	*Achyra*	*rantalis*	Garden webworm	1
Agr.van	Nymphalidae	Heliconiinae	*Agraulis*	*vanillae*	Gulf fritillary	1
Amo.ros	Erebidae	Erebinae	*Amolita*	*roseola*	Owlet moth	1
Ani.ill	Noctuidae	Noctuinae	*Anicla*	*illapsa*	Snowy dart	1
Ani.sim	Noctuidae	Noctuinae	*Anicla*	*simplicius*	Simplicius dart moth	1
Ano.pri	Erebidae	Scoliopteryginae	*Anomis*	*privata*	Hibiscus-leaf caterpillar moth	1
Pyr.ara	Hesperiidae	Pyrginae	*Apyrrothrix*	*araxes*	Dull firetip	1
Bat.phi	Papilionidae	Papilioninae	*Battus*	*philenor*	Pipevine swallowtail	1
Ble.san	Erebidae	Herminiinae	*Bleptina*	*sangamonia*	Owlet moth	1
Cal.eth	Hesperiidae	Hesperiinae	*Calpodes*	*ethlius*	Brazilian skipper	1
Cat.lin	Noctuidae	Oncocnemidinae	*Catabena*	*lineolata*	Five-lined sallow	1
Cat.ala	Erebidae	Erebinae	*Catocala*	*alabamae*	Alabama underwing	1
Cen.pet	Tortricidae	Tortricinae	*Cenopis*	*pettitana*	Maple-basswood leafroller moth	1
Cis.plu	Erebidae	Arctiinae	*Cisthene*	*plumbea*	Lead colored lichen moth	1
Cna.coc	Crambidae	Spilomelinae	*Cnaphalocrocis*	*cochrusalis*	Marasmia moth	1
Cop.min	Hesperiidae	Hesperiinae	*Copaeodes*	*minima*	Southern skipperling	1
Dec.per	Hesperiidae	Hesperiinae	*Decinea*	*percosius*	Double-dotted Skipper	1
Don.mel	Crambidae	Schoenobiinae	*Donacaula*	*melinellus*		1
Don.sor	Crambidae	Schoenobiinae	*Donacaula*	*sordidellus*		1
Dys.pun	Erebidae	Hypenodinae	*Dyspyralis*	*puncticosta*	Spot-edged Dyspyralis moth	1
Ela.fus	Noctuidae	Noctuinae	*Elaphria*	*fuscimacula*	Cutworm	1
Ela.nuc	Noctuidae	Noctuinae	*Elaphria*	*nucicolora*	Sugarcane midget	1
Eor.den	Crambidae	Crambinae	*Eoreuma*	*densella*	Wainscot grass-veneer	1

Ery.fun	Hesperiidae	Pyrginae	*Erynnis*	*funeralis*	Funereal duskywing	1
Eup.cla	Nymphalidae	Heliconiinae	*Euptoieta*	*claudia*	Variegated fritillary	1
Fru.sp.	Gelechiidae	Gelechiinae	*Frumenta*	sp.	Twirler moth	1
Hel.mac	Hesperiidae	Pyrginae	*Heliopetes*	*macaira*	Turks-cap white skipper	1
Her.sos	Nymphalidae	Satyrinae	*Hermeuptychia*	*sosybius*	Carolina satyr	1
Her.aeg	Crambidae	Spilomelinae	*Herpetogramma*	*aeglealis*	Serpentine webworm	1
Her.flu	Crambidae	Spilomelinae	*Herpetogramma*	*fluctuosalis*	Greater sweet potato webworm moth	1
Hom.ele	Pyralidae	Phycitinae	*Homeosoma*	*electella*	Sunflower moth	1
Hyp.min	Erebidae	Hypeninae	*Hypena*	*minualis*	Sooty bomolocha moth	1
Ida.obf	Geometridae	Sterrhinae	*Idaea*	*obfusaria*	Rippled wave	1
Iso.ten	Erebidae	Boletobiinae	*Isogona*	*tenuis*	Thin-lined owlet	1
Lac.sub	Lacturidae		*Lactura*	*subfervens*	Speckled lactura	1
Leu.inc	Noctuidae	Noctuinae	*Leucania*	*incognita*	Wainscot moth	1
Lob.per	Geometridae	Sterrhinae	*Lobocleta*	*peralbata*		1
Mac.bic	Geometridae	Ennominae	*Macaria*	*bicolorata*	Bicolored angle	1
Mim.ruf	Crambidae	Odontiinae	*Mimoschinia*	*rufofascialis*	Rufous-banded crambid moth	1
Nat.iol	Papilionidae	Pierinae	*Nathalis*	*iole*	Dainty sulphur	1
Noc.pro	Noctuidae	Noctuinae	*Noctua*	*pronuba*	European yellow underwing	1
Nom.nea	Psychodomorpha	Psychodidae	*Nomophila*	*nearctica*	American celery webworm	1
Pal.fle	Crambidae	Pyraustinae	*Palpita*	*flegia*	Satin white palpita moth	1
Pal.qua	Crambidae	Spilomelinae	*Palpita*	*quadristigmalis*	Four-spotted palpita moth	1
Pan.rep	Erebidae	Eulepidotinae	*Panopoda*	*repanda*	Orange panopoda	1
Pho.aga	Pieridae	Coliadinae	*Phoebis*	*agarithe*	Large orange sulphur	1
Pho.phi	Pieridae	Coliadinae	*Phoebis*	*philea*	Orange-barred sulphur	1
Pol.fla	Crambidae	Pyraustinae	*Polygrammodes*	*flavidalis*	Ironweed root moth	1
Pon.pro	Pieridae	Pierinae	*Pontia*	*protodice*	Checkered white	1
Psa.aby	Geometridae	Ennominae	*Psamatodes*	*abydata*	Dot-lined angle	1
Pse.san	Crambidae	Pyraustinae	*Pseudopyrausta*	*santatalis*	Crambid snout moth	1
Pyr.sp.	Crambidae	Pyraustinae	*Pyrausta*	sp.	Crambid snout moths	1
Pyr.com	Hesperiidae	Pyrginae	*Pyrgus*	*communis*	Common checkered-skipper	1
Pyr.pro	Pieridae	Coliadinae	*Pyrisitia*	*proterpia*	Tailed orange	1
Ren.sp.	Erebidae	Herminiinae	*Renia*	sp.	Erebid moths	1
Rin.sig	Ennominae	Geometridae	*Rindgea*	*s-signata*	Signate looper moth	1
Sch.sp.	Erebidae	Hypenodinae	*Schrankia*	sp.	Schrankia	1
Sco.lim	Geometridae	Sterrhinae	*Scopula*	*limboundata*	Large lace-border	1

Sco.umb	Geometridae	Sterrhinae	*Scopula*	*umbilicata*	Swag-lined wave	1
Str.ist	Lycaenidae	Theclinae	*Strymon*	*istapa*	Mallow Scrub-Hairstreak	1
Str.mel	Lycaenidae	Theclinae	*Strymon*	*melinus*	Gray hairstreak	1
Van.car	Nymphalidae	Nymphalinae	*Vanessa*	*cardui*	Painted lady	1
Vid.per	Hesperiidae	Hesperiinae	*Vidius*	*perigenes*	Pale-rayed Skipper	1
Vir.sp.	Erebidae	Arctiinae	*Virbia*	sp.	Virbia	1
Lymantriinae	Erebidae	Lymantriinae			Tussock moth	1
Erebidae	Erebidae				Erebid moths	1
Scythrididae	Scythrididae				Flower moths	1
