# Supplementary material for: Effects of Patch Size, Fragmentation, and Invasive Species on Plant and Lepidoptera Communities in Southern Texas
Source: Insects. 2021 Aug 29;12(9):777. doi: 10.3390/insects12090777 (PMC8472066; doi:10.3390/insects12090777)
Supplement: Supplementary file 1 [file insects-12-00777-s001.zip › Table S3.pdf]

Effects of patch size, fragmentation, and invasive species on plant and Lepidoptera communities in southern Texas

James A. Stilley and Christopher A. Gabler

Table S3. Permutational multiple analysis of covariance (PerMANCOVA) results examining the effects of time category (morning or afternoon), rain category (yes or no during survey), edge to interior ratio, temperature, blooming plant cover, blooming plant encounter rate, the natural log of the ratio of native to IEP plant encounter rates, IEP plant encounter rate, and IEP plant cover on Lepidoptera community composition. More complex models with additional terms and interactions between terms were considered prior to model pruning. Environmental factors not included here can be interpreted as being insignificant.

**Factor**	**d.f.**	$F_{11,50}$	p	
Time category	1	0.60	0.9485	
Rain category	1	1.45	0.0550	.
Edge to interior ratio	1	2.62	0.0003	***
Temperature	1	1.77	0.0100	**
Blooming plant cover	1	1.62	0.0083	**
Blooming plant enc. rate	1	1.20	0.2237	
ln(native:IEP encounters)	1	1.94	0.0040	**
IEP plant enc. rate	1	1.08	0.3572	
IEP plant cover	1	1.47	0.0528	.
Model	9			
