# Supplementary material for: Effects of Patch Size, Fragmentation, and Invasive Species on Plant and Lepidoptera Communities in Southern Texas
Source: Insects. 2021 Aug 29;12(9):777. doi: 10.3390/insects12090777 (PMC8472066; doi:10.3390/insects12090777)
Supplement: Supplementary file 1 [file insects-12-00777-s001.zip › Table S4.pdf]

Effects of patch size, fragmentation, and invasive species on plant and Lepidoptera communities in southern Texas

James A. Stilley and Christopher A. Gabler

Table S4. Type III ANCOVA results examining the effects of habitat class, the natural log of patch size, edge to interior ratio, and woody plant encounter rate on the natural log of the ratio of native to IEP plant encounter rates, which is abbreviated as ln(Native:IEP encounters) in the main text.

**Factor**	**d.f.**	$F_{6,20}$	p	
Habitat class	3	9.95	0.0009	***
ln(Patch size)	1	1.99	0.1800	
Edge to interior ratio	1	1.08	0.3168	
Woody plant enc. rate	1	0.26	0.6194	
Model	6	5.53	0.0040	**
