# Supplementary material for: Effects of Patch Size, Fragmentation, and Invasive Species on Plant and Lepidoptera Communities in Southern Texas
Source: Insects. 2021 Aug 29;12(9):777. doi: 10.3390/insects12090777 (PMC8472066; doi:10.3390/insects12090777)
Supplement: Supplementary file 1 [file insects-12-00777-s001.zip › Table S5.pdf]

Effects of patch size, fragmentation, and invasive species on plant and Lepidoptera communities in southern Texas

James A. Stilley and Christopher A. Gabler

**Table S5.** Type III ANCOVA results examining the effects of habitat class, the natural log of patch size, edge to interior ratio, woody plant encounter rate, the natural log of the ratio of native to IEP plant encounter rates, IEP plant encounter rate, and IEP plant cover on plant richness.

| Factor | d.f. | $F_{9,20}$ | *p* | |
|---|---|---|---|---|
| Habitat class | 3 | 0.91 | 0.4655 | |
| ln(Patch size) | 1 | 0.13 | 0.7302 | |
| Edge to interior ratio | 1 | 7.49 | 0.0194 | * |
| Woody plant enc. rate | 1 | 2.65 | 0.1318 | |
| ln(Native:IEP enc. ratio) | 1 | 35.04 | 0.0001 | *** |
| IEP plant enc. rate | 1 | 41.14 | <0.0001 | *** |
| IEP plant cover | 1 | 0.18 | 0.6810 | |
| Model | 9 | 12.79 | 0.0001 | *** |
