# Supplementary material for: Effects of Patch Size, Fragmentation, and Invasive Species on Plant and Lepidoptera Communities in Southern Texas
Source: Insects. 2021 Aug 29;12(9):777. doi: 10.3390/insects12090777 (PMC8472066; doi:10.3390/insects12090777)
Supplement: Supplementary file 1 [file insects-12-00777-s001.zip › Table S6.pdf]

Effects of patch size, fragmentation, and invasive species on plant and Lepidoptera communities in southern Texas

James A. Stilley and Christopher A. Gabler

Table S6. Type III ANCOVA results examining the effects of habitat class, the natural log of patch size, edge to interior ratio, woody plant encounter rate, the natural log of the ratio of native to IEP plant encounter rates, IEP plant encounter rate, and IEP plant cover on total plant cover.

**Factor**	**d.f.**	$F_{9,20}$	p	
Habitat class	3	7.73	0.0047	**
ln(Patch size)	1	0.11	0.7490	
Edge to interior ratio	1	0.06	0.8164	
Woody plant enc. rate	1	0.08	0.7802	
ln(Native:IEP enc. ratio)	1	28.14	0.0003	***
IEP plant enc. rate	1	2.44	0.1466	
IEP plant cover	1	17.53	0.0015	**
Model	9	10.39	0.0003	***
