# Supplementary material for: Effects of Patch Size, Fragmentation, and Invasive Species on Plant and Lepidoptera Communities in Southern Texas
Source: Insects. 2021 Aug 29;12(9):777. doi: 10.3390/insects12090777 (PMC8472066; doi:10.3390/insects12090777)
Supplement: Supplementary file 1 [file insects-12-00777-s001.zip › Table S7.pdf]

Effects of patch size, fragmentation, and invasive species on plant and Lepidoptera communities in southern Texas

James A. Stilley and Christopher A. Gabler

Table S7. Type III ANCOVA results examining the effects of habitat class, the natural log of patch size, edge to interior ratio, woody plant encounter rate, the natural log of the ratio of native to IEP plant encounter rates, IEP plant encounter rate, and IEP plant cover on plant diversity (Shannon *H'*).

**Factor**	**d.f.**	$F_{9,20}$	p	
Habitat class	3	1.97	0.1770	
ln(Patch size)	1	2.02	0.1827	
Edge to interior ratio	1	0.20	0.6646	
Woody plant enc. rate	1	0.35	0.5656	
ln(Native:IEP enc. ratio)	1	8.26	0.0151	*
IEP plant enc. rate	1	46.78	<0.0001	***
IEP plant cover	1	16.84	0.0017	**
Model	9	11.09	0.0002	***
